# Molecular sexing assays in 114 mammalian species: In silico sequence reanalysis and a unified graphical visualization of diagnostic tests

**DOI:** 10.1002/ece3.5093

**Published:** 2019-04-08

**Authors:** Rebeka Strah, Tanja Kunej

**Affiliations:** ^1^ Biotechnical Faculty, Department of Animal Science University of Ljubljana Domzale Slovenia

**Keywords:** mammals, molecular sexing, PCR, sex determination, sex identification

## Abstract

Molecular‐based methods for identifying sex in mammals have a wide range of applications, from embryo manipulation to ecological studies. Various sex‐specific or homologous genes can be used for this purpose, PCR amplification being a common method. Over the years, the number of reported tests and the range of tested species have increased greatly. The aim of the present analysis was to retrieve PCR‐based sexing assays for a range of mammalian species, gathering the gene sequences from either the articles or online databases, and visualize the molecular design in a uniform manner. For nucleotide alignment and diagnostic test visualization, the following genomic databases and tools were used: NCBI, Ensembl Nucleotide BLAST, ClustalW2, and NEBcutter V2.0. In the 45 gathered articles, 59 different diagnostic tests based on eight different PCR‐based methods were developed for 114 mammalian species. Most commonly used genes for the analysis were *ZFX*, *ZFY*, *AMELX*, and *AMELY*. The tests were most commonly based on sex‐specific insertions and deletions (SSIndels) and sex‐specific sequence polymorphisms (SSSP). This review provides an overview of PCR‐based sexing methods developed for mammals. This information will facilitate more efficient development of novel molecular sexing assays and reuse of previously developed tests. Development of many novel and improvement of previously developed tests is also expected with the rapid increase in the quantity and quality of available genetic information.

**Main findings of the study**

1. Overview of 59 molecular sexing tests in 114 species from 45 publications and unified graphical visualization based on nucleotide sequence.
2. Sequence reanalysis of previously developed molecular sexing tests and their update according to recent genomic information.
3. Proposed three main elements necessary for the development of new PCR‐based molecular sexing assays: primer design, product size, and internal amplification controls.

## 1 INTRODUCTION

Molecular‐based sexing techniques can be used to reliably determine sex in mammals with limited sexual dimorphism. However, even in species with clear sexually dimorphic traits molecular sexing has various purposes, such as embryo sex identification, behavior and ecology studies, and conservation genetics.

For molecular‐based sexing, sex‐specific DNA markers are often utilized, such as the presence of a testis‐determining factor gene (*SRY*) in mammals. In our previous study, we reviewed various molecular‐based sexing methods and proposed terminology unification regarding sex‐specific sequence variants (SSSV) (Hrovatin & Kunej, 2018). Those can further be divided into three main groups: (a) length polymorphisms, (b) sequence differences, and (c) number (dose) of sex chromosomes. Length differences can arise either due to chromosome specific number of repeats, or due to indels specific for either sex chromosome—sex‐specific indels (SSIndel). Sequence differences encompass Y‐chromosome‐specific fragments or genes, allele‐specific sequences (nonhomologous parts of homologous genes), and single nucleotide variations on homologous genes of sex chromosomes (sex‐specific sequence polymorphisms—SSSP).

We also established minimal requirements for reporting molecular sexing assays, including unification terminology (Hrovatin & Kunej, 2018): species scientific name, species ID, gene name, sequence and ID, sex‐specific variant, method, coordinates of relevant regions on the nucleotide sequence, characteristics defining the amplicon system, description of detected amplicons and controls, and reference PMID or WoS ID. There is, however, still little overview of the currently existing molecular sexing assays, based on PCR, which are still the most commonly used. The field lacks a review study on existing sexing methods developed for different species, as a consequence multiple tests have been developed for the same species. While many new tests are published, previously developed tests have not yet been reexamined according to the recent updates of genomic browsers. Additionally, in many of the examined articles the methods were not adequately described and the information needed to be supplemented. Finally, the main elements for development of a PCR‐based molecular sexing test need to be summarized for more efficient development of the study in the future.

The aim of the present analysis was therefore to: (a) gather reported PCR‐based sexing assays for a range of mammalian species and develop a table with extracted relevant information from the publications, (b) supplement the extracted data with missing genomic information, (c) reexamine the molecular design using data from latest genomic browsers using in silico analysis, (d) unify graphical visualizations of the sexing tests, and (e) summarize main elements for designing and reporting a PCR‐based sexing test.

## 2 MATERIALS AND METHODS

To retrieve the articles, we used the following key words: «PCR molecular sexing mammals, PCR molecular sexing mammals AMEL*, PCR sex identification mammal, PCR molecular sex determination mammal, PCR sexing mammal, PCR sexing mammal amelogenin, mammal sexing *ZFX,* and mammal sexing *ZFY*« in Web of Science (https://webofknowledge.com/), the PubMed NCBI citation database (https://www.ncbi.nlm.nih.gov/pubmed/), and the Google scholar (https://scholar.google.si/). We conducted the searches in November 2016 and in November 2018. The time span for literature search was from 1990 to November 2018. We excluded articles describing non‐PCR sexing methods and not written in English.

Information extracted from the articles was entered into a tabular format. Scientific names for the species were complemented if missing in the source reference. Old gene names were unified according to the HGNC database (https://www.genenames.org/). In cases where gene names were not found in the HGNC database and other sequences used were not named with a gene name, we kept the nomenclature used by the authors. The NCBI taxonomy browser (https://www.ncbi.nlm.nih.gov/taxonomy/) was used to acquire the taxonomy ID and the common tree tool was used to arrange them in a taxonomical order. The nucleotide sequence alignments used for visualizations of homologous regions of the X and Y chromosomes were produced by using either Nucleotide BLAST (https://blast.ncbi.nlm.nih.gov/Blast.cgi) or ClustalW (http://www.ebi.ac.uk/Tools/msa/clustalw2/). In case of missing NCBI accession numbers (NCBI acc. no.), Ensembl and NCBI databases were searched based on the data that were provided: gene names, representations of alignments and polymorphisms, and matching cited primers to candidate gene sequences.

Visualization of the molecular sexing tests was performed using the following steps. The Nucleotide BLAST was used for the majority of alignments, and ClustalW was used in cases of large gaps in the sequences. Genetic polymorphisms were extracted from Ensembl browser and marked on the sequence. For tests including the use of a restriction enzyme, the enzyme recognition sites of the sequences were retrieved using the NEBCutter v2.0 tool (http://nc2.neb.com/NEBcutter2/).

Ensembl genomic browser release 90 was used to retrieve information on genetic variations (Zerbino et al., 2018). In cases of PCR assays based on nonhomologous genes, chromosome ideograms and locations of the genes were extracted from Ensembl browser. In cases of references with incomplete information related with nucleotide sequences, we visualized the method with a simple sketch of the sequence, primers, and the SSSV. We presented the expected results for each method with a visualization of band lengths in bp on an agarose gel.

## 3 RESULTS

The present analysis consisted of the following five main steps: (a) obtaining articles on molecular sexing of mammals and extracting the available data, (b) complementing the missing genomics data and presentation in a tabular format, (c) obtaining SNP locations from the Ensembl browser, (d) visualization of the assays in a unified manner, and (e) summing up the main elements and guidelines for designing a new PCR‐based test for molecular sexing.

### 3.1 Literature search and data extraction

Obtained 45 articles were published between 1990 and 2018. A total of 114 different species were sexed in these articles. Several assays were tested on multiple species, giving a total of 161 tests. The articles were heterogeneous in terms of the information they provided. Most did not report species ID, gene accession numbers or sample sizes, but sometimes also lacked electrophoreograms or any product sizes in base pairs.

### 3.2 Complementing the missing data and tabular presentation

The data extracted from the articles are presented in tabular format (Table 1). For each test, the following information is presented: common name and scientific name of the species, taxonomy ID, gene name, SSSV, sample size, and method. Additional details are included in the Supporting Information Appendix S1: gene name, primer name, nucleotide sequences of the forward and reverse primer, and annealing temperatures for PCR.

**Table 1:** Representation of the tests presented in the article, arranged in order of species based on NCBI taxonomy. The table contains genes and SSSVs used, sample sizes (if available), and method used

Common name (*Scientific name*)	Taxonomy ID	Gene or marker name	SSSV	Sample size	Method	Citation
Human (*Homo sapiens)*	9606	*AMELX, AMELY*	SSIndel	22	PCR	Faerman et al., (1995)
Human (*Homo sapiens)*	9606	*SRY, ATL1 marker*	*SRY*‐ 198 bp *ATL1* ‐ 261 bp		PCR	Tungwiwat et al., (2003)
Human (*Homo sapiens)*	9606	*ZFX, ZFY*	SSSP		PCR‐RFLP	Aasen and Medrano, (1990)
Human (*Homo sapiens)*	9606	*AMELX, AMELY, ZFX, ZFY*	SSIndel		PCR	Fredsted and Villessen, (2004)
Human (Homo sapiens)	9606	*AMELX, AMELY*	SSIndel		PCR	Gibbon et al., (2009)
Apes: Human (*Homo sapiens*), chimpanzee (*Pan troglodytes*), gorilla (*Gorilla gorilla*), orangutan (*Pongo pygmaeus*)	9606, 9598 9593, 9600	*ZFX, ZFY*	SSIndel	129, 6, 6, 3. respectively	PCR	Wilson and Erlandsson, (1998)
Apes: Human (*Homo sapiens),*Chimpanzee (*Pan troglodytes),*Gorilla (*Gorilla gorilla),*Orangutan (*Pongo pygmaeus),*White‐cheeked gibbon (*Nomascus leucogenys)*	9606, 9598 9593, 9600 61853	*DDX3X, DDX3Y*	SSIndel		PCR	Villesen and Fredsted, (2006)
Old world monkeys: Rhesus macaque (*Macaca mulatta),*Hamadryas baboon (*Papio hanadryas),*Colobus monkey *(Colobus guereza),*Douc langur *(Pygathrix nemaeus)*	9544, 9557 33548, 54133	*DDX3X, DDX3Y*	SSIndel		PCR	Villesen and Fredsted, (2006)
Baboon (*Papio*)	9554	*ZFX, ZFY*	SSIndel		PCR	Wilson and Erlandsson, (1998)
Pig‐tailed macaque (*Macaca nemestrina),*Japanese macaque *(Macaca fuscata),*crab‐eating macaque *(Macaca fascicularis),* Rhesus macaque (*Macaca mulatta)*	9545, 9542 9541, 9544	*AMELX, AMELY*	SSIndel		PCR	Morrill et al., (2008)
Tonkean macaque (*Macaca tonkeana)*	40843	*ZFX, ZFY*	SSSP	4	PCR‐RFLP	Fernando and Melnick, (2001)
Mandrill (*Mandrillus sphinx*)	9561	*AMELX, AMELY*	SSIndel		PCR	Morrill et al., (2008)
New world monkeys: Marmaoset (C*allithrix jacchus),*Bolivian squirrel monkey *(Saimiri boliviensis),*Brown capuchin *(Cebus apella),*Spider monkey *(Ateles fusciceps*)	9483, 27679 9515, 9508	*DDX3X, DDX3Y*	SSIndel		PCR	Villesen and Fredsted, (2006)
Marmoset (C*allithrix jacchus*)	9483	*ZFX, ZFY*	SSSP		PCR‐RFLP	Takabayashi and Katoh, (2011)
Marmoset (C*allithrix jacchus*)	9483	*ZFX, ZFY*	SSIndel		PCR	Wilson and Erlandsson, (1998)
Prosimians: Gray mouse lemur (*Microcebus murinus),*Berthe's mouse lemur *(Microcebus berthae),*Lesser dwarf lemur *(Cheirogaleus medius),*Red‐fronted lemur *(Eulemur fulvus rufus),*Coquerel's mouse lemur *(Mirza coquereli),* red‐tailed sportive lemur *(Lepilemur ruficaudatus),* Ring‐tailed lemur *(Lemur catta*)	30608 143352, 9460 859983, 47180, 78866 9447	*AMELX, AMELY, ZFX ZFY*	SSIndel		PCR	Fredsted and Villessen, (2004)
Mouse (*Mus musculus*)	10090	*Sly, Xlr*	SSIndel		PCR	McFarlane et al., (2013)
Mouse (*Mus musculus*)	10090	*Kdm5c, Kdm5d*	SSIndel		PCR	Clapcote and Roder, (2005)
Rat (*Rattus norvegicus)*	10116	*Sry, Actb*			Duplex PCR	Miyajima et al., (2009)
Rabbits and hares (Leporidae: *Oryctolagus cuniculus, Lepus europaeus, Lepus timidus*)	9986, 9983 62621	*ZFX, ZFY*	SSSP	131 in total (70 european rabbits, 37 brown hares, 24 mountain hares)	PCR‐RFLP	Fontanesi et al., (2008)
Lesser horseshoe bat (*Rhinolophus hipposideros*)	77218	*DDX3X, DDX3Y*	SSIndel	39	PCR	Zarzoso‐Lacoste et al., (2018)
Silver‐haired bat (L*asionycteris noctivagans*), eastern red bat, (*Lasiurus borealis*), hoary bat, (*Lasionycteris cinereus),*evening bat (*Nycticeius humeralis*), tri‐colored bat (*Perimyotis subflavus*), Mexican freetailed bat (*Tadarida brasiliensis*)	27667, 258930, 257879, 27670, 27672, 9438	*ZFX, ZFY*	SSSP	924	Duplex PCR	Korstian et al., (2013)
Felidae: Wild cat (*Felis silvestris),*Bobcat *(Lynx rufus),*Eurasian lynx *(Lynx lynx),*Puma *(Puma concolor)*	9683, 61384, 13125, 9696	*ZFX, ZFY, AMELX, AMELY*	SSIndel	100	PCR	Pilgrim et al., (2005)
Puma (*Puma concolor*), Pallas's cat (*Otocolobus manul*) jaguar (*Panthera onca*), tiger (*Panthera tigris*), lion (*Panthera leo*), serval (*Leptailurus serval*), bobcat (*Lynx rufus*)	9696, 61408, 9690, 9694, 9689, 61405, 61384	*ZFX, SRY*		48, 25, 4, 2, 1 and 1, respectively		DeCandia et al. (2016)
Masked palm civet (*Paguma larvata*)	9675	*ZFX, SRY*		8	Duplex PCR	Zhang et al., (2016)
Otter (*Enhydra lutris*)	34882	*ZFX, ZFY*	SSSP	328	PCR‐RFLP	Hattori et al., (2003)
Mediterranean monk seals (*Monachus monachus*), Hawaiian monk seals (*Monachus schauinslandi*)	248254, 29088	*ZFX, SRY*		72 and 10, respectively	Duplex PCR	DeCandia et al. (2016)
Domestic dog (*Camis lupus familiaris*), coyote (*Canis latrans*)	9615, 9614	*ZFX, SRY*		1 and 2, respectively	Duplex PCR	DeCandia et al. (2016)
Dog (*Canis lupus familiaris*)	9615	*AMELX, AMELY*	SSIndel	128	PCR	Yan et al., (2013)
Dog (*Canis lupus familiaris*)	9615	*ZFX, ZFY*	SSSP		PCR‐RFLP	Ortega et al., (2004)
Dog (*Canis lupus familiaris*)	9615	*ZFX, ZFY*	SSSP	4	PCR‐RFLP	Fernando and Melnick, (2001)
Wolf (*Canis lupus*)	9612	*DDX3Y,*chr‐X marker amplified by primer AHTx40		153	PCR	Sastre et al., (2009)
Coyote (*Canis latrans*)	9614	*ZFX, ZFY*	SSSP		PCR‐RFLP	Ortega et al., (2004)
Maned wolf (*Chrysocyon brachyurus*)	68728	*ZFX, ZFY*	SSSP		PCR‐RFLP	Ortega et al., (2004)
Gray fox (*Urocyon cinereoargenteus*), red fox, (*Vulpes vulpes*) San Joaqion kit fox (*Vulpes marcotis mutica‐*no taxonomy ID)	55040, 9627	*ZFX, ZFY*	SSSP	354	PCR‐RFLP	Ortega et al., (2004)
Brown bear (*Ursus arctos),*Polar bear *(Ursus maritimus),*American black bear *(Ursus americanus),*Asian black bear *(Ursus thibetanus),*sun bear *(Helarctos malayanus),*Sloth bear *(Melursus ursinus),*Spectacled bear *(Tremarctos ornatus)*	9644, 29073, 9643, 9642, 9634, 9636, 9638	*SMCY, 318.2*Y‐linked marker*, ZFX*			multiplex PCR	Bidon et al., (2013)
Giant panda (*Ailuropoda melanoleuca*), brown bear (*Ursus arctos*), sloth bear (*Melursus usinus*), spectacled bear (*Tremarctos ornatus*)	9646, 9644, 9636, 9638	*ZFX, ZFY*	SSSP	7 giant pandas, 1 ursus arctos, 3 sloth bears, 5 spectacled bears	allele‐specific PCR	Durnin et al., (2007)
Red panda (*Ailurus fulgens)*	9649	*AMELX, AMELY*	SSIndel	22	PCR	Kumar et al. (2015)
Racoon (*Procyon lotor*)	9654	*ZFX, ZFY*	SSSP		Duplex PCR	Okuyama et al., (2014)
European red deer (*Cervus elaphus*)	9860	*AMELX, AMELY*	SSIndel		PCR	Pfeiffer and Brenig, (2005)
White‐tailed deer (*Odocoileus virginianus*)	9874	*ZFX, ZFY*	SSIndel		PCR	Lindsay & Belant (2007)
Cattle (*Bos taurus*)	9913	*AMELX, AMELY*	SSIndel		PCR	Chen et al., (1999)
Cattle (*Bos taurus*)	9913	*AMELX, AMELY*	SSIndel	28	PCR	Gokulakrishnan et al., (2013)
Cattle (*Bos taurus*)	9913	*DDX3X, DDX3Y*	SSIndel	28	PCR	Gokulakrishnan et al., (2012)
Cattle (*Bos taurus*)	9913	*ZFX, ZFY*	SSSP		PCR‐RFLP	Aasen and Medrano, (1990)
Water buffalo (*Bubalus bubalis)*	89462	*ZFX, ZFY*	SSSP		PCR‐RFLP	Pande and Totey, (1998)
Goat (*Capra hircus*)	9925	*AMELX, AMELY*	SSSP	43	allele‐specific PCR	Tsai et al., (2011)
Goat (*Capra hircus*)	9925	*AMELX, AMELY*	SSIndel	28	PCR	Gokulakrishnan et al., (2013)
Goat (*Capra hircus*)	9925	*ZFX, ZFY*	SSSP		PCR‐RFLP	Aasen and Medrano, (1990)
Goat (*Capra hircus*)	9925	*AMELX, AMELY, SRY*	SSIndel	135	PCR	Malik et al., (2013)
Sheep (*Ovis aries*)	9940	*AMELX, AMELY*	SSIndel		PCR	Pfeiffer and Brenig, (2005)
Sheep (*Ovis aries*)	9940	*AMELX, AMELY*	SSIndel	28	PCR	Gokulakrishnan et al., (2013)
Sheep (*Ovis aries*)	9940	*ZFX, ZFY*	SSSP		PCR‐RFLP	Aasen and Medrano, (1990)
Odontocetes: Harbor porpoise (*Phocoena phocoena),*Narwhal *(Monodon monoceros),*Beluga *(Delphinapterus leucas),*Mysticetes: Minke whale (*Balaenoptera acutorostrata),*Fin whale *(Balaenoptera physalus),*Blue whale *(Balaenoptera musculus),*Humpback whale *(Megaptera novaeangliae)*	9742, 40151, 9749, 9767, 9770, 9771, 9773	*ZFX, ZFY*	SSSP	3,570 in all (2,284 humpback whales, 315 fin whales, 37 blue whales, 7 minke whales, 592 belugas, 335 narwhals, 25 harbor porpoises)	allele‐specific PCR	Berube and Palsboll, (1996)
Cetaceans: Bowhead whale (*Balaena mysticetus),*North Pacific right whale *(Eubalaena japonica),*Minke whale *(Balaenoptera acutorostrata),*Sei whale *(Balaenoptera borealis),*Pigmy Bryde's whale *(Balaenoptera edeni),*Blue whale *(Balaenoptera musculus),*Fin whale *(Balaenoptera physalis),*Humpback whale *(Megaptera novaeangliae),*long‐beaked common dolphin *(Delphinus capensis),*saddleback dolphin *(Delphinus delphis),*short‐finned pilot whale *(Globicephala macrorhynchus),*long‐finned pilot whale *(Globicephala melas),*Risso's dolphin *(Grampus griseus),*Fraser's dolphin *(Lagenodelphis hosei),*white‐beaked dolphin *(Lagenorhynchus albirostris),*pacific white‐sided dolphin *(Lagenorhynchus obliquidens),*northern light whale dolphin *(Lissodelphis borealis),*Killer whale *(Orcinus orca),*false killer whale *(Pseudorca crassidens),*brideld dolphin *(Stenella attenuata),*striped dolphin *(Stenella coeruleoalba),*rough‐toothed dolphin *(Steno bredanensis),*bottlenose dolphin *(Trusiops truncatus),*gray whale *(Eschrichtius robustus),*sperm whale *(Physeter macrocephalus),*pigmy sperm whale *(Kogia breviceps),*dwarf sperm whale *(Kogia sima),* beluga *(Delphinapterus leucas),*narwhal *(Monodon monoceros),*harbor porpoise *(Phocoena phocoena), (Phocoenoides dalli),*Blainville's beaked whale *(Mesoplodon densirostris),*Cuvier's beaked whale *(Ziphius cavirostris)*	27602, 302098, 9767, 9768, 9769, 9771, 9770, 9773, 103584, 9728, 38241, 9731, 83653, 103594, 27610, 90247, 103588, 9733, 82174, 9735, 9737, 46167, 9739, 9734, 9755, 27615, 9752, 9749, 40151, 9742, 9744, 48708, 9760	*ZFX, ZFY*	SSSP		qPCR	Morin et al., (2005)
Bottle‐nosed dolphin (*Tursiops truncatus*), bridled dolphin (*Stenella attenuata*), Clymene dolphin *(Stenella clymene*), striped dolphin (*Stenella coeruleoalba*), atlantic spotted dolphin (*Stenella frontalis*), spinner dolphin (*Stenella langirostris*), saddleback dolphin (*Delphinus delphis*), harbor porpoise (*Phocoena phocoena*), Boutu (*Inia geoffrensis*), false killer whale (*Pseudorca crassidens*)	9739, 9735, 103589, 9737, 103590, 9736, 9728, 9742, 9725, 82174	*ZFX, SRY*			duplex PCR	Rosel, (2003)
Pig (*Sus scrofa domesticus*)	9825	*AMELX, AMELY*	SSIndel	329 (287 known)	PCR	Fontanesi et al., (2008)
Pig (*Sus scrofa domesticus*)	9825	*ZFX, SRY*		345	duplex PCR	Blanes et al., (2016)
Hippopotamus *(Hippopotamus amphibious)*	*9833*	*ZFX, ZFY*	SSSP	60 (6 of known sex)	PCR‐RFLP	Beckwitt et al., (2002)
Horse (*Equus caballus*)	9796	*ZFX, ZFY*	SSIndel	128	PCR	Han et al., (2010)
Indian rhynoceros (*Rhinoceros unicornis*)	9809	*ZFX, ZFY*	SSSP	4	PCR‐RFLP	Fernando and Melnick, (2001)
Asian elephant (*Elephas maximus*)	9783	*ZFX, ZFY*	SSSP	4	PCR‐RFLP	Fernando and Melnick, (2001)
Asian elephant (*Elephas maximus*)	9783	*ZFX, ZFY*	SSSP	129	PCR‐RFLP	Vidya et al. (2003)
Pale‐throated sloth (*Bradypus tridactylus*), Brown‐throated sloth (*Bradypus variegatus*), Maned three‐toed sloth (*Bradypus torquatus*)	9354, 9355, 227087	*ZFX, ZFY*	SSSP	47	PCR‐RFLP	Martinelli et al., (2010)
Opossum: Brushtail						
(*Trichosurus vulpecula*), Ringtail (*Pseudocheirus peregrinus*)	9337, 9333	*SRY, G6DP*		66	multiplex PCR	Russell, (2011)

In total, 25 articles reported the sequences used for the assay development accompanied by NCBI accession numbers or Ensembl ID. For 21 articles, the sequences were not provided. Available sequences were obtained from genomics databases for 12 of the articles not containing NCBI accession numbers or Ensembl ID. Ten articles employed nonhomologous genes for their test, so sequence alignments were not necessary for visualization.

### 3.3 Visualizations of reanalyzed molecular sexing tests

**Figure 1:**
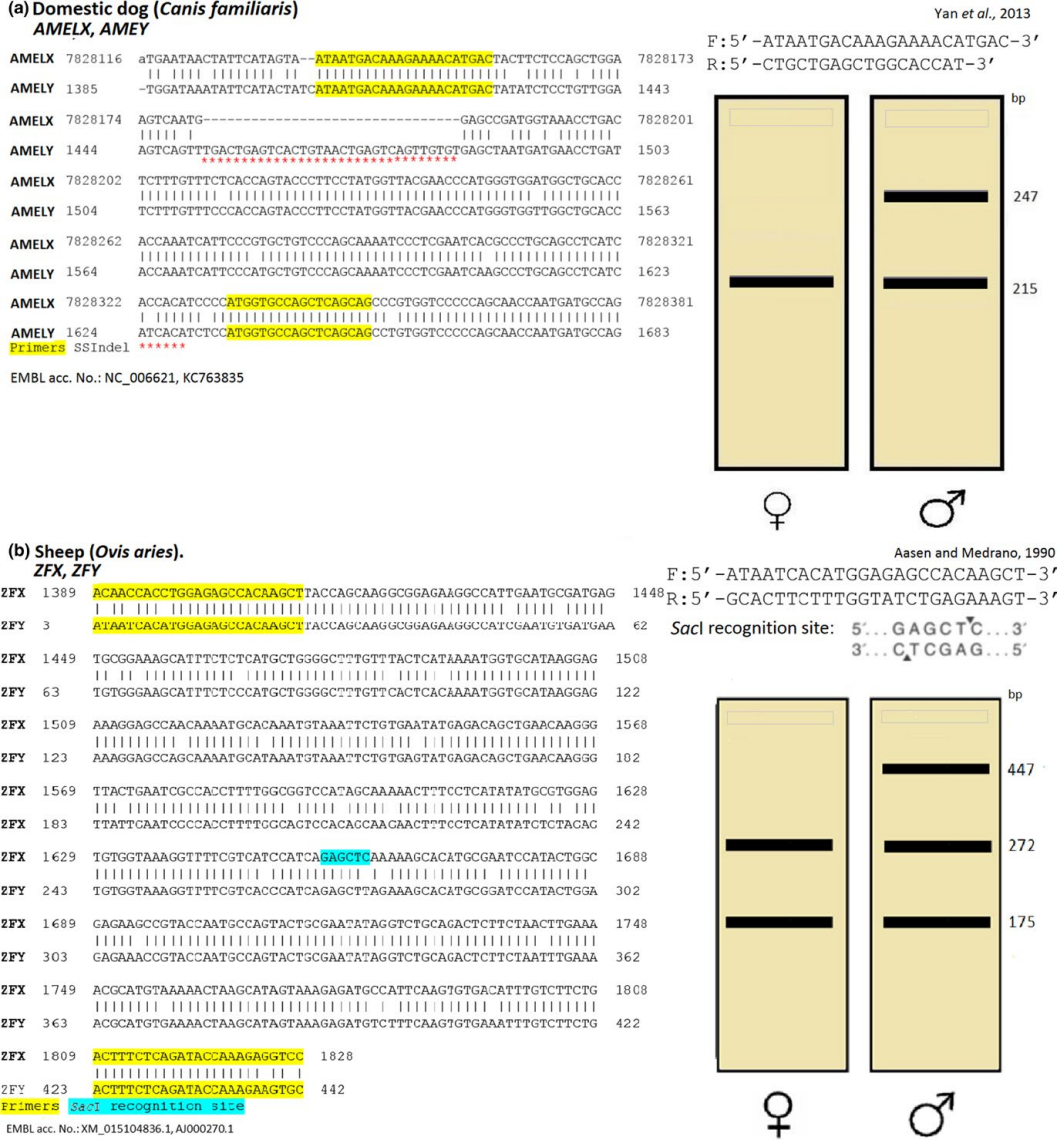
(a) A visual representation of the design of sex determination test using a PCR method for the domestic dog, containing an SSIndel (b) A visual representation of sex determination using a PCR‐RFLP method for sheep, containing an SSSP

Visualizations of 65 tests for 114 species are presented in Supporting Information Appendix S2, and two examples of visualized tests are also presented in Figure 1a,b. Visualization of each test includes the following elements: article citation, species common name, species scientific name, primers used, sequence alignment (or either chromosome or gene representation SSSV on the sequence, restriction enzyme recognition and cleavage sites (where appropriate), expected PCR products for both sexes and NCBI accession numbers or Ensembl ID.

### 3.4 Main elements required for development of a new molecular sexing test

In this section, we sum up minimal information for designing a PCR‐based sexing technique obtained from the articles. Generally, it is useful to obtain reliable genetic information on the species in question, genes and SSSVs. Ideally, the products should be amplified in one step, produce unambiguous results, and provide an internal amplification control (Villesen & Fredsted, 2006) The goal is to choose a method compatible with laboratory equipment and intended use. After obtaining the nucleotide sequence, the appropriate SSSV, method, and primer specificity are chosen based on the type and quality of the samples to be used in research. While designing the test, three basic elements should be considered.

#### 3.4.1 Primer design

Primers can be designed to either amplify genes of multiple species, or are specific for one species. The approach is chosen according to the purpose and the means of the study. Degenerate primers are useful for multiple species, while species‐specific primers are usually preferred for studies of samples collected in the field, which might be contaminated with foreign DNA. For example, Sastre et al. (2009) developed a test used on wolf fecal samples and tested it on several species of animals likely to be preyed upon by wolves, and Okuyama et al., 2014 designed a raccoon‐specific test, which would also prevent species misidentification of the samples collected in the wild.

Design of degenerate primers useful for a greater number of species usually targets genes commonly preserved between the species (Aasen & Medrano, 1990). Primers can be derived from a consensus sequence (Bidon et al., 2013; Fredsted & Villessen, 2004; Morin et al., 2005).

#### 3.4.2 Product size

Defining the optimal product size and size difference between the products is necessary for sexing and amplification success. Recommended length for PCR products is 300–800‐bp for good quality samples (Morin et al., 2005) and shorter than 170‐bp for degraded DNA samples prone to amplification failure (Durnin et al., 2007; Villesen & Fredsted, 2006). In PCR reactions containing degraded, low quality DNA smaller fragments are preferentially amplified (Faerman et al., 1995). Designing the Y‐specific amplicon to be smaller than the X‐specific amplicon is an approach to avoid this Y dropout (Bidon et al., 2013; Faerman et al., 1995; McFarlane et al., 2013; Wilson & Erlandsson, 1998).

#### 3.4.3 Internal amplification controls

Internal PCR amplification controls confirm successful amplifications and thus increase the reliability of the test. Often, X‐specific or autosomal products are utilized. They are necessary because absence of a male‐specific signal can be the result of an unsuccessful PCR reaction.

Usually, the Y‐specific product is the diagnostic component and the X‐specific (or autosomal) product is the amplification control. The amplification control is present in all samples and indicates a successful PCR reaction, while the presence or absence of the diagnostic (Y‐specific) product determines the sex. Bidon et al., 2013 even used amplification of two Y‐specific and independent genes (in addition to the amplification control) to decrease the possibility of one diagnostic Y‐chromosome signal not appearing due to failed amplification.

Tests which amplify homologous X‐ and Y‐specific genes with the same pair of primers already include the internal control. Nevertheless, an additional primer pair for a Y‐specific gene (mostly *SRY*) can still be included when developing a method, in order to corroborate the results (Lindsay & Belant, 2007; Malik et al., 2013; Morin et al., 2005).

## 4 DISCUSSION

The present analysis contains a collection of PCR‐based sexing assays for 114 mammalian species and presents the first sequence reanalysis of existing sexing tests using bioinformatics tools. The sexing tests are visualized in a unified manner, enabling better comparison of the tests. Results of the present study will allow more efficient development of novel tests and enable reuse of previously developed tests. The most commonly used method was simplex PCR, the most common gene *ZFX*and the most common SSSV an SSIndel. Accession numbers for sequences were provided in 25 articles. The sexing tests were presented in 65 separate visualizations.

While more than half of the articles (29 out of 45) reported sample sizes, they were often small. Larger sample sizes would contribute to greater reliability of the assays, especially ones that rely on SSSPs. Other information, such as NCBI accession numbers and gene names, was also lacking in some articles, making the search for relevant sequences laborious. Including this information would not only facilitate reuse of existing sexing assays on already tested species, but also help researchers applying the tests on other species. A unified way of presenting results of the development of sex identification assays, such as proposed by Hrovatin & Kunej, 2018, would greatly help make the field more manageable.

Our in silico reanalysis of the existing sexing assays shows that the presence of SNPs should also be considered while developing a new assay. Further studies are needed to test a possibility of SNPs discovered recently interfering an existing assay and expected results.

The present study contains a collection of information on a range of PCR‐based sexing test, enabling easier making the access to information on already existing assays, such as primers, genes, SSSVs, and expected results of specific tests. Missing information from the articles, such as official gene names and accession numbers for the sequences used for sexing, is also supplemented. The unified visualizations present sequence alignments of the PCR sexing assays and their expected results. To our knowledge, this study is the first to review and reanalyze the existing sexing assays. In future studies, it should be explored if sequence variants discovered recently effect previously developed sexing assays. The three main elements of designing a PCR‐based sexing assay presented in this study will help in the development of new tests where necessary.

While the application of bioinformatics methods for in silico development of new genetic sexing assays can help produce reliable tests in the future, the importance of confirmation with larger sample sizes should not be overlooked, due to the possibility of variation of the genes of interest within the population. The increase in availability of annotated genomic data (especially containing information on possible SSSPs and SSIndels) can, however, also help develop more reliable assays while at the same time decrease the necessity for large sample sizes, especially in cases where samples are not readily available. For better review of the existing and upcoming novel sexing assays, a searchable database should be developed.

## CONFLICT OF INTEREST

None declared.

## AUTHOR CONTRIBUTIONS

Data curation, data synthesis, visualization and writing R.S; design of the study, writing, coordination of the study: T.K.

## Data Availability

Sequences used for alignments were downloaded from NCBI and Ensembl, their accession numbers are provided in Supporting Information Appendix S1.
