# Supplementary material for: Molecular sexing assays in 114 mammalian species: In silico sequence reanalysis and a unified graphical visualization of diagnostic tests
Source: Ecol Evol. 2019 Apr 8;9(8):5018–28. doi: 10.1002/ece3.5093 (PMC6476764; doi:10.1002/ece3.5093)
Supplement: Supplementary file 1 [file ECE3-9-5018-s001.pdf]

Supplementary file 1: A table of primers used in the articles. Includes their target genes, names, sequences, annealing temperatures and species.

**Gene, locus**	**Primer name**	**Forward 5'-3'**	**Reverse 5'-3'**	**Anneal-ing T**	**Species**	**Citation**
ZFX/Y	P1-5EZ, P2-3EZ	ATAATCACATGGAGAGCCAC-AAGCT	GCACTTCTTTGGTATCTGAGAAA-GT	various	*Bradypus*, *Elephas maximus*, *Rhinoceros unicornis*, *Hippopotamus amphibians*, *Cetacea*, *Ovis aries*, *Capra hircus*, *Bos taurus*, *Procyon lotor*, *San Joaquin kit fox*, *coyote*, *gray fox*, *red fox*, *maned wolf*, *Canis lupus*, *Canis familiaris*, *Enhydra lutris*, *Macaca tonkeana*, *Homo sapiens*	Aasen & Medrano, 1990, Beckwitt et al., 2002, Hattori et al., 2003, Martinelli et al., 2010, Morin et al., 2005, Okuyama et al., 2014, Ortega et al., 2004, Fernando & Melnick, 2001, Vidya et al.,, 2003
ZFX	ZFX0582F ZFX0923R	ATAGGTCTGCAGACTCTTCTA	AGAATATGGCGACTTAGAACG	51	ODONTOCETES *(Odontoceti)**	Berube & Palsboll, 1996, Rosel et al., 2003
ZFY	ZFX0582F ZFY00767R	ATAGGTCTGCAGACTCTTCTA	TTTGTGTGAACTGAAATTACA	51	ODONTOCETES *(Odontoceti)**	Berube & Palsboll, 1996, Rosel et al., 2003
ZFX	ZFX0582F ZFX0785R	ATAGGTCTGCAGACTCTTCTA	CACTTATGGGGGTAGTCCTTT	51	MYSTICETES (*Mysticeti*)*	Berube & Palsboll, 1996, Rosel et al., 2003
ZFY	ZFX0582F ZFY0752R	ATAGGTCTGCAGACTCTTCTA	ATTACATGTCGTTTCAAATCA	51	MYSTICETES (*Mysticeti*)*	Berube & Palsboll, 1996, Rosel et al., 2003
ZFX		AAAGAAATCCCTCAAACACGT-TAC	TCGCCACCCRCAAATAG	50, 69-59	Bears (*Ursidae*)*	Bidon et al., 2013

Scaffold 318.2		AAGAAAAGTCATGCAACAGA-TACAG	TGATGCTTTGTGATCCTAATGTG	50, 69-59	Bears (*Ursidae*)*	Bidon et al., 2013
SMCY		GTCTTCCTCCTTAGAGGGTAA-TTAGG	TTCGTTTGATAATGGCCTAAAAC-TG	50, 69-59	Bears (*Ursidae*)*	Bidon et al., 2013
SRY	CSRY1F/R	GGGAAAGGCTCCTCACTATTT	AGGGATACATCCTCTCCTCTAC	62	*Sus scrofa domesticus*	Blanes et al., 2016
ZFX		GTGCTGCTTTGTCTTGGAATG	GAGGGAGTTAGGTCTGGATACT	62	*Sus scrofa domesticus*	Blanes et al., 2016
AMELX/Y		AAATTCTCTCACAGTCCAAG	CAACAGGTAATTTTCCTTTAG	53, 54	*Bos taurus*	Chen et al., 1999
Kdm5c/d		CTGAAGCTTTTGGCTTTGAG	CCACTGCCAAATTCTTTGG	54	*Mus musculus*	Clapcote & Roder, 2005
SRY	CSRY1F/R	CGCAAGGTGGCTCTAGAG	GCCTTSCGACGAGGTCGGTA	various	*Carivora**	DeCandia et al., 2016
ZFX	ZFX1F/R	TGGCCCAGATGGACATCC	AATGCTTTCCGGACTCATCG	various	*Carivora**	DeCandia et al., 2016
ZFX/Y	ZFY0097F, ZFY1204R	CATCCTTTGACTGTCTATCCTTG	CATTATGTGCTGGTTCTTTTCTG	55	*Ailuropoda melanoleuca*	Durnin et al., 2007
ZFX	(PS1)ZFX/Y_14, ZFX_175	CATCCTGAACACCTTACCAAG-AAGA	AGCATGAGAGAAATGCTTCCCAC	55	*Ailuropoda melanoleuca*	Durnin et al., 2007
ZFY	(PS1)ZFX/Y_14, ZFY_211	CATCCTGAACACCTTACCAAG-AAGA	CTTGTGCATTTTGTTGGCTCCTTTT	55	*Ailuropoda melanoleuca*	Durnin et al., 2007
ZFX	(PS2)ZFX/Y_75, ZFX_215	GAAGATAAGTTTACACAACCA	CTTGTGCATTTTGTTGGCTCC	55	*Ailuropoda melanoleuca*	Durnin et al., 2007
ZFY	(PS2)ZFX/Y_75, ZFY_167	GAAGATAAGTTTACACAACCA	CATTTTGTGAGTAAACAAAGCT	55	*Ailuropoda melanoleuca*	Durnin et al., 2007
AMELX/Y	SE47, SE48	CAGCCAAACCTCCCTCTGC		55	*Ailuropoda melanoleuca*	Durnin et al., 2007
AMELX	M4, M5	CAGCTTCCCAGTTTAAGCTCT	TCTCCTATACCACTTAGTCACT	NG	*Homo sapiens*	Faerman et al., 1995
AMELY	M4, M6	CAGCTTCCCAGTTTAAGCTCT	GCCCAAAGTTAGTAATTTTACCT	NG	*Homo sapiens*	Faerman et al., 1995
ZFX/Y		GGTGCAGCAACATGCTCTTA	TTAAAGCCTGAGGCGTCTGT	56	*Leporidae**	Fontanesi, Tazzoli, et al., 2008
AMELX/Y	Amel_2	GTTTAAGCCCTGATGGGTCA	CCGGGATAGAACTCTGGTCA	57	*Sus scrofa domesticus*	Fontanesi, Scotti, et al., 2008
AMELX/Y	Amel_3	GGGTGGATTCTTCATTCAGG	TCCAGGGGGTGTTCATTTTA	57	*Sus scrofa domesticus*	Fontanesi, Scotti, et al., 2008
AMELX/Y	AMEL2_F, AMEL2_R	CTCATCCTGGGCACCCTGSTT-ATATC	GGTACCACTTCARAGGGGTRAGC-AC	57	prosimians (*strepsirrhini*)*	Fredsted & Villessen, 2004

AMELX/Y	AMEL-fwd, AMEL-rvs	GTCTCTYYTAATGTKAACAAT-TGCAT	CCAACCATCAGAGCTTAAACTG	55, 65	*Homo sapiens*	Gibbon et al., 2009
AMELX/Y	AMEL-se AMEL-as	TGTKAACAATTGCATATTGAC-TTAATC	CACTATTCTTTACAGAGCCCAGG	56, 65	*Homo sapiens*	Gibbon et al., 2009
AMELX/Y	AMEL-fi, AMEL-ri	GGCACCCTGGTTATATCAACTT CA	CCATCAGAGCTTAAACTGGGAAG C	57, 65	*Homo sapiens*	Gibbon et al., 2009
DDX3X/Y	DDX31F, DDX3-1R	AGGAAGCCAGGAAAGTAA	CATCCA CGTTCTAAGTCTC	53	*Bos taurus*	Gokulakrishnan et al., 2012
DDX3X/Y	DDX3-2F, DDX3-2R	TGAGGAAGCCAGGAAAGTAA-GTAT	GCACCACCRTAWACCACACAA	53	*Bos taurus*	Gokulakrishnan et al., 2012
AMELX/Y		CCGCCCAGCAGYCCTTCCAG	TGGGGAATATYGGAGGCAGAGG	60	*Bos taurus*, *Ovis aries*, goat	Gokulakrishnan et al., 2013
ZFX/Y		AAATCAAAACCTTCATGCCAA	TTCCGGTTTTCAATTCCA	58	*Equus caballus*, *Bos taurus*	Han et al., 2010
SRY		TGCTATGTCCAGAGTATCCAA-CA	AATGGGAGGCCTGAAAGAGT	58	*Equus caballus*, *Bos taurus*	Han et al., 2010
ZFX/Y	KH1, KH2	TGACCAGCAAGGCAGAGAA	TCAACAACCCTTGTTCAGCT	45	*Enhydra lutris*	Hattori et al., 2003
ZFX	F-/R-ZFXBat	AGTCAAGGGRTGTCCATCR	GTTTGYASACCAGGTTCCTC	57	Bats (Chiroptera)*	Korstian et al., 2013
ZFY	F-/R-ZFYBat	GGTRAGDGCACAYRAGTTCC-ACA	TGCYATTACAAAACCTTTRTAGAT -AC	57	Bats (Chiroptera)	Korstian et al., 2013
ZFY	F-/R-ZFYFree	GGTGAGGGCACATGAGTTCC-ACA	TGCCATCACAATACCCTCTGTGAT AC	57	*Tadarida brasiliensis*	Korstian et al., 2013
AMELX/Y	RP-AmlF RP-AmlR	ACACTCCATGACTCCAACCC	CTGTATGGGGAACATCGGAG	54	*Ailurus fulgens*	Kumar et al., 2015
ZFX/Y	CerZFXYf CerZFXYr	GCTGACCCTGGAGAAGATGAC TTA	TCATTCTCAGGCTCACTCTCCACA	55	*Odocoileus virginianus*	Lindsay and Belant, 2007
SRY	CerSRYf CerSRYr	TGAACGAAGACGAAAGGTGG-CTCT	TACCCTATTGTGGCCCAGGTTTGT	55	*Odocoileus virginianus*	Lindsay and Belant, 2007
SRY	SRY-1F/R	CGAAGACGAAAGKTGGCTCT	TGTGCCTCCTCAAAGAATGG	60	*Capra hircus*	Malik et al., 2013
AMELX/Y	Amelogenin-F, Amelogenin-R	CAGCCAAACCTCCCTCTGC	CCCGCTTGGTCTTGTCTGTTGC	60	*Capra hircus*	Malik et al., 2013
ZFY	Zfx_R, Zfy_F	GACTAGACATGTCTTAACATC-TGTCC	CCTATTGCATGGACTGCAGCTTAT G	55	*Mus musculus*	McFarlane et al., 2013

Sly. Xlr	SX_F, SX_R	GATGATTTGAGTGGAAATGTG-AGGTA	CTTATGTTTATAGGCATGCACCAT-GTA	57	*Mus musculus*	McFarlane et al., 2013
Sry		TACAGCCTGAGGACATATTA	GCACTTTAACCCTTCGATGA	52	*Mus musculus*	Miyajima et al., 2009
Actb		AGCCATGTACGTAGCCATCC	GTGGTGGTGAAGCTGTAGC	52	*Mus musculus*	Miyajima et al., 2009
ZFX/Y	ZFY0097, P2–3EZ	CATCCTTTGACTGTCTATCCTTG	GCACTTCTTTGGTATCTGAGAAAG T	48	Cetacea*	Morin et al., 2005
ZFX/Y	CetZFXY_F1 CetZFXY_R1	AGTTTAAGTCGAGAGGTTTTT-TGAAA	TCTTGTTGGTAGTGTAATCACAGT-CAGT	56	Cetacea*	Morin et al., 2005
ZFX/Y	CETZFXprobe CETZFYprobe	AAAACCATCCTGAACACCTTA-CCAAGAA	AACCACCCTGAACACCTCACCAA	56	Cetacea*	Morin et al., 2005
AMELX/Y	AMXY-1F, AMXY-2	CTGATGGTTGGCCTCAAGCCT-GTG	TAAAGAGATTCATTAACTTGACTG	55	*Mandrillus sphinx*	Morrill et al., 2008
AMELX/Y	AMXY-8, AMXY-4R	TGACCAGCTTGGTTCTA	CTTGCTCATATTATACTTGACAAA	55	*Macaca tonkeana*	Morrill et al., 2008
AMELX/Y	AmelDeg F, AmelDeg R	CCCTGSGCTCTSTAAAGAATW-GTG	RTCRGMRCTTAAACTGGGAAGCT G	58	*Saimiri boliviensis*, *Homo sapiens*	Morrill et al., 2008
ZFX	ZFX-Ipfw, ZFX-Iprv	AGCCGTACCAGTGCCAGTA	GCGGACCTATACTCGCAGTA	53	*Procyon lotor*	Okuyama et al., 2014
ZFY	ZFY-Iprv, ZFY-Ipfw	AGCCGTACCAGTGCCAATT	TGCAGACCTATACTCGCAGAAT	53	*Procyon lotor*	Okuyama et al., 2014
ZFX/Y	ZFKF 203L, ZFKF 195H	CAAAAGGTGGCGATTCAATAA	ATGGAGAGCCACAAGCTRAC	58	Foxes and other canids*	Ortega et al., 2004
ZFX/ZFY	P1-5EZ, P2-3EZ	ATAATCACATGGAGAGCCAC-AAGCT	GCACTTCTTTGGTATCTGAGAAAG T		*Bubalus bubalis*	Pande & Totey, 1998
AMELX/Y	SE47, SE48	AGCCAAACCTCCCTCTGC	CCCGCTTGGTCTTGTCTGTTGC	56	*Ovis aries*, *Cervus elaphus*	Pfeiffer & Brenig, 2005
ZFX/Y		AAGTTTACACAACCACCTGG	CACAGAATTTACACTTGTGCA	56	*Felidae* *	Pilgrim et al., 2005
AMELX/Y		CGAGGTAATTTTTCTGTTTACT	GAAACTGAGTCAGAGAGGC	51	*Felis silvestris*	Pilgrim et al., 2005
SRY	PMSRYF, TtSRYR	CATTGTGTGGTCTCGTGATC	ACCGGCTTTCCATTCGTGAACG	51	Odontocets (*Odontoceti*)*	Rosel et al., 2003)
G6DP	Ex2, Ex2R	ACACACATATTCATCAT	ATGATGAATATGTGTGT	60	*Trichosurus vulpecula*, *Pseudocheirus peregrinus*	Russell et al., 2011

G6DP	Ex5, Ex5R	CAGGCCAACCGCCTCTTCTAC-CTGGCCTTG	CAAGGCCAGGTAGAAGAGGCGGT-TGGCCTG	60	*Trichosurus vulpecula*, *Pseudocheirus peregrinus*	Russell et al., 2011
G6DP	Ex13, Ex13R	TTCCAGTATGAGGGCACCTAC-AAGTGG	CCACTTGTAGGTGCCCTCATACTG-GAA	60	*Trichosurus vulpecula*, *Pseudocheirus peregrinus*	Russell et al., 2011
SRY		GCTATGTATGGCTTCTTGAATG	GTTAAACCTGAAACGAATGACAG	60	*Trichosurus vulpecula*, *Pseudocheirus peregrinus*	Russell et al., 2011
DDX3Y (ext.)	DBY-Fext, DBY-R	GCAAATTTGGTTTGTAGTCACA	CCATCTCAACATCGCTGAAC	55	*Canis lupus*, *Canis familiaris*	Sastre et al., 2009
ChrX (ext.)	AHTx40-F, AHTx40-Rex	GTAGCCCCATTTGTTTATATT-TGC	AAAACTGGACAGCCACATGC	55	*Canis lupus*, *Canis familiaris*	Sastre et al., 2009
DDX3Y (int.)	DBY-F (flourescent)	TTGGGGGTGGTTTTATTGTC	CCATCTCAACATCGCTGAAC	55	*Canis lupus*, *Canis familiaris*	Sastre et al., 2009
ChrX (int.)	AHTx40-R (Flourescent)	GTAGCCCCATTTGTTTATATT-TGC	GATCACTGTCTTACACCACAGGC	55	*Canis lupus*, *Canis familiaris*	Sastre et al., 2009
SRY	SRY-F, SRY-R	GATCAGTGGCAAACAGGAGAA	AGTTTCTGTGCCTCCTGGAA	57	*Callithrix jacchus*	Takabayashi & Katoh, 2011
ZFX/Y	Zf-F, Zf-R	CTGTGCATAACTTTGTTCC	TCATAAGGTAGTCCTCACA	57	*Callithrix jacchus*	Takabayashi & Katoh, 2011
AMELX/Y	gAML-XY-U1	AGCAACAGACAAGACCAAGC		54, 55	*Capra hircus*	Tsai et al., 2011
AMELX	gAML-X-D2		ACCCACCATAAAAGCTATTG	54, 55	*Capra hircus*	Tsai et al., 2011
AMELY	gAML-Y-D1		TGCCATATAGATAGACAAGC	54, 55	*Capra hircus*	Tsai et al., 2011
ALT1 marker	X1 and X3 (multiplex)	CCCTGATGAAGAACTTCTATCTC	GAAATTACACACATAGGTGGCACT	55	*Homo sapiens*	Tungwiwat et al., 2003
SRY	Y1.5, Y1.6	CTAGACCGCAGAGGCGCCCAT	TAGTACCCACGCCTGCTCCGG	55	*Homo sapiens*	Tungwiwat et al., 2003
ALT1 marker	X2, X3	TCGCCTTTCTCAAATTCCAAG	GAAATTACACACATAGGTGGCACT	55	*Homo sapiens*	Tungwiwat et al., 2003
SRY	Y1.7 AND Y1.8 (nested)	CATCCAGAGCGTCCCTGGCTT	CTTTCCACAGCCACATTTGTC	55	*Homo sapiens*	Tungwiwat et al., 2003
DDX3X/Y	F-Deadbox1, R-Deadbox1	TGATGTTTAGTGCTACTTTTCC-TAAGGAA	AGAGGTAGAGCCWACTCTKCCTA-CA	57 (apes), 62	Apes, monkeys*	Villesen & Fredsted, 2006

				(monk-eys)		
ZFX/Y	Rik8, Jim9	ATTCCAGGCAGTACCAAACAG	CCATCAGGGCCAATAATTATTG	62	Primates (*Primates*)*	Wilson & Erlandsson, 1998
AMELX/Y	DAME-F, DAME-R	GGCACCCTGGTTATATCAACT	CCACTTCYTCCYGCTTGGTCTT	55	*Canis familiaris*	Yan et al., 2013
AMELX/Y	DSI-F, DSI-R	ATAATGACAAAGAAAACATG-AC	CTGCTGAGCTGGCACCAT	55	*Canis familiaris*	Yan et al., 2013
DDX3X, DDX3Y	DDX3X/Y-Mam-F, DDX3X/Y-Mam-R	CAGATCTATGAGGAAGCCAGAAA	TCATACCGCTCTAGAGTTCGC	56	*Rhinolophus hipposideros*	Zarzoso et al., 2018
SRY		ATTCTTCGAGGAGGCACA	GGGTTCTGAGGAGGAGTTT	55	*Paguma larvata*	Zhang et al., 2016
ZFX		TTGGCGGTCCACAGCAAGAAC	CTGGCACTGGTATGGCTTCTC	55	*Paguma larvata*	Zhang et al., 2016

*For more information see table 1.
