# Supplementary material for: Molecular sexing assays in 114 mammalian species: In silico sequence reanalysis and a unified graphical visualization of diagnostic tests
Source: Ecol Evol. 2019 Apr 8;9(8):5018–28. doi: 10.1002/ece3.5093 (PMC6476764; doi:10.1002/ece3.5093)
Supplement: Supplementary file 2 [file ECE3-9-5018-s002.pdf]

Molecular sexing assays in 114 mammalian species: *in silico* sequence reanalysis and a unified graphical visualization of diagnostic tests

Supplementary file 2: Visualizations of molecular sexing methods

Rebeka Strah and Tanja Kunej

Department of Animal Science, Biotechnical Faculty, University of Ljubljana, Groblje 3, SI-1230 Domzale, Slovenia

Human (*Homo sapiens*). *AMELX, AMELY*

Faerman et al., 1995

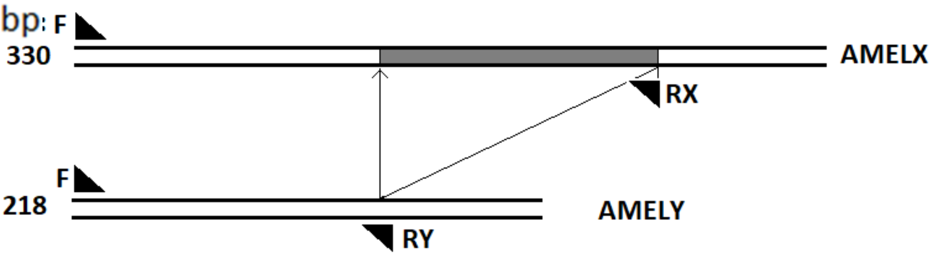


```
F:5′-CAGCTTCCCAGTTTAAGCTCT-3′
R:5′-TCTCCTATACCACTTAGTCACT-3′
R:5′-GCCCAAAGTTAGTAATTTTACCT-3′
```

```
AMELX    TACAGTTCCTACCACCAGCTTCCCAGTTTAAGCTCTGATGGTTGGCCTCAAGCCTGTGTC
AMELY    TACAGTTCCTACCATCAGCTTCCCAGTTTAAGCTCTGATGGTTGGCCTCAAGCCTGTGTT

AMELX    GTCCCAGCAGCCTCCCGCCTGGCCACTCTGACTCAGTCTGTCCTCCTAAATATGGCCGTA
AMELY    GCTCCAGCACCCTCCTGCCTGACCATTCGGATTGACTCTTTCCTCCTAAATATGGCTGTA

AMELX    AGCTTACCCATC-ATGAACCACTACTCAGGGAGGCTCCATGATAGGGCAAAAAGTAAACT
AMELY    AGTTTATTCATTCATGAACCACTGCTCAGGAAGGTTCCATGAAAGGGCAAAAAGTCAACT

AMELX    CTGACCAGCTTG----GTTCTAACCCAGCTAGTAAAATGTAAGGATTAGGTAAGATGTTA
AMELY    CTGACTGACCAGCTTGGTTCTATCCCATCCGGTAAAATGTAAAGAT--------------
                                                       **************

AMELX    TTTAAAACTCTTTCCAGCTCAAAAAACTCCTGATTCTAAGATAGTCACACTCTATGTGTG
AMELY    ------------------------------------------------------------
         ************************************************************

AMELX    TCTCTTGCTTGCCTCTGCTGAAATATTAGTGACTAAGTGGTATAGGAGAGACTCCGCAGA
AMELY    ------------------------------------------------------------
         ************************************************************

AMELX    ACAGCGGAATGCATGAGTTTTGGACGTCGGGTTTGAGGTTCTCCTCAACCTCTTACTAAC
AMELY    ---------------------------------------------------TAGGTAAAA
         ***************************************************

AMELX    TTTGTGATTTTGGCAAATCATTTCCTCTTTCTGGAACCCTGGTTTCCTCATCTGGAGAA
AMELY    TTACTAACTTTGGGCAAATAATTTCCTCTCTTTGGAACCCTGGTTTTCTCATTTGGACAA
```

Common primer | X-specific primer | Y-specific primer | SSIndel ******

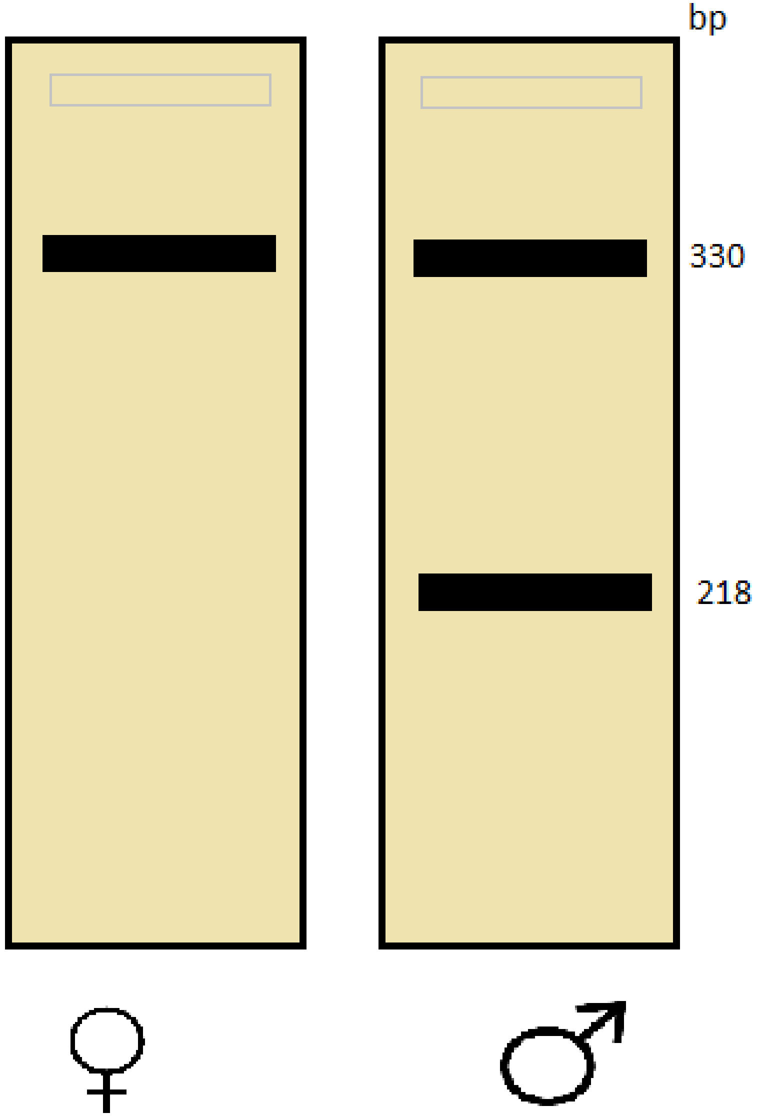

ENSG00000125363, ENSG00000099721

Human (*Homo sapiens*). *ATL1* marker, *SRY*

Tungwiwat et al., 2003

Multiplex PCR *ATL1*
F:5′-CCCTGATGAAGAACTTCTATCTC-3′
R:5′-GAAATTACACACATAGGTGGCACT-3′
Multiplex PCR *SRY*
F:5′-CTAGACCGCAGAGGCGCCCAT-3′
R:5′-TAGTACCCACGCCTGCTCCGG-3′
Nested PCR *ATL1*
F:5′-TCGCCTTTCTCAAATTCCAAG-3′
R:5′-GAAATTACACACATAGGTGGCACT-3′
Nested PCR *SRY*
F:5′-CATCCAGAGCGTCCCTGGCTT-3′
R:5′-CTTTCCACAGCCACATTTGTC-3′

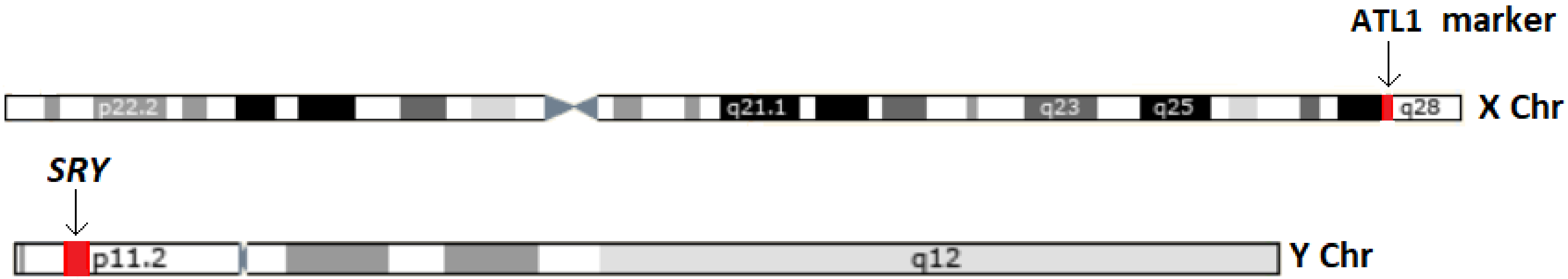

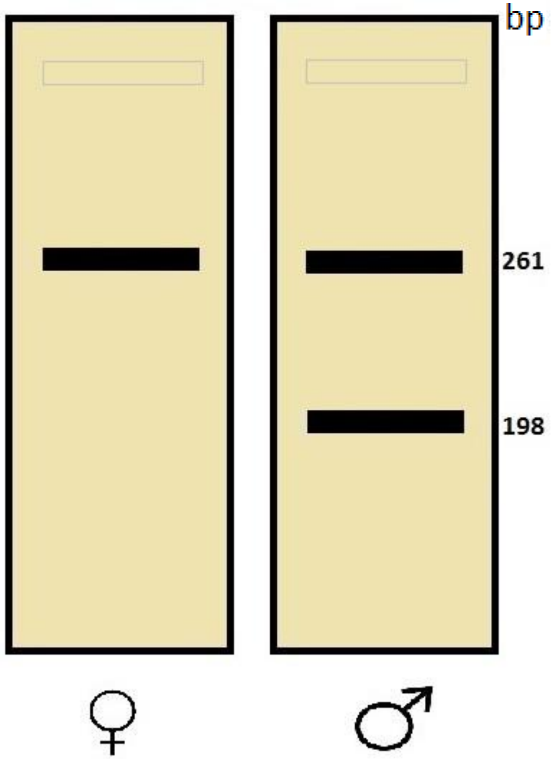

Human (*Homo sapiens*). *AMELX, AMELY.* System 2:

Gibbon et al., 2009

```
F:5'-GTCTCTYYTAATGTKAACAATTGCAT-3'
R:5'-CCAACCATCAGAGCTTAAACTG-3'
Inner primers:
F:5'-GGCACCCTGGTTATATCAACTTCA-3'
R:5'-CCATCAGAGCTTAAACTGGGAAGC-3'
```

```
AMELX 867   TAAAAACAGACCTCAAGTATATTCTGCACTATATAGATTTTTTAAAGTAGCTTCAGTCT    926
AMELY 352   GAAAAACAGACCTCAAATATATTCTGTACTATATAGATTTTTTAAAAGTAATTTTAGTCT   411

AMELX 927   CCTTTAATGTGAACAATTGCATACTGACTTAATCTCTTCCTCTCTTCTCTTCCTTCAC     986
AMELY 412   CTCTTAATGTTAACAATTGCATATTGACTTAATCTCTTACTCTCTCTTCCCTTCCTTCAC   471

AMELX 987   TCTCTCCCTTCCTCTCTCTTTCTATTCTCCTCCCCTCCTCCCTGTAAAAGCTACCACCTC   1046
AMELY 472   ACTCTCCCTTCCTCTCTCTTTCTCTTCTCCTCCCCTCCTCCCTATAAAAGCTACCACCTC   531

AMELX 1047  ATCCTGGGCACCCTGGTTATATCAACTTCAGCTATGAGGTAATTTTCTCTTTACTAATT    1106
AMELY 532   ATCCTGGGCACCCTGGTTATATCAACTTCAGCTATGAGGTAATTTTCTCTTTACTAATT    591

AMELX 1107  TTGACCATTGTTTGCGTTAACAATGCCCTGGGCTCTGTAAAGAATAGTGTGTTGATTCTT   1166
AMELY 592   TTGATCACTGTTTGCATTAGCAGTCCCCTGGGCTCTGTAAAGAATAGTGGGTGGATTCTT   651

AMELX 1167  TATCCCAGAT------GTTTCTCAAGTGGTCCTGATTTACAGTTCCTACCACCAGCTTC    1220
AMELY 652   CATCCCAAATAAAGTGGTTTCTCAAGTGGTCCCAATTTACAGTTCCTACCATCAGCTTC    711
                      *******

AMELX 1221  CCAGTTTAAGCTCTGATGGTTGGCCTCAAGCCTGTGTCGTCCCAGCAGCCTCCCGCCTGG   1280
AMELY 712   CCAGTTTAAGCTCTGATGGTTGGCCTCAAGCCTGTGTTGCTCCAGCACCCTCCTGCCTGA   771
```

Primer set AMEL-fwd/-rvs Primer set AMEL-fi/-ri Primer overlap SSIndel******

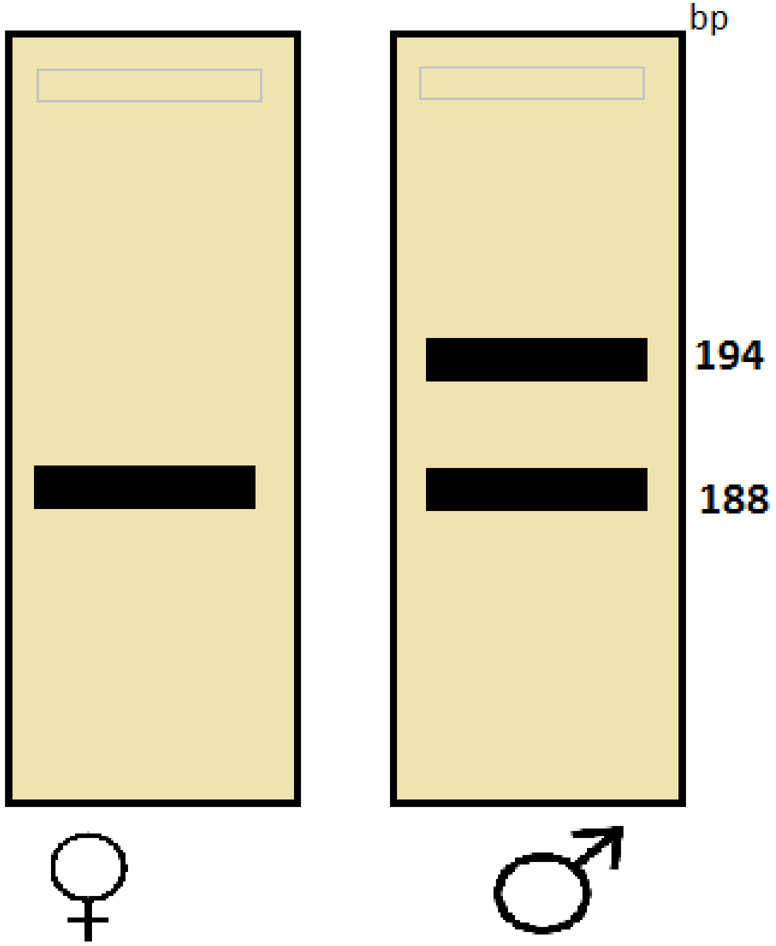

AMELX: ENSG00000125363 *AMELY*: ENSG00000099721

Human (*Homo sapiens*). ZFX, ZFY

Aasen and Medrano, 1990

```
ZFX  61   GAAGAAATACCGCTGTACTGACTGTGATTACACTACCAACAAGAAGATAAGTTTACACAA  120
          |||||| |||| ||||||||||||||||||||||||||| ||||||||||||||||| ||
ZFY  270  GAAGAAGTACCACTGTACTGACTGTGATTACACTACCAATAAGAAGATAAGTTTACATAA  329

ZFX  121  CCACCTGGAGAGCCACAAGCTGACCAGCAAGGCAGAGAAGGCCATTGAATGCGATGAGTG  180
          ||||||||||||||||||||||||||||||||||||||||||||||||||| ||||||||
ZFY  330  CCACCTGGAGAGCCACAAGCTGACCAGCAAGGCAGAGAAGGCCATTGAATGTGATGAGTG  389

ZFX  181  TGGGAAGCATTTCTCTCATGCAGGGGCTTTGTTTACTCACAAAATGGTGCATAAGGAAAA  240
          |||||||||||| |||||||||||||||||||||||||||||||||||||||||||||||
ZFY  390  TGGGAAGCATTTTTCTCATGCAGGGGCTTTGTTTACTCACAAAATGGTGCATAAGGAAAA  449

ZFX  241  AGGAGCCAACAAAATGCACAAGTGTAAATTCTGTGAATACGAGACAGCTGAACAAGGGTT  300
          ||| ||||||||||||||||||||||||||||||||||| |||||||||||||| |||||
ZFY  450  AGGGGCCAACAAAATGCACAAGTGTAAATTCTGTGAATATGAGACAGCTGAACAGGGGTT  509

ZFX  301  ATTGAATCGCCACCTCTTGGCAGTCCACAGCAAGAACTTTCCTCATATTTGTGTGGAGTG  360
          ||||||||||||||||||||||||||||||||||||||||||||||||||||||||||||
ZFY  510  ATTGAATCGCCACCTCTTGGCAGTCCACAGCAAGAACTTTCCTCATATTTGTGTGGAGTG  569

ZFX  361  TGGTAAGGGTTTTCGTCACCCGTCAGAGCTCAAAAAGCACATGAGAATCCATACTGGGGA  420
          |||||| ||||| || |||||||| || || | |||||||||| |||||||||| || ||
ZFY  570  TGGTAAAGGTTTCCGACACCCGTCGGAACTGAGAAAGCACATGCGAATCCATACCGGCGA  629

ZFX  421  GAAGCCGTACCAATGCCAGTACTGCGAATATAGGTCTGCAGACTCTTCTAACTTGAAAAC  480
          |||||| ||||||||||||||||| |||||||||||||||||||||||||||||||||||
ZFY  630  GAAGCCATACCAATGCCAGTACTGTGAATATAGGTCTGCAGACTCTTCTAACTTGAAAAC  689

ZFX  481  GCATGTCAAAACTAAGCATAGTAAAGAGATGCCATTCAAGTGTGACATTTGTCTTCTGAC  540
           ||| | ||||| |||||||||||||||||||||||||||||||||||||||||||||||
ZFY  690  ACATATAAAAACAAAGCATAGTAAAGAGATGCCATTCAAGTGTGACATTTGTCTTCTGAC  749

ZFX  541  TTTCTCGGATACCAAAGAGGTGCAGCAACATGCTCTTATCCACCAAGAAAGCAAAACACA  600
          |||||| ||||||||||| |||||||||||| ||||| ||||||||||||||||||||||
ZFY  750  TTTCTCAGATACCAAAGAAGTGCAGCAACATACTCTTGTCCACCAAGAAAGCAAAACACA  809
```

Primers | Mismatches to primers | *Hae*III recognition site | SNPs in Ensembl browser

EMBL acc. No.: NG_021253.1, NG_008113.1

F:5′-ATAATCACATGGAGAGCCACAAGCT-3′

R:5′-GCACTTCTTTGGTATCTGAGAAAGT-3′

*Hae*III recognition site: 5′...GG^CC...3′
3′...CC_GG...5′

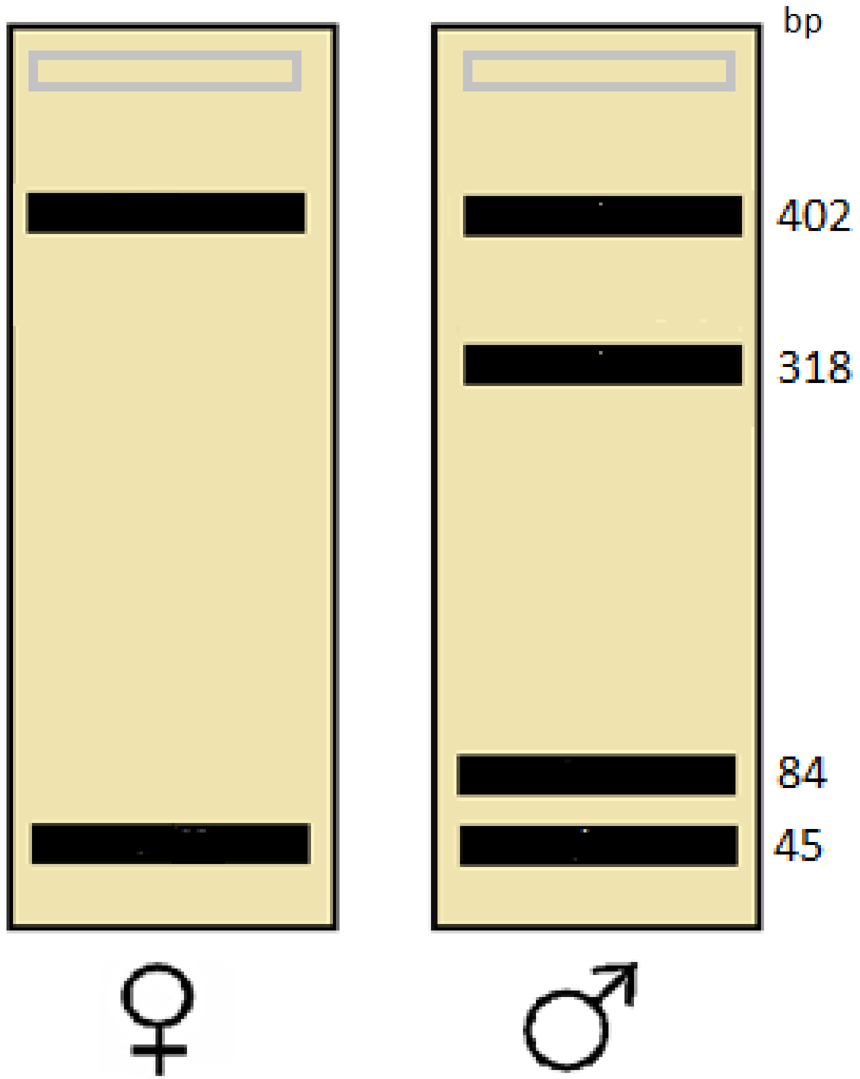

Wilson and Erlandson, 1998

Human (*Homo sapiens*), Chimpanzee (*Pan troglodytes*), Orangutan (*Pongo pygmaeus*), Gorilla (*Gorilla gorilla*), Gibbon (*Hylobates* sp.), baboon (*Papio*), marmoset (*Callithrix jacchus*). *ZFX,ZFY*

```
ZFX:     ATTCCAGGCAGTACCAAACAGGTGAGGGCGCACGAGTTCCATGGCGCAGCGTGCTCTGCG
ZFY:     ATTCCAGGCAGTACCAAACAGGTGAGTTCCACAGGGGTGTT-------------------

ZFX:     AGCTCTCAGAGGAAACTCTAGTATGTATCCACAGGGGTGTCACAATGGCATTTTAGCTGC
ZFY:     -----------------------------------------ATGATGGAGTTTTAGCTAG

ZFX:     TAGACCATATATAGCTTTGTCTATTGAACTTGAAAATATAATTTTCAGAATTCAGTGATA
ZFY:     TAGGCCACATGTATTTTTATGTGTTGAATTTGAAAGAAAAAATTTCAAAATTCAGTGATA

ZFX:     TTCATGAATGATTTCCTTGGATAAAAAGAAACAGGACATGGCTGAAACATGGATGAGAAA
ZFY:     TTCATGAATGGTTTTCTTGGATAAGAAGAAACAGTTGTGCATCAACCATTCAGGAAAAAG

ZFX:     AATTGAAAACTTGGATGATTTTTATGTGTACATGGAATGAAATCCCTCAAATATGTTATA
ZFY:     AATTAAAATCCTCTGG-TGATTTTAGTGAAAAGGAAAATAAATTTCTAAAATGTTACCTA

ZFX:     AGCATTAACTTTTTAAATAAATTAATACATTTAGAGAATCTATGTCAGCATAAAGCAGGC
ZFY:     ACTT--------TAAGTGAACGAAATTACATGGATCTACTTATACTAGCATAAAGCAGGT

ZFX:     ATAATTTACAGAGCAGCAGGATAAGTACTTTAGTTCATATGACATTC-------------
ZFY:     ATAATTTACCGAGAAGTGGAAGAAGTACCTAGGTTATTTGTAGGACTGATTACTATCCTA

ZFX:     -CTATTCTTGTATGATGATTTGCGTATTAAAAATTTT--ATGGGTATATTTAAACTTTGC
ZFY:     TGTTGTTTTGAATGCTGCTTTGCATATTAAAATTTATTTATAGGTGCAGTTAAGCTTTAC

ZFX:     TGTAAGTTAACGTAAGTTATGTTTTTTGGAATAAACAGTGGAAATTTTTGTCACCAAAGT
ZFY:     TGTTTGCATATATTTGGCTTGGAGTCAG----T----CACCAAAGCAGAAATGCTGGACT

ZFX:     TTATTTTTATGAATAATTTTCCACCAGTTTATTTACTTATTTTTTTTTGAGGCAGAATCT
ZFY:     TGATTTTTATGAGTTATTTGCTACACATTTCTAAATTCATGTTCTTTTGTCACTGCTTGA

ZFX:     TGC------TCTCTTGCCCAGGCTGGAGTGCAGTGGCGTGATCTCAACTCACACTGCAAC
ZFY:     TTGATTTTTTTTTTTTAACTGGAGGGGGTGAGATTGGTTCATAC---TTAACCAAACCAG

ZFX:     CTCTGCCCCCTGGGTACAGGTGGTTCTCCTGCCTCAACCTTCCAAGCGGCTAGGA-----
ZFY:     TTCTCTCGATTAGG---ACATTATTATAACTCTT-AACATTGAAAAGCAGTAAAAGGAAT

ZFX:     -TTACAGATGTGTGCCACTGTGCCCCACTAATTTTCATATTTTTAGTAGAGGCGGGTTTT
ZFY:     GTTAATAATTTAAA-AGTATTTGCCCACTAATGTTCAGAA--------CACA--AGCTTT

ZFX:     CAGCATGTTATGTTGGTCAGGGTGGTCTCGAATTCCTGAGTTCAGGTGATCTGCCTGCCT
ZFY:     AAAAAATTCATGAGGAGA---------------CCAGAAG--------------------

ZFX:     CGGCTTCCGAAAGTGCTGGGATTACAGGCGTGAGCCACCGTGCCCAGCCAATTTTCCACC
ZFY:     ------------------------------------------------------------

ZFX:     AATTTCTAAATTGCTGTAGGTTTAATCACTAATTGATAAAAGTTACATATTCATTAATTT
ZFY:     ------------------------------------------------------------

ZFX:     TATTAAACTAGAGGGAGTGAAGGCGGTACAGTCTTCATGAAGGAAGTTTCCAGACCATTT
ZFY:     ------------------------------------------------------------

ZFX:     TCTCTATGACTAGGTTGCTATATCTCTTGTGTTGAAAAACAGTAAAAGGAATGTTAAGCA
ZFY:     ------------------------------------------------------------

ZFX:     GCAAATAGTCCAATTTAGGAGTCCTTGGCCAACAGTGGTCAGAACCCACACTTTATATTC
ZFY:     ------------------------------------------------------------

ZFX:     GCAAAGAAACTGGAACAGAACTTGGTTTGAGCACTCATACTCCTTTCTTTTCCTTTTTTA
ZFY:     ---------------------TTTGATTAAGCACTCATACTGCTTTCTTTTCCTTTCTTA

ZFX:     GCAATAATTATTGGCCCTGATGGACATCCTTTGACTGTCTATCCTTGCATGATTTGTGGG
ZFY:     GCAATAATTATTGGCCCTGATGGTCATCCTTTGACTGTCTATCCTTGCATGATTTGTGGG
Primers SSIndel ******
```

```
ZFX:     ATTCCAGGCAGTACCAAACAGGTGAGGGCGCACGAGTTCCATGGCGCAGCGTGCTCTGCG
ZFY:     ATTCCAGGCAGTACCAAACAGGTGAGTT---------------------------------

ZFX:     AGCTCTCAGAGGAAACTCTAGTATGTATCCACAGGGGTGTCACAATGGCATTTTAGCTGC
ZFY:     ----------------------------CCACAGGGGTGTTATGATGGAGTTTTAGCTAG

ZFX:     TAGACCATATATAGCTTTGTCTATTGAACTTGAAAATATAATTTTCAGAATTCAGTGATA
ZFY:     TAGGCCACATGTATTTTTATGTGTTGAATTTGAAAGAAAAAATTTCAAAATTCAGTGATA

ZFX:     TTCATGAATGATTTCCTTGGATAAAAAGAAACAGGACATGGCTGAAAC-----ATGGATG
ZFY:     TTCATGAATGGTTTTCTTGGATAAGAAGAAACAGTTGT--GCATCAACCATTCAGGAAAA

ZFX:     AGAAAAATTGAAAACTTGGATGATTTTTATGTGTACATGGAATGAAATCCCTCAAATATG
ZFY:     AGAA--TTAAAATCCTCTGGTGATTTTA--GTGAAAAGGAAAATAAATT--TCTAAAATG

ZFX:     TTATAAACATTAACTTTTTAAATAAATTAATATATTTAGAG-AATCTATGTCAGCATAAA
ZFY:     TTACC-----TAACTTTAA--GTGAACGAAATTACATGGATCTACTTATACCAGCATAAA

ZFX:     GCAGGCATAATTTACAGAGCAGCAGGATAAGTACTTTAGTTCA----------TATGACA
ZFY:     GCAGGTATAATTTACAGAGAAGTGGAAGAAGTACCTAGGTTATTTGTAGGACTGATTACT

ZFX:     TTCCTATTC----TTGTATGATGATTTGCATATTAAAAATTT--TATGGGTATATTTAAA
ZFY:     ATCCTATGTTGTTTTGAATGCTGCTTCGCATATTAAAATTTATTTATAGGTGCAGTTAAG

ZFX:     CTTTGCTGTAAGTTAATGTAAGTTATGTTTTTTGGAATAAACCGTGGAAATTTTTGTCAC
ZFY:     CTTTACT-----------------------------------------------------

ZFX:     CAAAGTTTATTTTTATGAATAATTTTCCACCAGTTTATTTACTTATTTTTTTTTGAGGCAG
ZFY:     --------------------------------G----TTTGCATATAT-----TTGGCTT

ZFX:     AATCTTGCTCTCTCGCCCAGGCTGGAGTGCAGTGGCGTGATCTCAACTGACACTGCAACC
ZFY:     G----GAGTCAGTCACCAAAGCAGAAATGCTG----------------------------

ZFX:     TCTGCCCCCTGGGTACAAGTGGTTCTCCTGCCTCAGCCTTCCAAGCGGCTAGGATTACAG
ZFY:     ------------------------------------------------------------

ZFX:     ATGTGTGCCACTGTGCCCCACTAATTTTCATATTTTTAGTAGAGGCAGGTTTTCAGCATG
ZFY:     ------------------------------------------------------------

ZFX:     TTACGTTGGTCAGGGTGGTCTCGAACTCCTGAGTTCAGGTGATCTGCCTGCCTCGGCTTC
ZFY:     --------------------------------------------------GACTTGATTTT

ZFX:     TGAAAGTGCTGGGATTACAGGCGTGAGCCACCGTGCCCAGCCAATTTTCCACCAATTTCT
ZFY:     T-----------------ATGAGTT------------------ATTTGCTACACATTTCT

ZFX:     AAATTGCTGTAGGTTTAATCACTAATTGATAAAAGTTACATATTCATTAATTTTATTAAA
ZFY:     AAATTCATGT-TCTTTTGTCACTGCTTGATTG-------------ATTTTTTTTTTTTAA

ZFX:     CTAGAGGGAGTGAAGGTGATACAGTCTTCATGAATGAAGTTTCCAGACCATT-TTCTCTA
ZFY:     CTGGAGGGGGTGAGATTGGTTCATACTTA------------ACCAAACCAGTTCTCTCGA

ZFX:     TGACTAGGTTGCTATATCTCTTG-TGTTGAAAAACAGTAAAAGGAATGTTAAGTAGCAAA
ZFY:     TTAGGACATTATTATAACTCTTAACATTGAAAAGCAGTAAAAGGAATGTTAATAAT----

ZFX:     TAGTCCAATTTAGGAGTCCTTGGCCAACAGTGGTCAGAACCCACACTTTATATTCGCAAA
ZFY:     ---------TTAAAAGTATTTGCCCACTAATGTTCAGAACACAAGCTTTAAAAAATTCAT

ZFX:     GAAACTGGAACAGAACTTGGTTTGAGCACTCATACTCCTTTCTTTTCCTTTTTTAGCAAT
ZFY:     GA--GGAGACCACAAGTTTGATTAAGCACTCATACTGCTTTCTTTTCCTTTCTTAGCAAT

ZFX:     AATTATTGGCCCTGATGGACATCCTTTGACTGTCTATCCTTGCATGATTTGTGGGA
ZFY:     AATTATTGGCCCTGATGGTCATCCTTTGACTGTCTATCCTTGCATGATTTGTGGGA
Primers SSIndel ******
```

F:5′-ATTCCAGGCAGTACCAAACAG-3′

R:5′-CCATCAGGGCCAATAATTATTG-3′

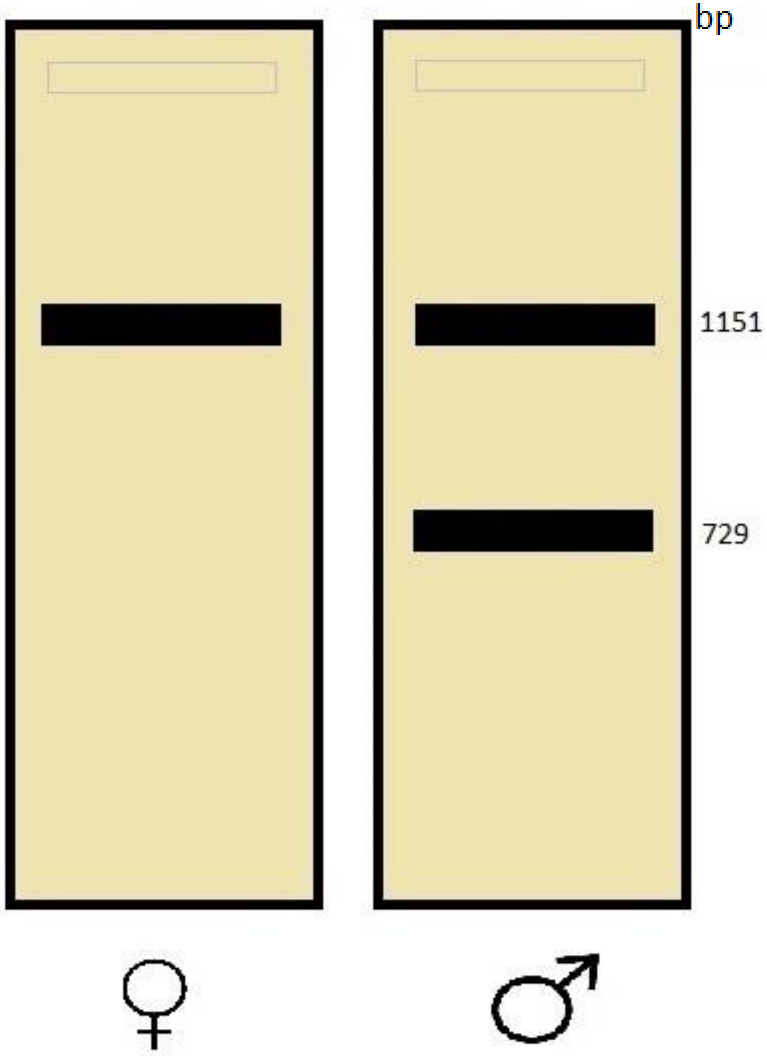

Human: ENSG00000005889, ENSG00000067646 Chimpanzee: ENSPTRG00000021747, ENSPTRG00000022471

Human (*Homo sapiens*), Chimpanzee (*Pan troglodytes*), Gorilla (*Gorilla gorilla*), Orangutan (*Pongo pygmaeus*), White-cheeked gibbon (*Nomascus leucogenys*), Rhesus macaque (*Macaca mulatta*), Hamadryas baboon (*Papio hamadryas*), Douc Langur (*Pygathrix nemaeus*), Black and white colobus monkey (*Colobus guereza*), Spider monkey (*Ateles fusciceps*), Bolivian squirrel monkey (*Saimiri boliviensis*), Brown capuchin (*Cebus paella*), Common marmoset (*Callithrix jacchus*). *DDX3X, DDX3Y*

Villesen and Fredsted, 2006

```
F:5'-TGATGTTTAGTGCTACTTTTCCTAAGGAA-3'
R:5'-AGAGGTAGAGCCWACTCTKCCTACA-3'
```

```
DDX3X  2    TACTTGGTGTTAGATGAAGCTGATCGGATGTTGGATATGGGGTTTGAGCCTCAGATTCGT  61
            ||||| ||||| |||||||||||| ||||| |||||||||| ||||| |||||||| |||
DDX3Y  2    TACTTAGTGTTGGATGAAGCTGATAGGATGCTGGATATGGGATTTGAACCTCAGATACGT  61

DDX3X  62   AGAATAGTCGAACAAGATACTATGCCTCCAAAGGGTGTCCGCCACACTATGATGTTTAGT  121
             | ||||| ||||||||||||||||| |||||||| || || ||||| ||||||||||||
DDX3Y  62   CGTATAGTTGAACAAGATACTATGCCACCAAAGGGCGTTCGTCACACCATGATGTTTAGT  121

DDX3X  122  GCTACTTTTCCTAAGGAAATACAGGTACTGTTTGATGTTGCAAATTTTATTTA-----TT  176
            ||||||||||||||||||||||||||||||||||| |||  || ||| ||| |     ||
DDX3Y  122  GCTACTTTTCCTAAGGAAATACAGGTACTGTTTGACGTTTGAACTTTCATTCAGAACATT  181
                                                                 ******

DDX3X  177  TA-GAAATTTGTTTATCT-CAGGTAAT--A---AT-AAAA-A--t--tt---t-tttt-C  218
            |  |    ||  |||||| ||| ||||  |   || |||| |  |  ||   | |||| |
DDX3Y  182  TGTGTTTATTCATTATCTTCAGATAATTTATGTATTAAAATAGGTAATTGAGTATTTTTC  241
              *                        ** ***  *      ** **  *** *    *

DDX3X  219  ------TTTCAGATGCTGGCTCGTGATTTCTTAGATGAATATATCTTCTTGGCTGTAGGA  272
                  ||||||||||| |||||||| || || |||||||||||||| |||||||||||
DDX3Y  242  TCACTGTTTCAGATGCTTGCTCGTGACTTTTTGGATGAATATATCTTTTTGGCTGTAGGC  301
            ******

DDX3X  273  AGAGTTGGCTCTACCTCTGAAAACATCACACAGAAAGTAGTTTGGGTGGAAGAATCAGAC  332
            ||||| |||||||||||||| |||||||||||||||||||||||||||||||| | |||
DDX3Y  302  AGAGTAGGCTCTACCTCTGAGAACATCACACAGAAAGTAGTTTGGGTGGAAGACTTAGAT  361

DDX3X  333  AAACGGTCATTTCTGCTTGACCTCCTAAATGCAACAG  369
            |||||||||||||| || ||| |  ||  ||||||||
DDX3Y  362  AAACGGTCATTTCTACTGGACATTTTAGGTGCAACAG  398
```

Primers Mismatch to primers SSIndel******

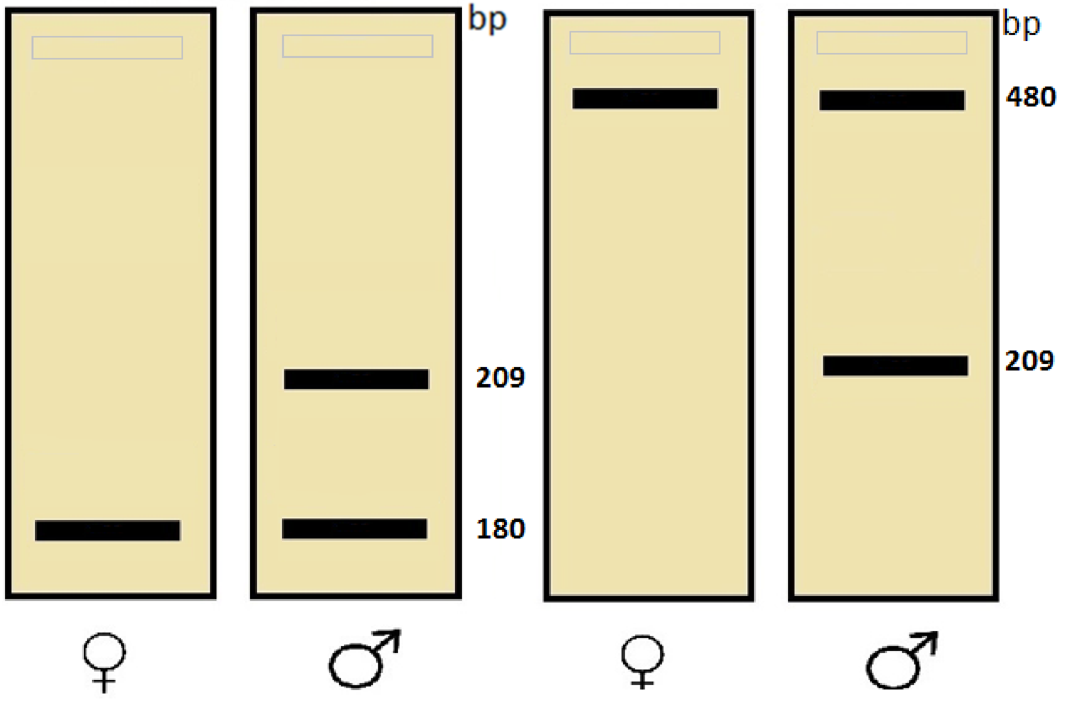

Macaca mulatta and Papio hamadryas

ENST00000360160.8, ENST00000399959.6

Macaca nemestrina, Macaca fuscata, Macaca mulatta, Macaca fascicularis. AMELX, AMELY

Morrill et al., 2008

```
AMELX      CTATCCCAGCTAGTAAAATGTAAGGATTAGGTAAGATGTTATTTAAGACTCTTTCCAGCT
AMELY      CTATCCCATCTGGTGAAATGTAAGGAT---------------------------------
                                      ************ ************* ***************

AMELX      CAAAAACTCCTGATTCTAAGATAGTCACACTCTATGTGTGTCTCTCATTTGGCTCTGCTG
AMELY      ------------------------------------------------------------
           ************ ************* ***************************** ************* ********

AMELX      AAATATTAGTGACTAAGTGGTATAGGAGAGACTCTGCAGAACAATGGAATGCATGAGTTT
AMELY      ------------------------------------------------------------
           ************ ************* ***************************** ************* ********

AMELX      TGGACATTGGGTTTGAGGTTCTCCTCAACCTCTTACTAACTGTGTGACTTTGGGCAAATC
AMELY      -------------------------------TAGGTAAAATTACTGACTTTGGGGGAAATA
           ************ ************* ***********

AMELX      ATTTCCTCTTTCTGGAACCCTGGTTTCCTCATGTGGAGAAAGGAAATAATTATAATAACC
AMELY      ATTTCCTCTCTTTGGAACCCCAGTTTCCTCATTTGGATGAGGGAAATTACTGTAATATTC

AMELX      ATATATCAAAATATTGTTTGGAGAATAATATACTTAATGAATATGAAA
AMELY      ACATTTCAAAATGTTGGACAGTAATATA----GTTAACAGTTACAAAA
                                       ****
Primers not shown SSIndel ******
```

F:5′-TGACCAGCTTGGTTCTA-3′

R:5′-CTTGCTCATATTATACTTGACAAA-3′

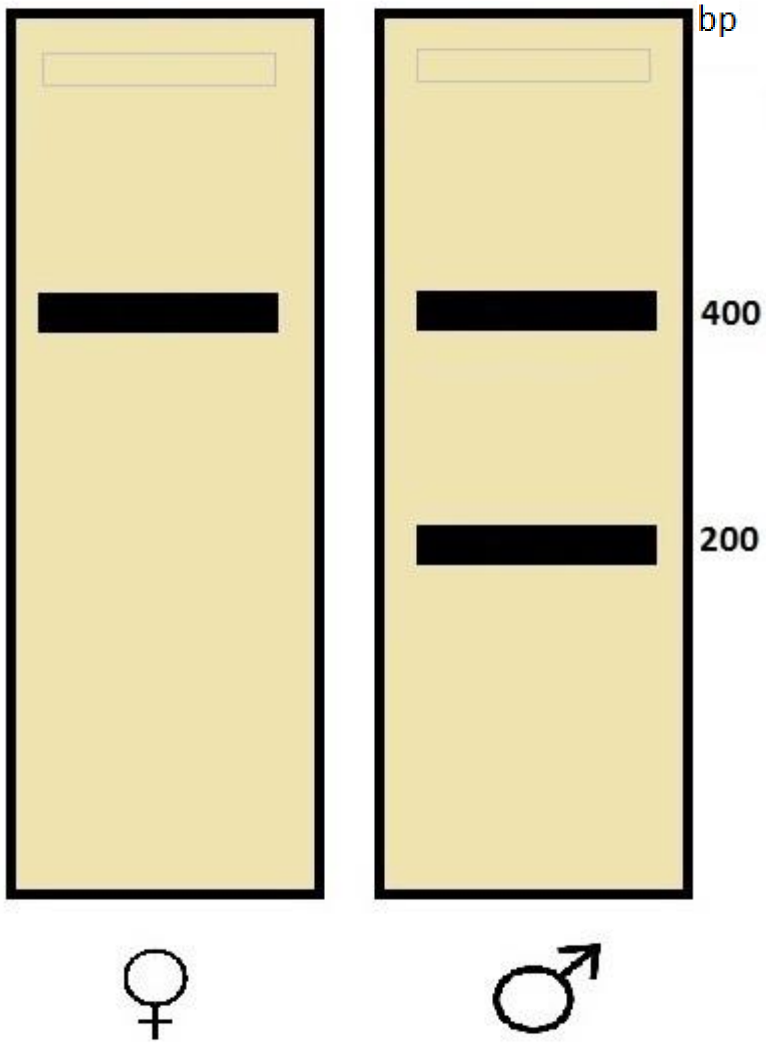

Macaca mulatta: EMBL acc. No.: EU748892, EU748893

Tonkean macaque (*Macaca tonkeana*). *ZFX, ZFY* — Fernando and Melnick, 2001

```
ZFX 199  GAAGATAAGTTTACACAACCACCTGGAGAGCCACAAGCTGACCAGCAAAGCAGAGAAGGC  258
ZFY 61   GAAGATAAGTTTACATAACCACCTGGAGAGCCACAAGCTGACCAGCAAGGCAGAGAAGGC  120

ZFX 259  CATAGAATGCGATGAGTGTGGGAAGCATTTCTCTCATGCAGGGGCTTTGTTTACTCACAA  318
ZFY 121  CATTCAATGTGATGAGTGTGGGAAGCATTTTTCTCATGCGGGGGCTTTGTTTACTCACAA  180

ZFX 319  AATGGTGCATAAGGAAAAAGGAGCCAACAAAATGCACAAGTGTAAATTCTGTGAATACGA  378
ZFY 181  AATGGTGCACAAGGAAAAAGGGGCCAACAAAATGCACAAGTGTAAATTCTGTGAATATGA  240

ZFX 379  GACAGCTGAACAAGGGTTACTGAATCGCCACCTCTTGGCGGTCCACAGCAAGAACTTTCC  438
ZFY 241  GACAGCTGAACAGGGGTTATTGAATCGCCACCTCTTGGCAGTCCACAGCAAGAACTTTCC  300

ZFX 439  TCATATTTGTGTGGAGTGTGGTAAGGGTTTTCGTCACCCCTCAGAGCTCAAAAAGCACAT  498
ZFY 301  TCATATTTGTGTGGAGTGTGGTAAAGGTTTCCGACACCCATCGGAACTCAAAAAGCACAT  360

ZFX 499  GAGAATCCATACTGGGGAGAAGCCGTACCAATGCCAGTACTGCGAATATAGGTCTGCAGA  558
ZFY 361  GCGAATCCATACAGGGGAGAAGCCATACCAATGCCAGTACTGCGAATATAGGTCTGCAGA  420

ZFX 559  CTCTTCTAACTTGAAAACGCATGTCAAAACTAAGCATAGTAAAGAGATGCCATTCAAGTG  618
ZFY 421  CTCTTCCAACTTGAAAACACATATAAAAACAAAGCATAGTAAAGAGATGCCATTCAAGTG  480

ZFX 619  TGACATTTGTCTTCTGACTTTCTCGGATACCAAAGAGGTGCAGCAACATGCTCTTATCCA  678
ZFY 481  TGATATTTGTCTTCTGACTTTCTCAGATACCAAAGAAGTGCAGCAGCATACTCTTGTCCA  540
```

F:5′-ATAATCACATGGAGAGCCACAAGCT-3′
R:5′-GCACTTCTTTGGTATCTGAGAAAGT-3′

HaeIII recognition site: 5′...GGCC...3′ / 3′...CCGG...5′

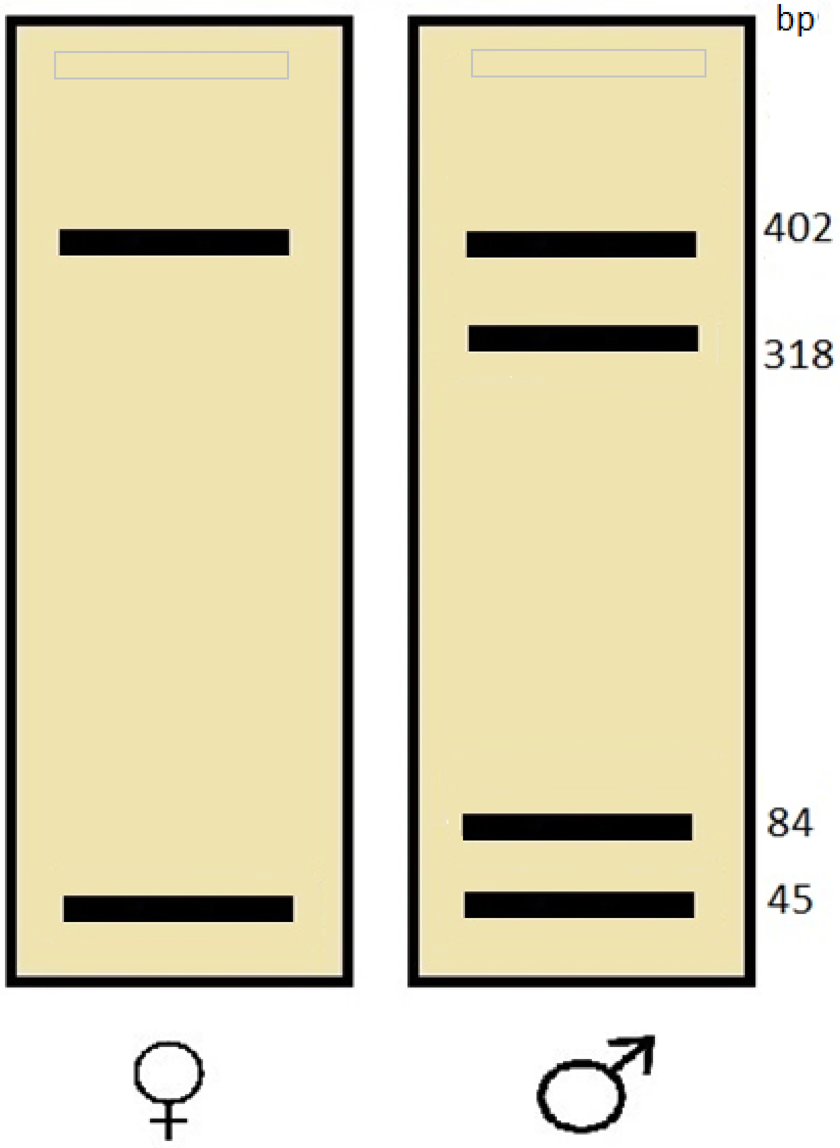

Primers | Mismatch to primers | *Hae*III recognition site | Restriction site ▲

Diagnostic site ******

EMBL acc. No.: HM071225.1, HM071747.1

Mandrill (*Mandrillus sphinx*). *AMELX, AMELY*

Morrill et al., 2008

```
AMELX     TTGGTTCTATCCCAGCTAGTAAAATGTAAGGATTAGGTAAGATGTTATTTAAGACTCTTT
AMELY     --------------------------------------------------------CCCT
          ********************************************************

AMELX     CCAGCTCAAAAACTCCTGATTCTAAGATAGTCACACT----CTATGTGTGTCTCTCATTT
AMELY     CCTGCCTGATTACTCGGATTGACTCTCTCCTCCTAATGGCTGTAAGCTTATTCATCATGA
                                               ****

AMELX     GGCTCTGCTGAAATATTAGTGACTAAGTGGTATAGGAGAGACTCTGCAGAACAATGGAAT
AMELY     ACCACTGCTCAGGAAGATTCCATGAA------------AGGGCAAAAAGTCAACTCTGAC
                                    ************

AMELX     GCATGAGTTTTGGACATTGGGTTTGAGGTTCTCCTCAACCTCTTACTAACTGTGTGACTT
AMELY     TGACCAGCTTGGTTCTATCCCATCTGGTGAAATGTAAAGATTAGGTAAAATTACTGACTT

AMELX     TGGGCAAATCATTTCCTCTTTCTGGAACCCTGGTTTCCTCATGTGGAGAAAGGAAATAAT
AMELY     TGGGGAAATAATTTCCTCTCTTTGGAACCCCAGTTTCCTCATTTGGATGAGGGAAATTAC

AMELX     TATAATAACCATATATCAAAATATTGTTTGGAGAATAATATACTTAATGAATATGAAAAG
AMELY     TGTAATATTCACATTTCAAAATGTTGGACAGTAATA----TA-GTTAACAGTTACAAAAC
                                              ****

AMELX     TACTTTGTCAAGTATAATATGAGCAAGTTTACTAATTTTTTTGTATCAATTAAATGTCAT
AMELY     TGCTTTGTCAGGTATAATATGAGCAAGGTAATTAATTTTTTA---TTGATTAAATGCTGT
                                                    ***

AMELX     ATTACTGTATGAAGAATCCTCAAACCTAAGTCTAACCAAGTATATATACTGTTCAGAAAG
AMELY     ACTACTAGAAAAAGAATCCCCAAACCTAAGGTT---AACCAATATATACTGTTCAGAAAG
                                           ***

AMELX     GAATAAAATTCTTACTTTTCTCACAGGTTCAGGTAACAATCTGTGAGTTTATTTACTTAT
AMELY     GAATAAAATTCTTACTTCTCTCACAGGTTCAGGTAACAATCTTTGAGTTTATTTACTTAC

AMELX     AAAAGCTGAAGACAAATGTTAATAAGAATTTGAGGCAAGATTTTCTGTTAAGCCTAAAAG
AMELY     ACAAGCTGCTGACAAATGTTAGTAAGAATCTGATGCAAGGTGTTCTGTTAAACCTAAAAG

AMELX     ATTGACACATCTGATGAGTAAATCTGTGTTTCCAGGATGAGGGACAGTGTTTGCACCTCT
AMELY     ACTGACAAATTTGATTAGTAAATCTGTGTTTTTAGGATGAGGGACAGTGTTTGCACCTTT

AMELX     CTTTTTCCCATTGTGACATCAAAGAAAAAAATGAAATTAACATCATGTCATATTATTATG
AMELY     TTTTCCCCAT-TGTGAC-ATCAAAGGAAAGATGAAATTAACATTATGTCACATTATTATG

AMELX     TCATAATTTTGTGTTTGTTTTGCTCTTACAATGAAAAGCAGGAATTACGGAATTAAACAG
AMELY     GCATAATTTTGTGTTTGCTTTGCTCTTACAATGAAGAGCAGGAGCTATGAAAATAAATAG

AMELX     ATTTACTCC
AMELY     ATTTACTCC
```

Primers not shown SSIndel ******

EMBL acc. no.: EU748887, EU748889

F: 5’-CTGATGGTTGGCCTCAAGCCTGTG-3’

R: 5’-TAAAGAGATTCATTAACTTGACTG-3’

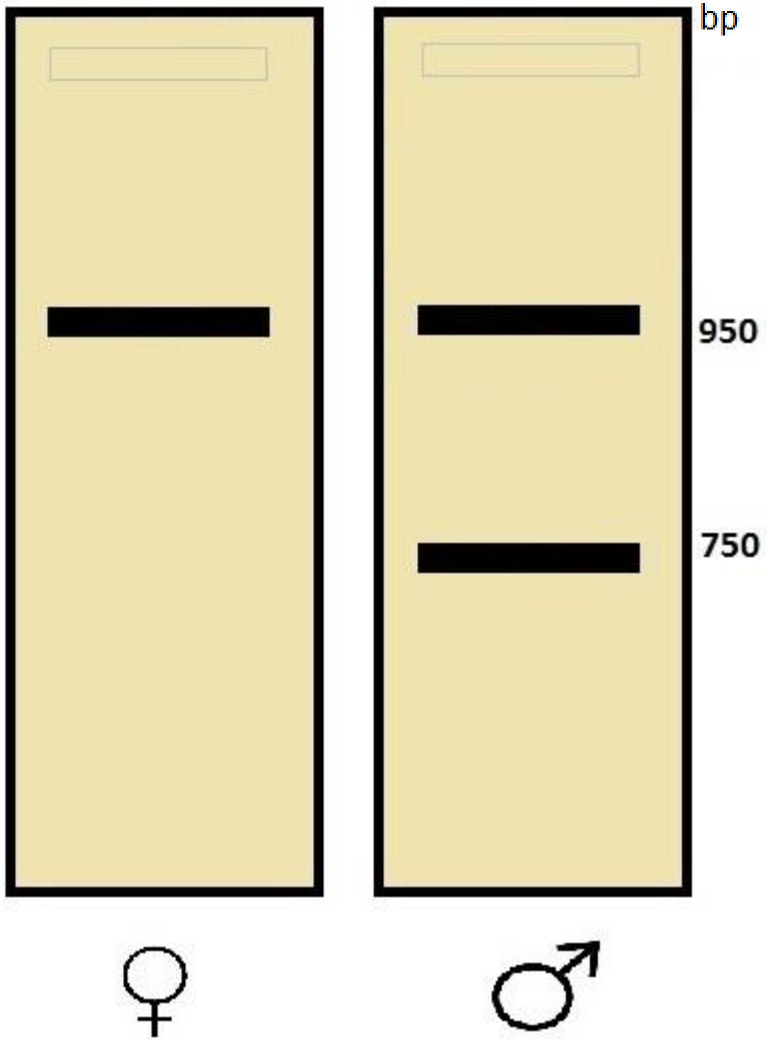

Approximate band sizes

Common marmoset (*Callithrix jacchus*). *ZFX, ZFY*

Takabayashi and Katoh, 2011

```
ZFX   1  TGATGACCCAGATTCTGTTGTAATCCAAGATGTTATTGAGGACGTTGTTATAGAAGATGT  60
ZFY   1  TGATGACCCAGACTCAGTTGTAATCCAAGATGTTATTGAAGATGTTGTCATAGAAGATGT  60

ZFX  61  TCAGTGCCCAGATATCATGGAAGAAGCAGATGTATCTGAAACGGTCATCATTCCTGAGCA  120
ZFY  61  TCAGTGTTCAGATATCTTAGAAGAAACAGATGTATCTGAAAATGTTATCATTCCTGAGCA  120

ZFX 121  AGTGCTGGACTCAGATGTAACTGAAGAAGTTTCTTTAGCACATTGCACCGTCCCAGATGA  180
ZFY 121  AGTGCTGGACTCAGATGTAACTGAAGAAGTTTCTGTGTCACACTGCACAGTCCCAGATGA  180

ZFX 181  TGTTTTAGCTTCTGACATTACTTCAGCCTCAATGTCTATGCCAGAACACGTCTTGACGAG  240
ZFY 181  TGTTTTAGCTTCTGACATTACTTCATCCTCAGTGTCTATGCCAGAACATGTTTTAACAAG  240

ZFX 241  TGAATCTATACATGTGTCTGATGTTGGACATGTTGGACATGTTGAACATGTGGTTCATGA  300
ZFY 241  TGAATCCATGCATGT-----ATGT-G-ACA--TTGGACATGTTGAACATGTGGTACGTGA  291

ZFX 301  TAGCGTAGTAGAAGCAGAAATTGTCACGGATCCTCTGACCACCGATGTAGTTTCAGAAGA  360
ZFY 292  TAATGTAGTGGAAGCAGAAATTATTACTGATCCTCTGACCAGTGACGTAGTTTCAGAAGA  351

ZFX 361  AGTATTGGTAGCAGACTGTGCCTCTGAAGCAGTCATAGATGCCAATGGATCCCTGTGGA  420
ZFY 352  AGTATTGATAGCAGACTGTGCCCCTGAAACGATCACAGATGC---TGGGATCTCAGTGGA  408

ZFX 421  CCAGCAAGATGATGACAAAGGCAAC  445
ZFY 409  CCAGCGAGATGATGACAAAGGCAAC  433
```

DdeI recognition site *MseI* recognition site Restriction site ▲ Diagnostic site ***

F: 5′-CTGTGCATAACTTTGTTCC-3′

R: 5′-TCATAAGGTAGTCCTCACA-3′

RE recognition site:

DdeI: 5′...C▼TNAG...3′ / 3′...GANT▲C...5′

MseI: 5′...T▼TAA...3′ / 3′...AAT▲T...5′

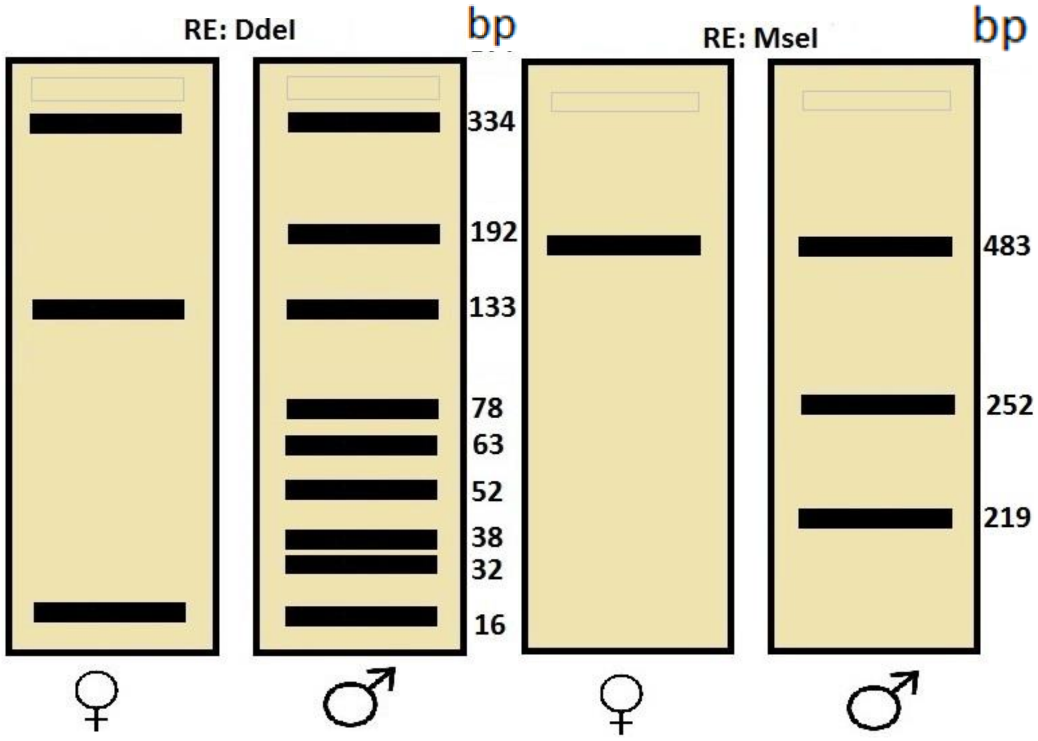

EMBL acc. No.: AB526243, AB526244

Prosimians: **gray mouse lemur (*Microcebus murinus*), Berthe's mouse lemur (*Microcebus berthae*), fat-tailed dwarf lemur (*Cheirogaleus medius*), Coquerel's dwarf lemur (*Mirza coquereli*), red-fronted brown lemur (*Eulemur fulvus rufus*), red-tailed sportive lemur (*Lepilemur ruficaudatus*), and ring-tailed lemur (*Lemur catta*) *AMELX, AMELY***

Fredsted and Villesen, 2004

```
AMELX     CTCTCTTTTTCTGTTCTCCTTCCCCATCCCTATAAAAGCTACCACCTCATCCCGGGCACC
AMELY     TCTCTCTTTTCTATTTTCCTTCCCCATCCCTATAAAAGCTACAACCTCATCCTGGGCACC
                ****** ** *************************** ********* *******

AMELX     CTGCTTATATCAACTTCAGCTATGAGGTAATTTTTCTTTTTACTAATTTTGACCATTGTT
AMELY     CTGGTTATATCAACTTCAGCTATGAGGTAATTTTTCTCTTTACTAATTTTGACCATTTTT
          *** ********************************* ******************* **
          [Non-diagnostic alignment is ommitted]

AMELX     GTTAACCAAGTATATACTGCTCAGAAAGGAATGAAATTCTTCCTT-----CTCTAACA--
AMELY     GTTAGACTTTAAAAGGTTGCTAAATATGGCCAACATTTATTTTTAGGAGATTTTACCATC
          ****   *    * *   **** *  * **     * ** **  *        * ** **

AMELX     ------GGTTCAGGTAATAATCTATGAGTCTGTTTACTTATACAAGCTGAAGAAAAATGT
AMELY     TTCTTTTGTGGTGTTAATGGTTGAAGTGAATATTAAGTTATATAATCTTTTG--------
                 **   * ****  *  * * *  * ** * ***** ** **   *

AMELX     TAGTAAGAATTTGAGGCAAGATTCTCTATTAAACCTTAAAAGGTTGACATATTTGAGCAG
AMELY     ------------------CGATTGTCCGTGTAATGAAG----------------------
          *****************                     **********************

AMELX     TAAATCTGTGTTTCTAGGATGAGGGACAGTGTTTCCACCTCCCCCCCCCCTTTTTTTTTA
AMELY     ------------------------------------------------------------
          ************************************************************

AMELX     CCATTGTAACATTAAATGAAAAAAGAATGAAATTACTATTATGTCACATTAATATGTCAT
AMELY     ------------------------------------------------------------
          ************************************************************

AMELX     AATTTTTGTTTGTTCTGCCCTTGCAGTGAAGAGCAGGAGCTATAGAATTAAACAGAATTA
AMELY     ------------------------------AGCAGGTGTCATGGAATTAAACAGAATTG
          ************************* *****

AMELX     CCATTTTTGCAACTTTAGTTAACTTAATGAACCTCTTTAACTCTCCATGACCTTACCTGC
AMELY     CTACTTCTGCATCCTTAGTTAGATTAATGAGCCTCTAACTTTCCAT--AGCCTTGT-CTA
                                                        **

AMELX     AAAAGCGAGGATGAGAATACCTGCCTTAGAGCATGTGAGAAAATGTGAAAAATGTGTGCA
AMELY     TAAAATGTACGTGATGATACTTCTCTCAGAGCATGTGAGACAATATAAAAAATGTGTGTG

AMELX     ATGGATGTAACACAGTGCCTGACACATAGAAAGCACCCCAAAAATGTTTACATTCTTCTT
AMELY     ATAGATGCAATGCAGTTCCTGATTCAGAAAGCACCC--AACAAATCTTTATGT---TCTT
                                              **                ***

AMELX     TCTTTTGTAGAACTCACATTCTCAGGCTATCAGTATTTACGGGACTGAATTAGTGAGTCT
AMELY     CTCTTCAGGAGACATACTCTTTTAGGCTATCACTATTGATAGAACTGCATTTGTGAGTCT

AMELX     ATATTTCATACTGCATTAGTGAGTTTCTATATCAGAATGAAGTAAATTAAATCAAATACA
AMELY     ACATTTCATACTGCATTAATGCATTTCTATATTTGATCAAAGTAAATTAAATAAAATGTA

AMELX     TTCTCATATCTCTTTCTTTTAAGGTGCTTACCCCTTTGAAGTGGTACCAGAGCATGATAA
AMELY     TTCTCATATC----TCTGTTAAGGTGCTTACGCCTTTGAAGTGGTACCAAAGCATGATAA

Primers Mismatch to primers SSIndel******
```

EMBL acc. no.: AB091785.1, AB091786.1

F:5′-CTCATCCTGGGCACCCTGSTTATATC-3′

R:5′-GGTACCACTTCARAGGGGTRAGCAC-3′

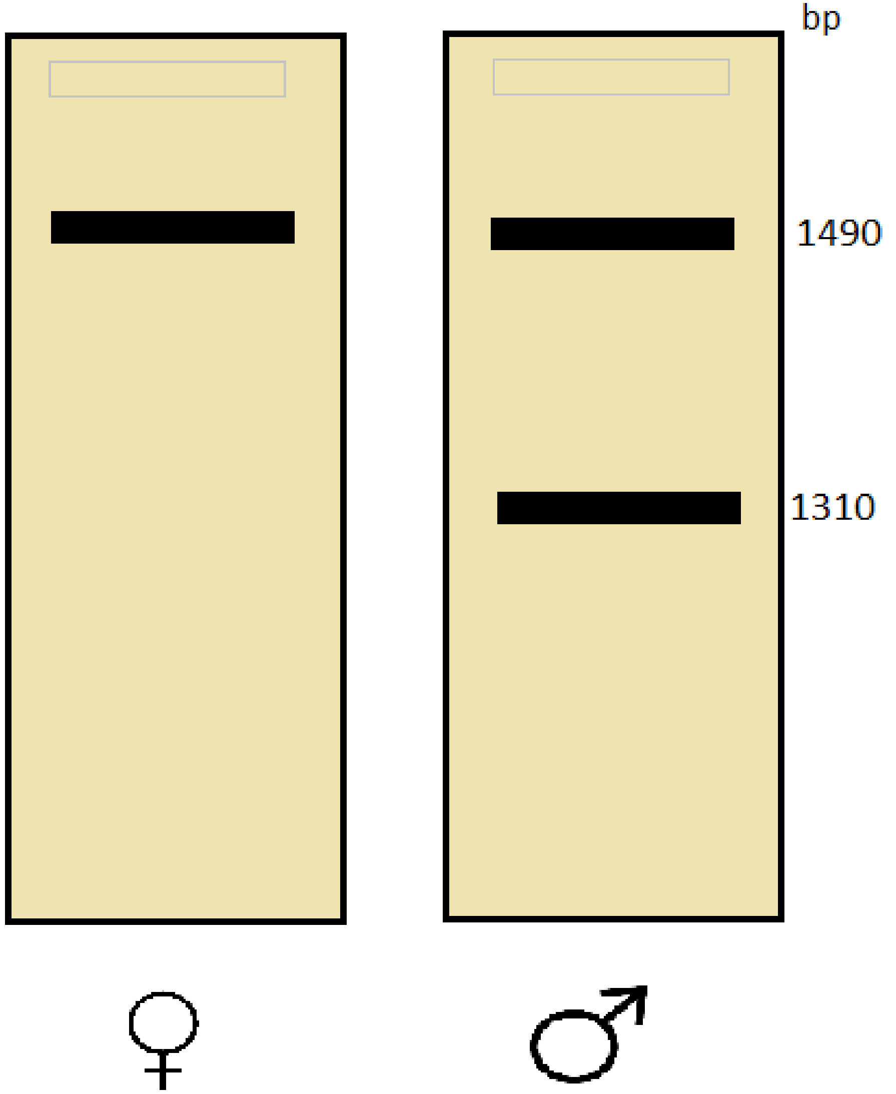

Mouse (*Mus musculus*). *Xlr, Sly*

McFarlane et al., 2013

SX

F: 5´-GATGATTTGAGTGGAAATGTGAGGTA-3´

R: 5´-CTTATGTTTATAGGCATGCACCATGTA-3´

Zfy

F: 5´-GACTAGACATGTCTTAACATCTGTCC-3´

R: 5´-CCTATTGCATGGACTGCAGCTTATG-3´

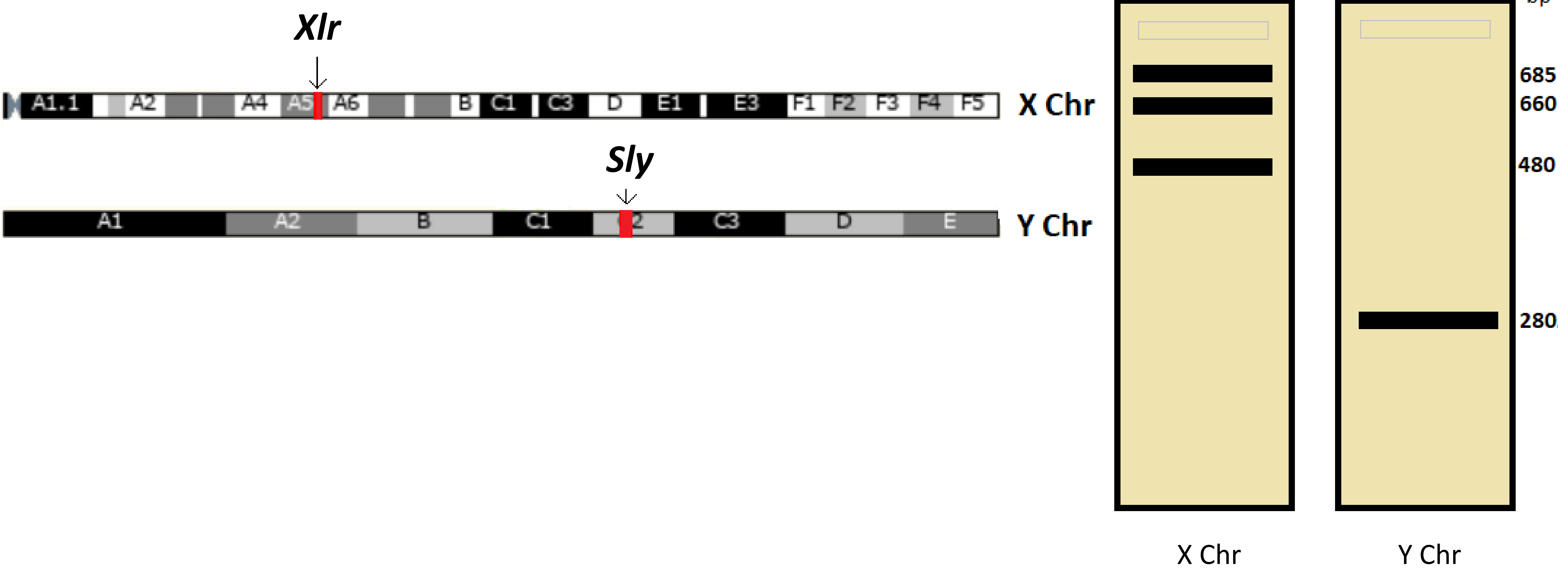

Mouse (*Mus musculus*). *Kdm5c, Kdm5d.* Synonyms: *Jarid1c, Jarid1d*

Clapcote and Roder, 2005

F:5′-CTGAAGCTTTTGGCTTTGAG-3′

R:5′-CCACTGCCAAATTCTTTGG-3′

```
Kdm5c 1     GAGTGTAAGCGGCCCCTGAAGCCTTTGGCTTTGAGCAAGCTACCCGGGAATATACTCTG  60
            ||||||||| | ||||||||||| |||||||||||||| ||||| | ||| |||||| ||
Kdm5d 1     GAGTGTAAGAGTCCCCTGAAGCTTTTGGCTTTGAGCAGGCTACACAGGAGTATACTTTG  60

Kdm5c 61    CAGAGCTTTGGAGAGATGGCTGACTCCTTTAAAGCTGACTACTTCAACATGCCTGTGCAT  120
            ||||| ||||| ||||||||||||||||| || |||||||||||||||||||||||||||
Kdm5d 61    CAGAGTTTTGGTGAGATGGCTGACTCCTTCAAGGCTGACTACTTCAACATGCCTGTGCAT  120

Kdm5c 121   GTAGGTGATGCAGGGCTGGGGTAGTGCTGAGACTAGGTTCATAGGCACTGGTACTGTACC  180
            ||| ||||       |||    | || | |   ||   || ||| |     |  ||
Kdm5d 121   GTATGTGA-------CTG----AATGATTAATGTA---TCCTAGTC-----TTATG----  157
                    *******    ****             ***        *****     ****

Kdm5c 181   TCCCTAAATTAATGTTGACAGGATAAGATGTC-ATTTACTATCCTGTTCCCCCAGATGGT  239
                 |||  ||   |  |||   |  ||| | || |  |  ||| ||| |  |||||||
Kdm5d 158   -----AAAGCAA--ATACCAGTGAACCATGGCTATCTTTTCACCTATTCTCATAGATGGT  210
            *****       **

Kdm5c 240   ACCCACAGAACTTGTAGAGAAGGAGTTTTGGCGGCTGGTGAATAGCATCGAGGAAGACGT  299
            ||| |||||| |||| || ||||| || ||| |||||||||  ||||| ||||| || ||
Kdm5d 211   ACCTACAGAAGTTGTGGAAAAGGAATTCTGGAGGCTGGTGAGCAGCATTGAGGAGGATGT  270

Kdm5c 300   AACTGTGGAGTATGGGGCTGACATCCATTCCAAAGAATTTGGCAGCGGTTTCCCTGTCA  358
             || || || ||||| || ||||| || ||||||||||||||||| || || |||||||
Kdm5d 271   GACAGTTGAATATGGAGCAGACATTCACTCCAAAGAATTTGGCAGTGGGTTTCCTGTCA  329
```

Primer | Mismatch to primer | SNP | SSIndel ******

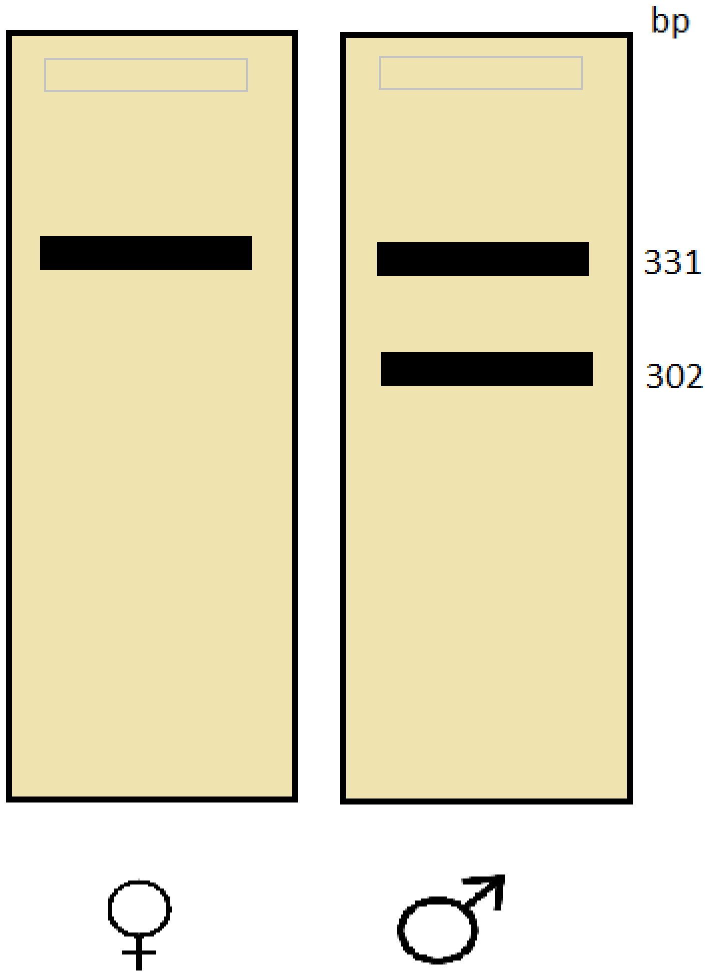

ENSMUST00000082177, ENSMUST00000055032

Brown rat (*Rattus norvegicus*). *Actb, Sry*

Miyajima et al., 2009

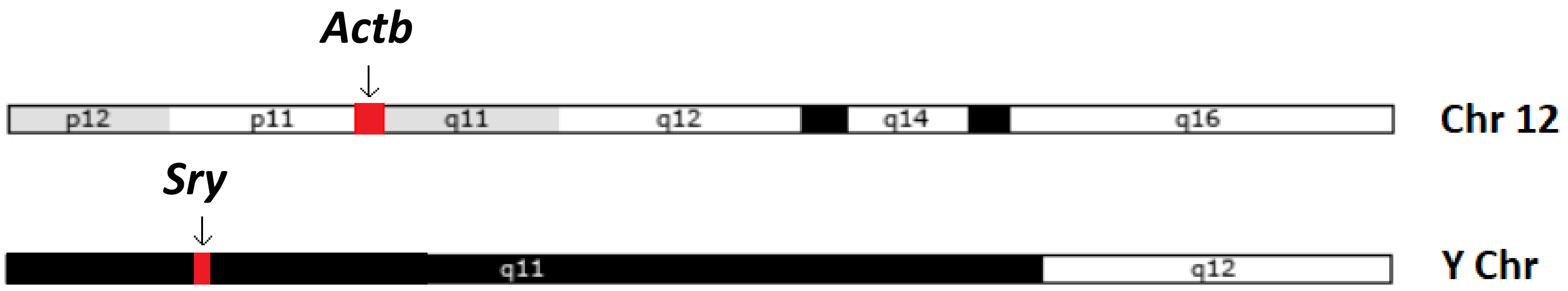

Sry

F:5′-TACAGCCTGAGGACATATTA-3′

R:5′-GCACTTTAACCCTTCGATGA-3′

Actb

F:5′-AGCCATGTACGTAGCCATCC-3′

R:5′-GTGGTGGTGAAGCTGTAGC-3′

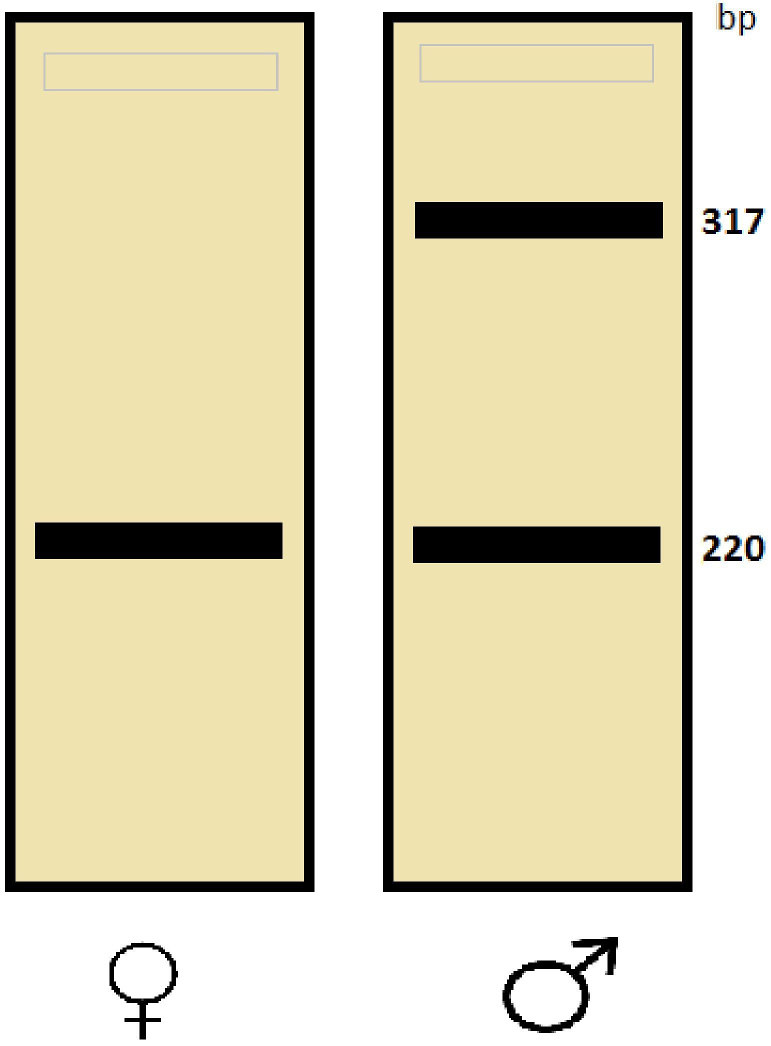

European rabbit (*Oryctolagus cuniculus*). *ZFX, ZFY*

Fontanesi et al., 2008

F: 5’-GGTGCAGCAACATGCTCTTA-3’

R: 5’-TTAAAGCCTGAGGCGTCTGT-3’

```
ZFX TCAGATACCAAAGAGGTGCAGCAACATGCTCTTATCCACCAAGAAAGCAAAACACACCAG
ZFY -----------------------------------TCCACCAAGAGAGCAAAACACACCAG

ZFX TGTTTGCATTGTGACCACAAGAGTTCGAACTCAAGTGATTTGAAACGACACATTATTTCA
ZFY TGTTTGCATTGTGACCACAAGAGTTCGAACTCAAGTGATTTGAAACGACACATTATTTCA

ZFX GTTCACACAAAGGACTACCCCCACAAGTGTGACATGTGTGATAAAGGCTTTCACCGGCCT
ZFY GTTCACACAAAGGACTACCCCCACAAATGTGACATGTGTGATAAAGGCTTTCACCGGCCT

ZFX TCAGAACTCAAGAAACACGTGGCTGCCCACAAGGGTAAAAAAATGCACCAGTGTAGACAT
ZFY TCAGAACTCAAGAAACACGTGGCTGCTCACAAGGGTAAAAAAATGCATCAGTGTAGACAT

ZFX TGTGACTTTAAGATTGCAGATCCATTTGTTCTAAGTCGCCATATTCTATCAGTTCACACA
ZFY TGTGACTTTAAGATTGCAGATCCATTTGTTCTGAGTCGCCATATTCTCTCAGTTCACACA
                                 ▲***

ZFX AAGGATCTTCCATTTAGGTGTAAGAGATGTAGAAAGGGATTTAGACAACAGAATGAGCTT
ZFY AAAGATCTTCCATTTAGGTGTAAGAGATGTAGAAAGGGATTTAGACAACAGAATGAACTT
     ▲****                                                ****

ZFX AAAAAGCATATGAAGACACACAGTGGCAGGAAAGTGTATCAGTGTGAGTACTGTGAGTAT
ZFY AAAAAGCATATGAAGACACACAGTGGCAGGAAAGTGTATCAGTGTGAGTACTGTGAGTAT

ZFX AGCACTACAGACGCCTCAGGCTTTAAACGGCATGTTATTTCCATTCATACGAAGGACTAT
ZFY AGCACT------------------------------------------------------
```

Primers | AluI | BglII | HinfI | Restriction site ▲ | Diagnostic site ******

RE recognition sites:

AluI: 5′…AG▼CT…3′ / 3′…TC▲GA…5′

BglII: 5′…A▼GATCT…3′ / 3′…TCTAG▲A…5′

HinfI: 5′…G▼ANTC…3′ / 3′…CTNA▲G…5′

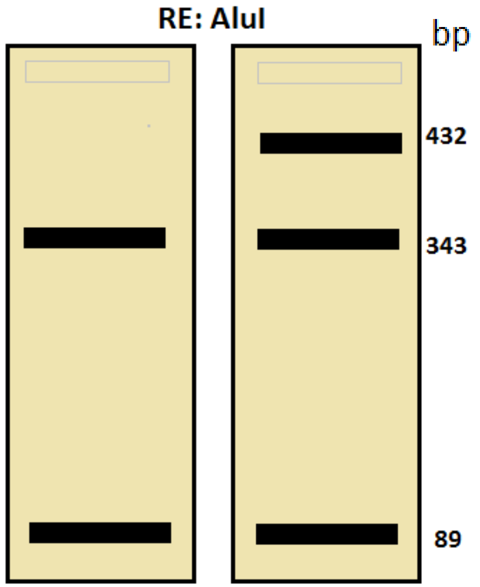

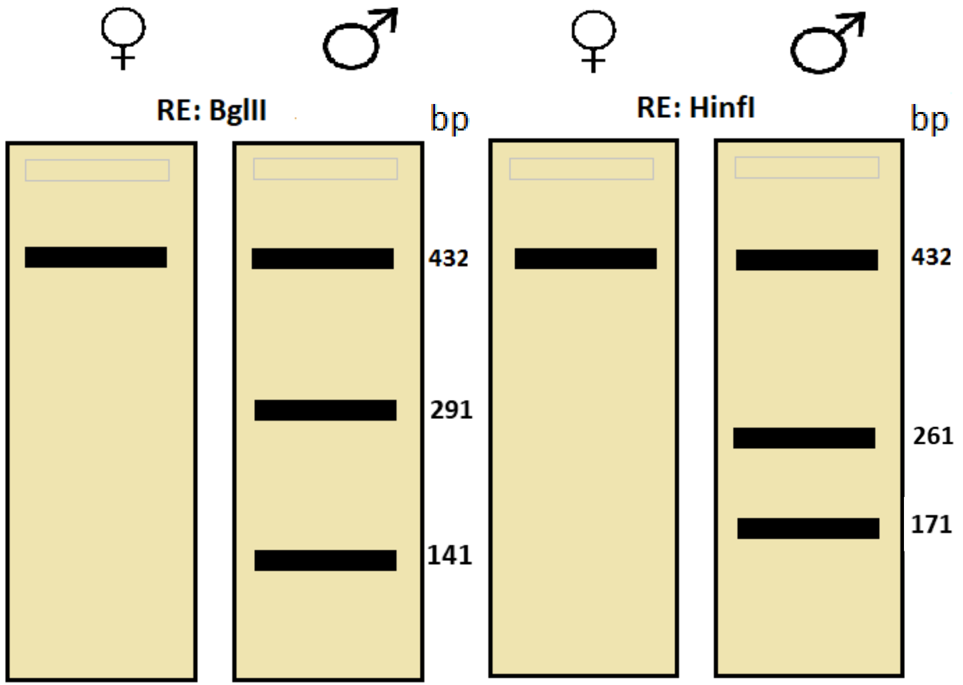

EMBL acc. no:.: XM_017349827. 1, AM778418.1

European brown hare (*Lepus europaeus*). *ZFX, ZFY*

Fontanesi et al., 2008

F: 5'-GGTGCAGCAACATGCTCTTA-3'

R: 5'-TTAAAGCCTGAGGCGTCTGT-3'

```
ZFX  1    TCCACCAAGAAAGCAAAACACACCAGTGTTTGCATTGTGACCACAAGAGTTCGAACTCAA  60
          |||||||||| |||||||||||||||||||||||||||||||||||||||||||||||||
ZFY  1    TCCACCAAGAGAGCAAAACACACCAGTGTTTGCATTGTGACCACAAGAGTTCGAACTCAA  60

ZFX  61   GTGATTTGAAACGACACATTATTTCAGTTCACACAAAGGACTACCCCCACAAGTGTGACA  120
          |||||||||||||||||||||||||||||||||||||||||||||||||||| |||||||
ZFY  61   GTGATTTGAAACGACACATTATTTCAGTTCACACAAAGGACTACCCCCACAAATGTGACA  120

ZFX  121  TGTGTGATAAAGGCTTTCACCGGCCTTCAGAACTCAAGAAACACGTGGCTGCCCACAAGG  180
          |||||||||||||||||||||||||||||||||| |||||||| |||||||| |||||||
ZFY  121  TGTGTGATAAAGGCTTTCACCGGCCTTCAGAACTTAAGAAACATGTGGCTGCTCACAAGG  180

ZFX  181  GTaaaaaaaTGCACCAGTGTAGACATTGTGACTTTAAGATTGCAGATCCATTTGTTCTGA  240
          ||||||||||||| ||||||||||||||||| ||||||||||||||||||||||||||||
ZFY  181  GTAAAAAAATGCATCAGTGTAGACATTGTGATTTTAAGATTGCAGATCCATTTGTTCTGA  240

ZFX  241  GTCGCCATATTCTGTCAGTTCACACAAAGGATCTTCCATTTAGGTGTAAGAGATGTAGAA  300
          ||||||||||||| |||||||||||||| |||||||||||||||||||||||||||||||
ZFY  241  GTCGCCATATTCTCTCAGTTCACACAAAAGATCTTCCATTTAGGTGTAAGAGATGTAGAA  300

ZFX  301  AGGATTTAGACAACAGAATGAGCTTAAAAAGCATATGAAGACACACAGTGGCAGGAAAG  360
          ||||||||||||||||||||| ||||||||||||||||||||||||||||||||||||||
ZFY  301  AGGATTTAGACAACAGAATGAACTTAAAAAGCATATGAAGACACACAGTGGCAGGAAAG  360

ZFX  361  TGTATCAGTGTGAGTACTGTGAGTATAGCACT  392
          ||||||||||||||||||||||||||||||||
ZFY  361  TGTATCAGTGTGAGTACTGTGAGTATAGCACT  392
```

AluI BglII Restriction site ▲ Diagnostic site ******

RE recognition sites:

AluI: 5'...AGCT...3' / 3'...TCGA...5'

BglII: 5'...AGATCT...3' / 3'...TCTAGA...5'

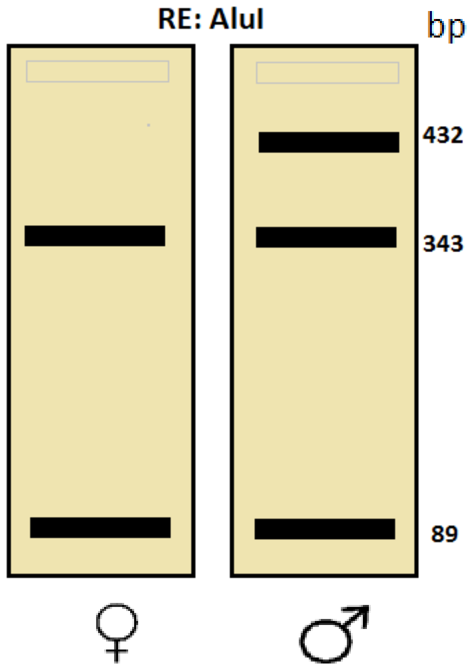

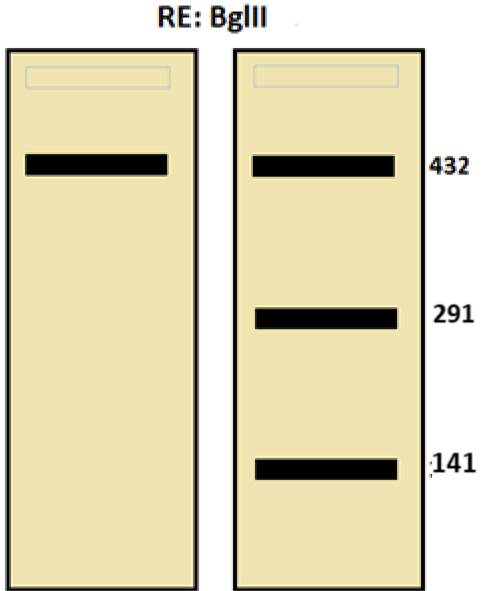

EMBL acc. no.: AM778429.1 AM778430.1

Mountain hare (*Lepus timidus*): ZFX, ZFY

Fontanesi et al., 2008

F: 5’-GGTGCAGCAACATGCTCTTA-3’

R: 5’-TTAAAGCCTGAGGCGTCTGT-3’

```
ZFX   1   TCCACCAAGAGAGCAAAACACACCAGTGTTTGCATTGTGACCACAAGAGTTCGAACTCAA  60
          ||||||||||||||||||||||||||||||||||||||||||||||||||||||||||||
ZFY   1   TCCACCAAGAGAGCAAAACACACCAGTGTTTGCATTGTGACCACAAGAGTTCGAACTCAA  60

ZFX  61   GTGATTTGAAACGACACATTATTTCAGTTCACACAAAGGACTACCCCCACAAGTGTGACA  120
          |||||||||||||||||||||||||||||||||||||||||||||||||||| |||||||
ZFY  61   GTGATTTGAAACGACACATTATTTCAGTTCACACAAAGGACTACCCCCACAAATGTGACA  120

ZFX 121   TGTGTGATAAAGGCTTTCACCGGCCTTCAGAACTCAAGAAACACGTGGCTGCCCACAAGG  180
          ||||||||||||||||||||||||||||||||||||||||||||||||||||||||||||
ZFY 121   TGTGTGATAAAGGCTTTCACCGGCCTTCAGAACTCAAGAAACACGTGGCTGCCCACAAGG  180

ZFX 181   GTaaaaaaaTGCACCAGTGTAGACATTGTGACTTTAAGATTGCAGATCCATTTGTTCTGA  240
          ||||||||||||||||||||||||||||||||||||||||||||||||||||||||||||
ZFY 181   GTAAAAAAATGCACCAGTGTAGACATTGTGACTTTAAGATTGCAGATCCATTTGTTCTGA  240

ZFX 241   GTCGCCATATTCTGTCAGTTCACACAAAGGATCTTCCATTTAGGTGTAAGAGATGTAGAA  300
          ||||||||||||| |||||||||||||||  |||||||||||||||||||||||||||||
ZFY 241   GTCGCCATATTCTCTCAGTTCACACAAAAGATCTTCCATTTAGGTGTAAGAGATGTAGAA  300
                                     ▼    *▲*****
ZFX 301   AGGGATTTAGACAACAGAATGAGCTTAAAAAGCATATGAAGACACACAGTGGCAGGAAAG  360
          |||||||||||||||||||||| |||||||||||||||||||||||||||||||||||||
ZFY 301   AGGGATTTAGACAACAGAATGAACTTAAAAAGCATATGAAGACACACAGTGGCAGGAAAG  360
                              ****
ZFX 361   TGTATCAGTGTGAGTACTGTGAGTATAGCACT  392
          ||||||||||||||||||||||||||||||||
ZFY 361   TGTATCAGTGTGAGTACTGTGAGTATAGCACT  392
```

AluI BglII Rrestriction site ▲ Diagnostic site ******

RE recognition sites:

AluI: 5′...AG▼CT...3′ / 3′...TC▲GA...5′

*Bgl*II: 5′...A▼GATCT...3′ / 3′...TCTAG▲A...5′

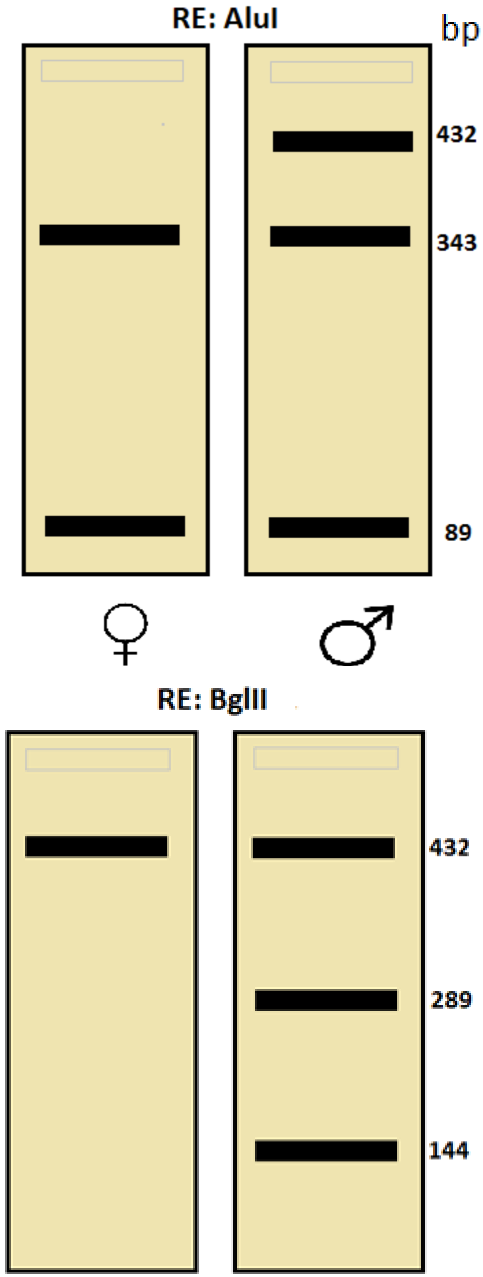

EMBL acc. no.: AM778431.1 AM778432.1

Lesser horseshoe bat (*Rhinolophus hipposideros*). *DDX3X, DDX3Y*

Zarzoso-Lacoste et al., 2018

DDX3X/Y-Mam-F: CAGATCTATGAGGAAGCCAGAAA

DDX3X/Y-Mam-R: TCATACCGCTCTAGAGTTCGC

```
DDX3X  1    CAGATCTATGAGGAAGCCAGAAAGGTAAGGATGCATTAAAGTG---ATTGGCTTTCTCAT  57
            ||||||||||||||||||||||| ||||  |  ||||  | ||   |||||| || | ||
DDX3Y  1    CAGATCTATGAGGAAGCCAGAAAAGTAAATACACATTTTATTGGTAATTGGCATTTTTAT  60

DDX3X  58   TGATTCTAATGAAATGTTTTATGCGCACGTAAAAGTCTCAGCCTTGAAGTTGATGAAATT  117
            |||||||||||       |||||  ||| ||||                        | |
DDX3Y  61   TGATTCTAATGCC-----TTATGATCACCTAAAT------------------------AAT  92
                         *****                ***********************

DDX3X  118  TCTTTGCTTATAGTTTTCATACCGCTCTAGAGTTCGC  154
            ||||| |||||||||||||||||||||||||||||||
DDX3Y  93   TCTTTTCTTATAGTTTTCATACCGCTCTAGAGTTCGC  129
Primers  SSIndel  ****
```

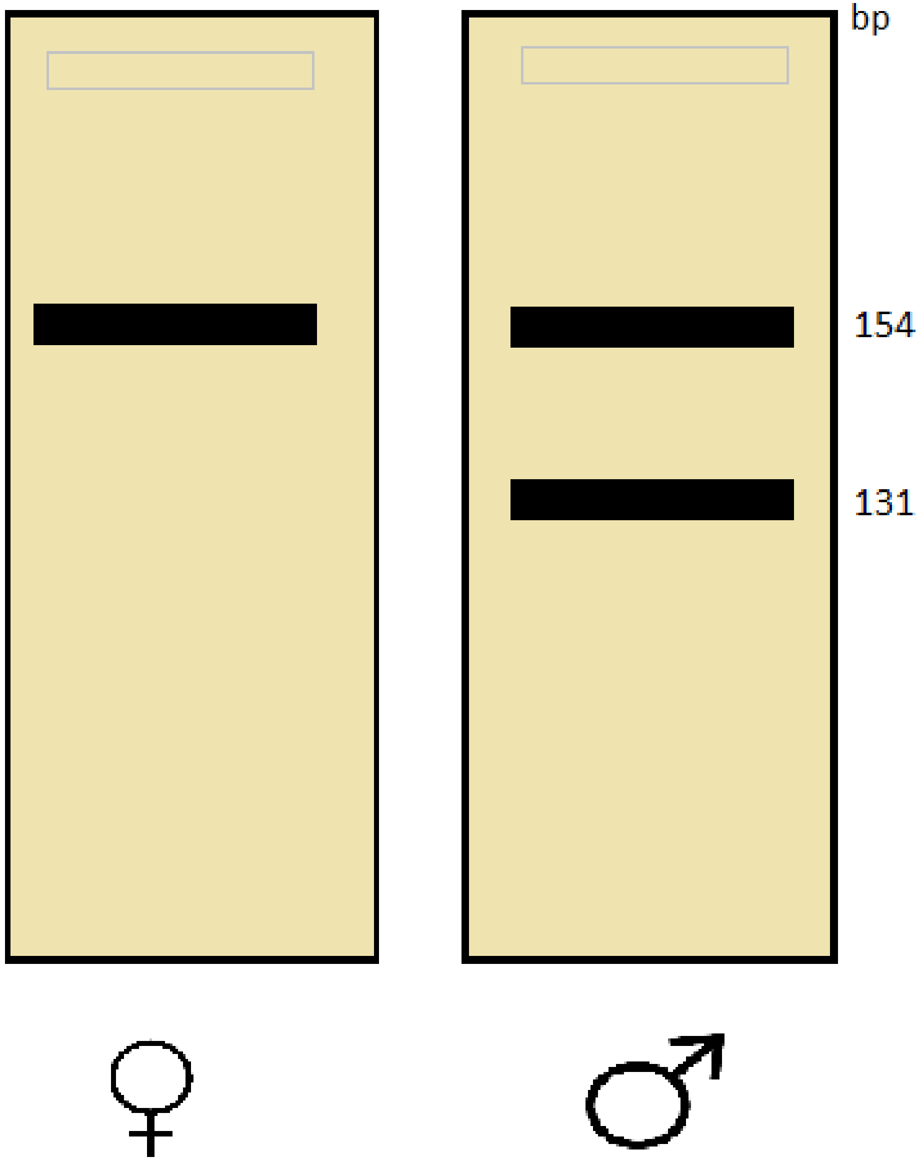

EMBL acc. No.: KY793698.1, KY793697.1

Silver-haired bat (*Lasionycteris noctivagans*), eastern red bat (*Lasiurus borealis*), hoary bat (*Lasionycteris cinereus*), evening bat (*Nycticeius humeralis*), tri-colored bat (*Perimyotis subflavus*), Mexican free-tailed bat (*Tadarida brasiliensis*). *ZFX, ZFY.*

Korstian et al., 2013

ZFX:
F:5′-AGTCAAGGGRTGTCCATCR-3′
R:5′-GTTTGYASACCAGGTTCCT-3′
ZFY:
F:5′-GGTRAGDGCACAYRAGTTCCACA-3′
R:5′-TGCYATTACAAAACCTTTRTAGATAC-3′
T. brasiliensis ZFY:
F:5′-GGTGAGGGCACATGAGTTCCACA-3′
R:5′-TGCCATCACAATACCCTCTGTGATAC-3′

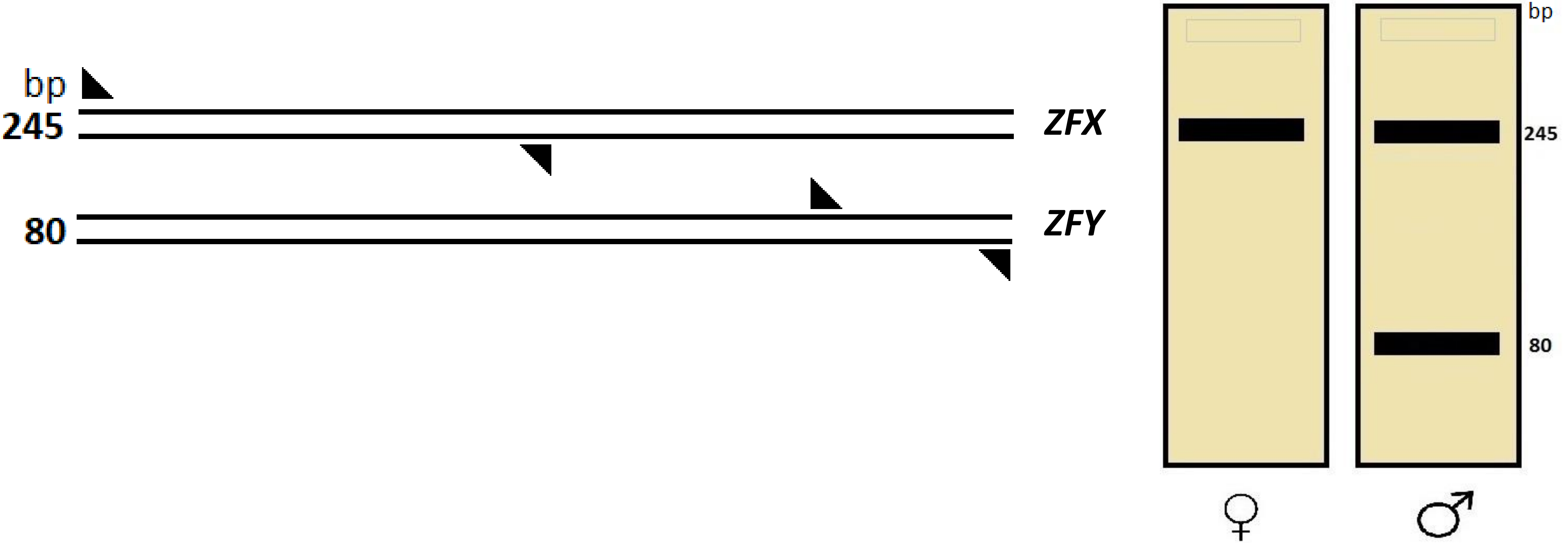

DeCandia et al., 2016

Carnivora: **Mediterranean monk seal (*Monachus monachus*), Hawaiian monk seals (*Monachus schauinslandi*), puma (*Puma concolor*), Pallas's cat (*Otocolobus manul*), jaguar (*Panthera onca*), tiger (*Panthera tigris*), lion (*Panthera leo*), serval (*Leptailurus serval*), bobcat (*Lynx rufus*), wolf (*Canis lupus*), domestic dog (*Canis lupus familiaris*), coyote (*Canis latrans*). *ZFX, SRY***

SRY

CSRY1F: 5'-CGCAAGGTGGCTCTAGAG-3'

CSRY1R: 5'-GCCTTSCGACGAGGTCGGTA-3'

ZFX

ZFX1F: 5'-TGGCCCAGATGGACATCC-3'

ZFX1R: 5'-AATGCTTTCCGGACTCATCG-3'

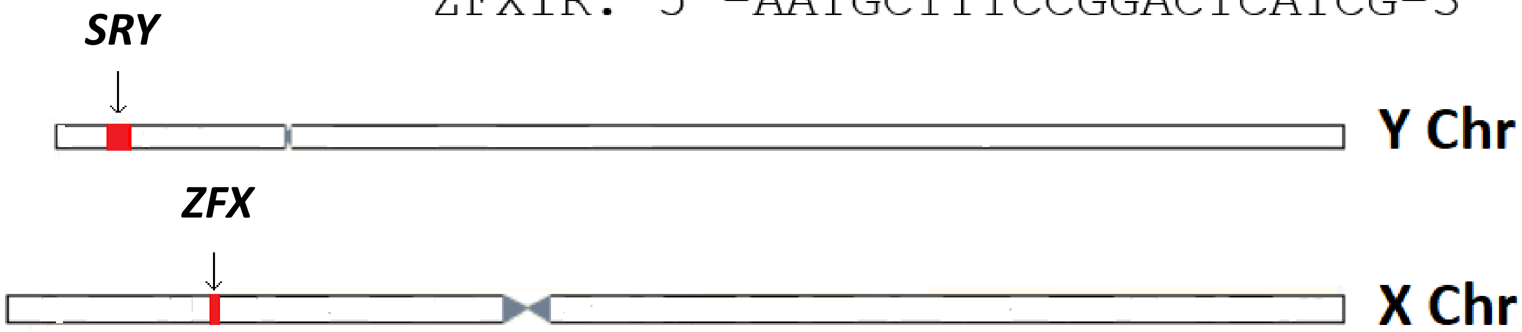

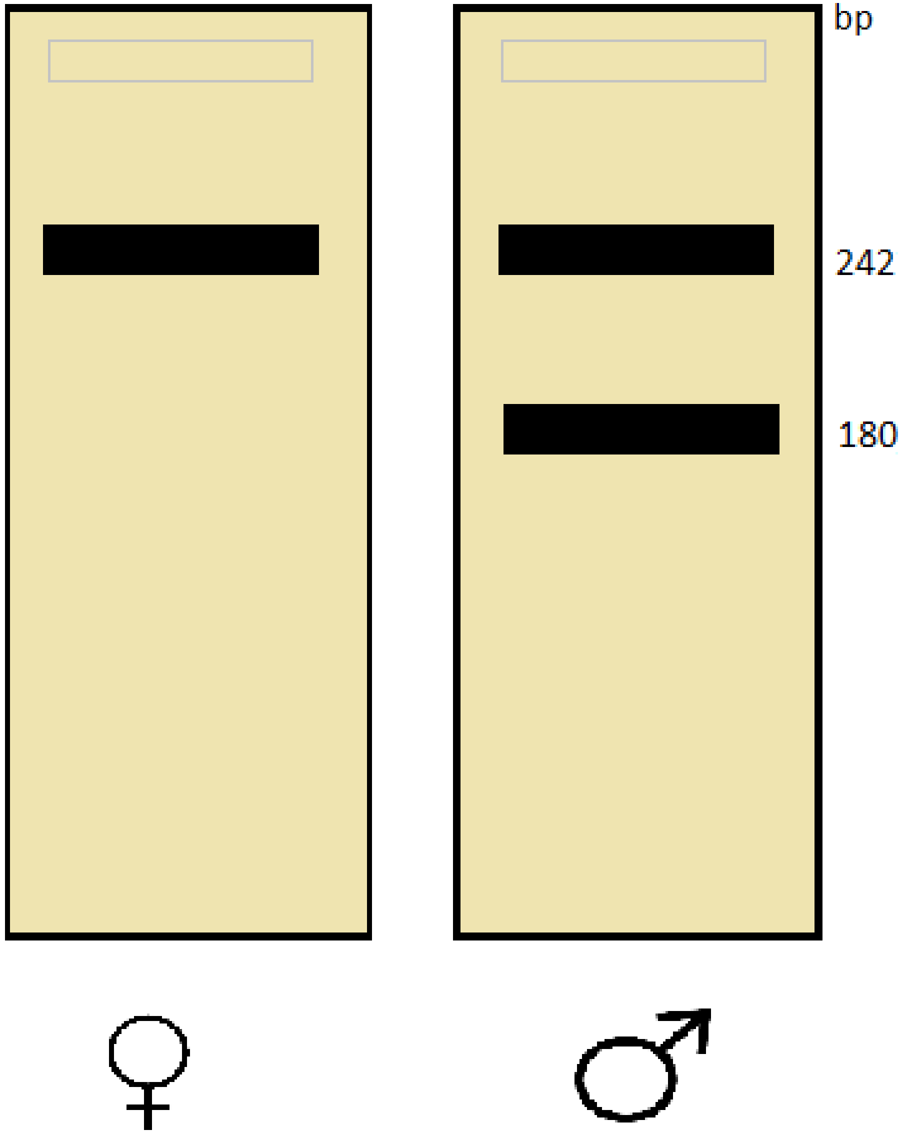

Domestic cat (*Felis silvestris*). *AMELX, AMELY*

Pilgrim et al., 2005

F: 5′-CGAGGTAATTTTTCTGTTTACT-3′

R: 5′-GAAACTGAGTCAGAGAGGC-3′

```
AMELX   1   GTTAYATCAACTTCAGCTATGAGGTAATTTTTCTCTTTACTAATTTTGACCATTGTTTGA   60
            |||| |||||||||||||| ||||||||||||||||||||||| ||||| |||||||
AMELY   1   GTTATATCAACTTCAGCTACGAGGTAATTTTTCTCTTTACTAACTTTGATCATTGTTACG   60

AMELX  61   CTTAACAATGCCCTGGGCTCTGTAAAGAGTAGCGTGTTGATTCTTTGTTCAAGATGTTTC  120
             |||||||| |||||||||||||||||| |||  |||||||||||| |||||||||||||
AMELY  61   GTTAACAATTCCCTGGGCTCTGTAAAGAATAGTATGTTGATTCTTTATTCAAGATGTTTC  120

AMELX 121   TCAGTCCCGCTTTTTCAGTTCCCATTACCAGCTTCCTGGTTTAAGCCCTGATGGGTTGCC  180
            ||||||| ||||||||||||| ||||||||||| ||||||||
AMELY 121   TCAGTCCTGCTTTTTCAGTTCTCATTACCAGCTGCCTGGTTT------------------  162
                                                      ******************

AMELX 181   TGAAGCCCGCATTGCCCCCAGCACACTCCTGCTCGGCCTCTCTGACTCAGTTTCTCCTCC  240
              ||||| ||||||  |||||||||||||||  |  |||||||||||||||||||||||| |
AMELY 163   --AAGCCTGCATTG-TCCCAGCACACTCCTATTTGGCCTCTCTGACTCAGTTTCTCCTGC  219
            **            *
```

Primers · Mismatch to primer · SSIndel ******

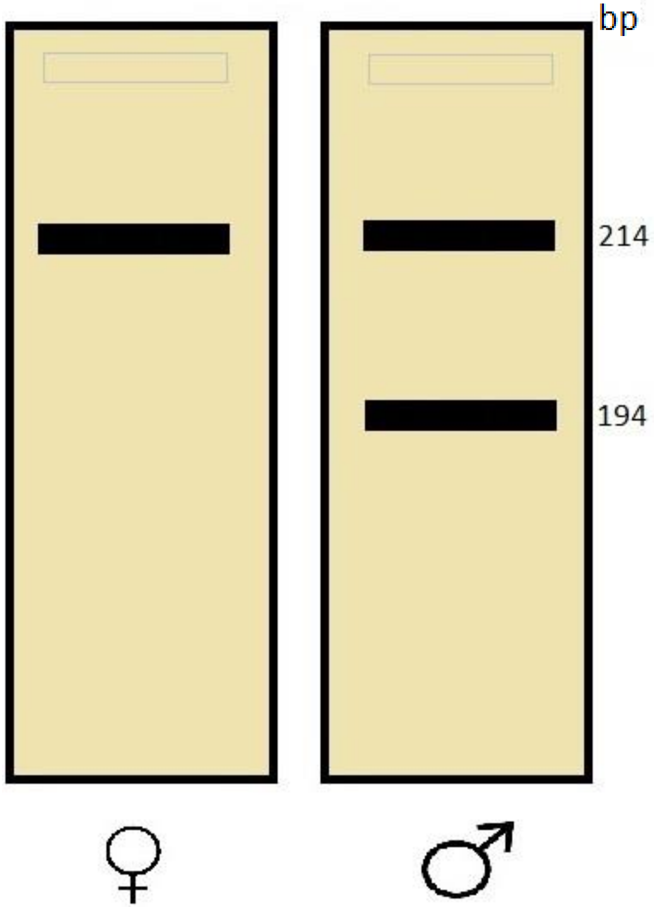

EMBL acc. No.: AF114709, AF197967

Domestic cat (*Felis silvestris*). *ZFX, ZFY*

Pilgrim et al., 2005

F: 5′-AAGTTTACACAACCACCTGG-3′
R: 5′-CACAGAATTTACACTTGTGCA-3′

```
ZFX  121  CCAAGAAGAAGTACCGCTGTACTGACTGTGATTACACTACCAACAAGAAGATAAGTTTAC  180
          ||||||||||||||| ||||||||||||||||||||||||||||||||||||||||||||
ZFY  121  CCAAGAAGAAGTACCACTGTACTGACTGTGATTACACTACCAACAAGAAGATAAGTTTAC  180

ZFX  181  ACAACCACCTGGAGAGCCACAAGCTGACCAGCAAGGCCGAGAAGGCCATCGAATGCGATG  240
          ||||||||||||||||||||||||| ||||||||||||||||||||||||||||| ||||
ZFY  181  ACAACCACCTGGAGAGCCACAAGCTCACCAGCAAGGCCGAGAAGGCCATCGAATGTGATG  240

ZFX  241  AGTGTGGGAAGCATTTCTCTCACGCTGGGGCTTTGTTTACTCACAAAATGGTGCATAAGG  300
          |||||||||||||||||||||| |||||||||||||||||||||||||||||||||||
ZFY  241  AGTGTGGGAAGCATTTCTCTCATGCTGGGGCTTTGTTTACTCACAAAATGGTGCATAA--  298
                                                                    **

ZFX  301  AGAAAGGAGCCAACAAAATGCACAAGTGTAAATTCTGTGAATACGAGACAGCTGAACAAG  360
          |||||  ||||||||||||||||||||||||||||||||||| ||||||||||||||||
ZFY  299  AGAAA-AAGCCAACAAAATGCACAAGTGTAAATTCTGTGAATATGAGACAGCTGAACAAG  357
               *
```

Primers SSIndel******

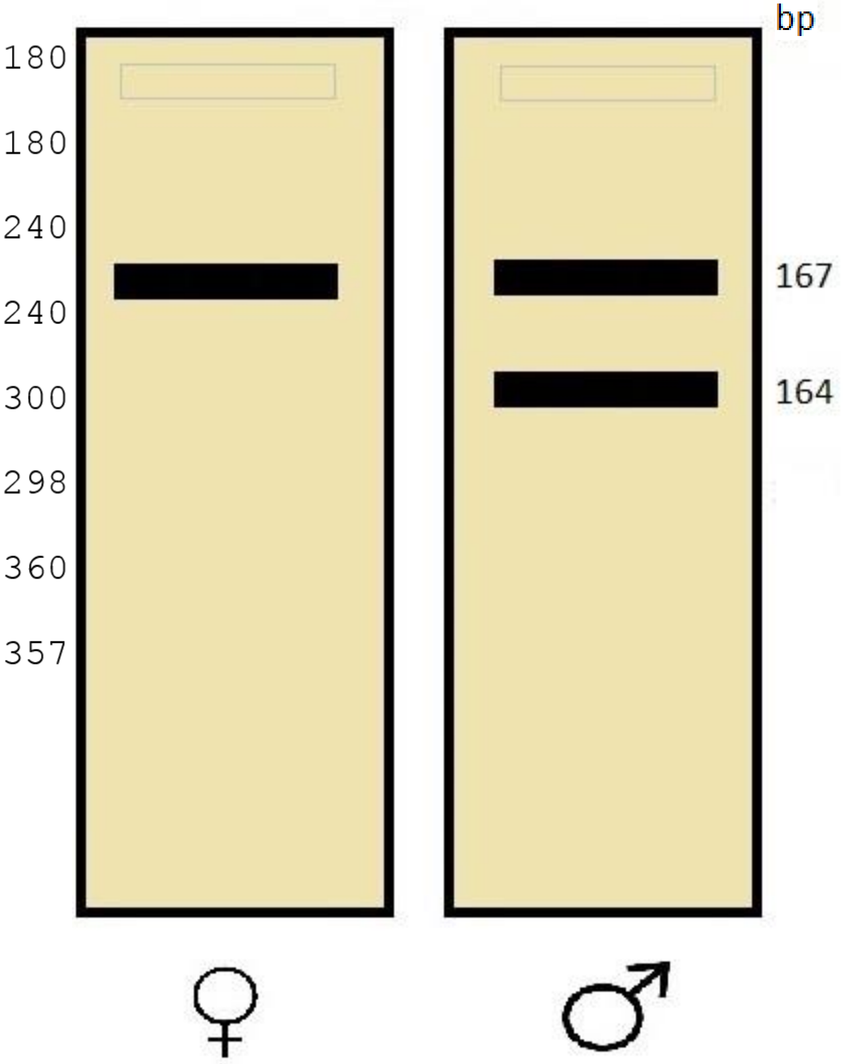

EMBL acc. No.: AF253014, AF252989

Cougar (*Puma concolor*). *ZFX, ZFY*

Pilgrim et al., 2005

F: 5′-AAGTTTACACAACCACCTGG-3′

R: 5′-CACAGAATTTACACTTGTGCA-3′

```
ZFX  121  CCAAGAAGAAGTACCGCTGTACTGACTGTGATTACACTACCAACAAGAAGATAAGTTTAC  180
          ||||||||||||||| ||||||||| ||||||||||||||||||||||||||||||||||
ZFY  121  CCAAGAAGAAGTACCACTGTACTGATTGTGATTACACTACCAACAAGAAGATAAGTTTAC  180

ZFX  181  ACAACCACCTGGAGAGCCACAAGCTGACCAGCAAGGCCGAGAAGGCCATCGAATGCGATG  240
          ||||||||||||||||||||||||| ||||||||||| ||||||||||||||||| ||||
ZFY  181  ACAACCACCTGGAGAGCCACAAGCTCACCAGCAAGGCTGAGAAGGCCATCGAATGTGATG  240

ZFX  241  AGTGTGGGAAGCATTTCTCTCACGCTGGGGCTTTGTTTACTCACAAAATGGTGCATAAGG  300
          |||||||||||||||||||||| |||||||||||||||||||||||||||||||||||
ZFY  241  AGTGTGGGAAGCATTTCTCTCATGCTGGGGCTTTGTTTACTCACAAAATGGTGCATAA--  298
                                                                    **

ZFX  301  AGAAAGGAGCCAACAAAATGCACAAGTGTAAATTCTGTGAATACGAGACAGCTGAACAAG  360
          |||||  |||||||||||||||||||||||||||||||||||| ||||||||||||||||
ZFY  299  AGAAA-AAGCCAACAAAATGCACAAGTGTAAATTCTGTGAATATGAGACAGCTGAACAAG  357
               *
```

Primers SSIndel******

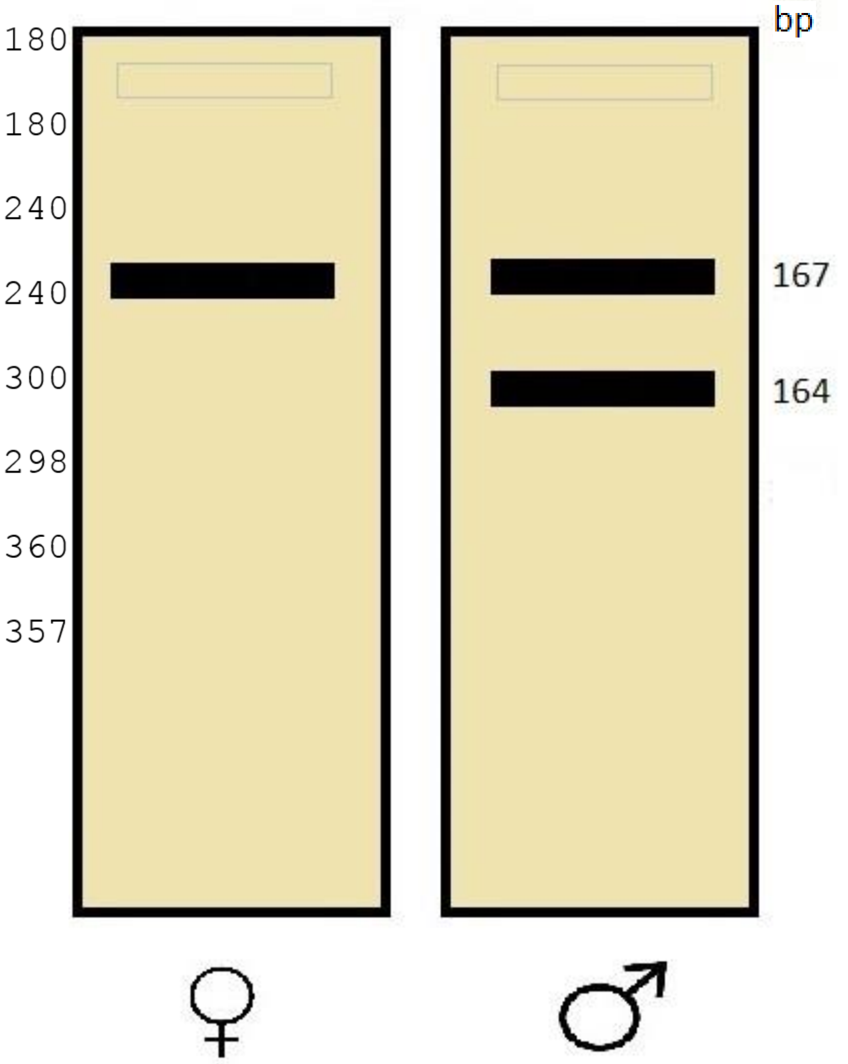

EMBL acc. No.: AF253018, AF252988

Bobcat (*Lynx rufus*). *ZFX, ZFY*

Pilgrim et al., 2005

F:5′-AAGTTTACACAACCACCTGG-3′

R:5′-CACAGAATTTACACTTGTGCA-3′

```
ZFX  121   CCAAGAAGAAGTACCGCTGTACTGACTGTGATTACACTACCAACAAGAAGATAAGTTTAC  180
           ||||||||||||||| ||||||||||||||||||||||||||||||||||||||||||||
ZFY  121   CCAAGAAGAAGTACCACTGTACTGACTGTGATTACACTACCAACAAGAAGATAAGTTTAC  180

ZFX  181   ACAACCACCTGGAGAGCCACAAGCTGACCAGCAAGGCCGAGAAGGCCATCGAATGCGATG  240
           ||||||||||||||||||||||||| ||||||||||||||||||||||||||||| ||||
ZFY  181   ACAACCACCTGGAGAGCCACAAGCTCACCAGCAAGGCCGAGAAGGCCATCGAATGTGATG  240

ZFX  241   AGTGTGGGAAGCATTTCTCTCACGCTGGGGCTTTGTTTACTCACAAAATGGTGCATAAGG  300
           |||||||||||||||||||||| ||||||||||||||||||||||||||||||||||| |
ZFY  241   AGTGTGGGAAGCATTTCTCTCATGCTGGGGCTTTGTTTACTCACAAAATGGTGCATAAAG  300

ZFX  301   AGAAAGGAGCCAACAAAATGCACAAGTGTAAATTCTGTGAATACGAGACAGCTGAACAAG  360
           | ||||   |||||||||||||||||||||||||||||||||| ||||||||||||||||
ZFY  301   AAAAAG---CCAACAAAATGCACAAGTGTAAATTCTGTGAATATGAGACAGCTGAACAAG  357
                 ***
Primers  SSIndel******
```

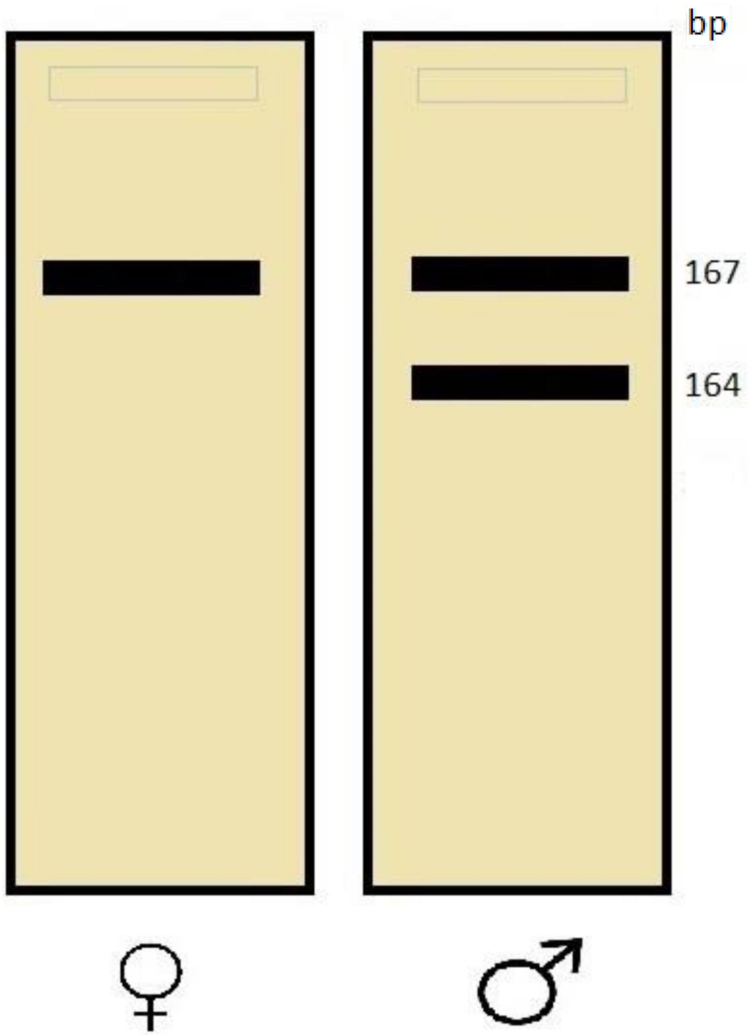

EMBL acc. No.: AF253002, AF252975

Eurasian lynx (*Lynx lynx*). *ZFX, ZFY*

Pilgrim et al., 2005

F:5′-AAGTTTACACAACCACCTGG-3′
R:5′-CACAGAATTTACACTTGTGCA-3′

```
ZFX  121   CCAAGAAGAAGTACCGCTGTACTGACTGTGATTACACTACCAACAAGAAGATAAGTTTAC  180
           ||||||||||||||| ||||||||||||||||||||||||||||||||||||||||||||
ZFY  121   CCAAGAAGAAGTACCACTGTACTGACTGTGATTACACTACCAACAAGAAGATAAGTTTAC  180

ZFX  181   ACAACCACCTGGAGAGCCACAAGCTGACCAGCAAGGCCGAGAAGGCCATCGAATGCGATG  240
           ||||||||||||||||||||||||| |||||||||||||||||||||||||||||| ||||
ZFY  181   ACAACCACCTGGAGAGCCACAAGCTCACCAGCAAGGCCGAGAAGGCCATCGAATGTGATG  240

ZFX  241   AGTGTGGGAAGCATTTCTCTCACGCTGGGGCTTTGTTTACTCACAAAATGGTGCATAAGG  300
           ||||||||||||||||||||||| ||||||||||||||||||||||||||||||||||||
ZFY  241   AGTGTGGGAAGCATTTCTCTCATGCTGGGGCTTTGTTTACTCACAAAATGGTGCATAAGG  300

ZFX  301   AGAAAGGAGCCAACAAAATGCACAAGTGTAAATTCTGTGAATACGAGACAGCTGAACAAG  360
           | |||  |||||||||||||||||||||||||||||||||||| ||||||||||||||||
ZFY  301   A-AAA--AGCCAACAAAATGCACAAGTGTAAATTCTGTGAATATGAGACAGCTGAACAAG  357
            *    **
```

Primers SSIndel******

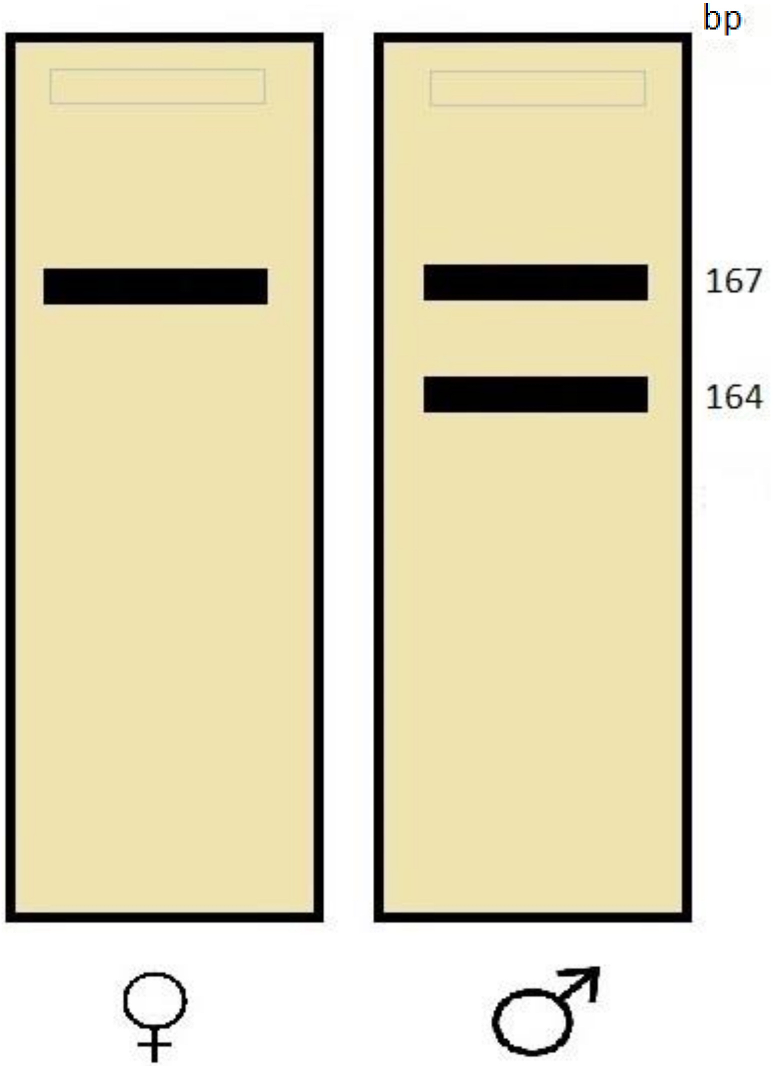

EMBL acc. No.: AF253001, AF252974

Masked palm civet (*Paguma larvata*). *ZFX, SRY*

Zhang et al., 2016

SRY

F: ATTCTTCGAGGAGGCACA

R: GGGTTCTGAGGAGGAGTTT

ZFX

F: TTGGCGGTCCACAGCAAGAAC

R: CTGGCACTGGTATGGCTTCTC

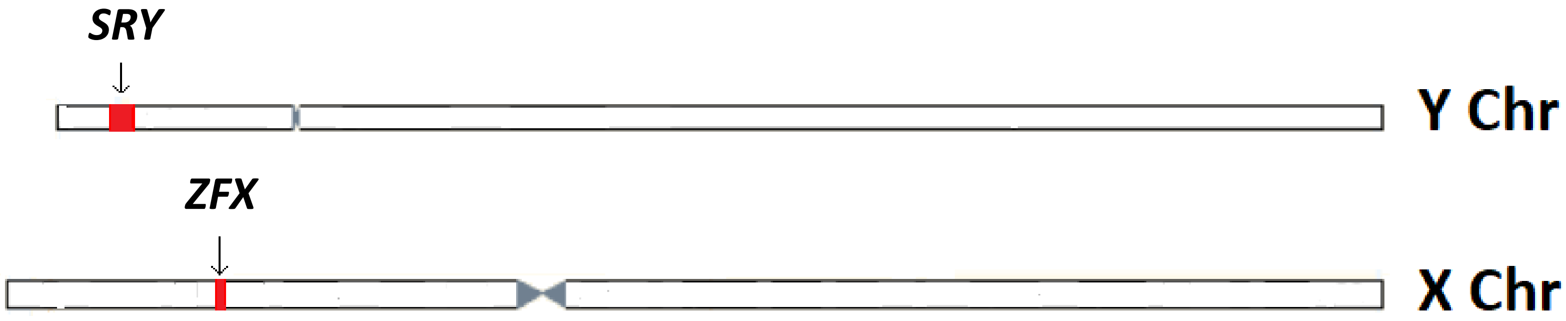

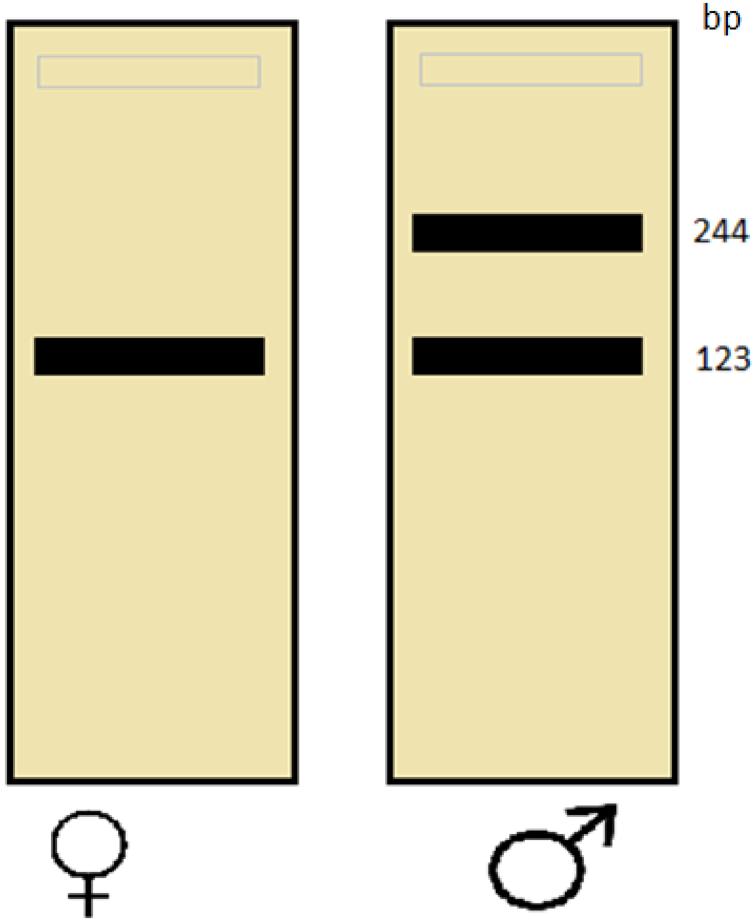

Sea otter (*Enhydra lutris*). *ZFX*, *ZFY*

Hattori et al., 2003

Outer primers:

F:5′-ATAATCACATGGAGAGCCACAAGCT-3′

R:5′-GCACTTCTTTGGTATCTGAGAAAGT-3′

Inner primers:

F:5′-TGACCAGCAAGGCAGAGAA-3′

R:5′-TCAACAACCCTTGTTCAGCT-3′

*Nla*III recognition site:

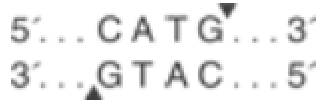

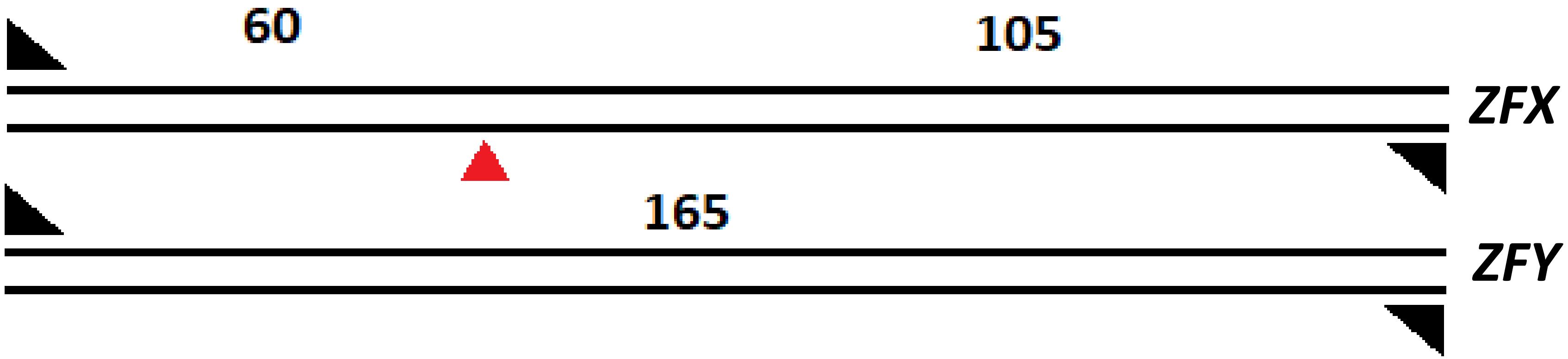

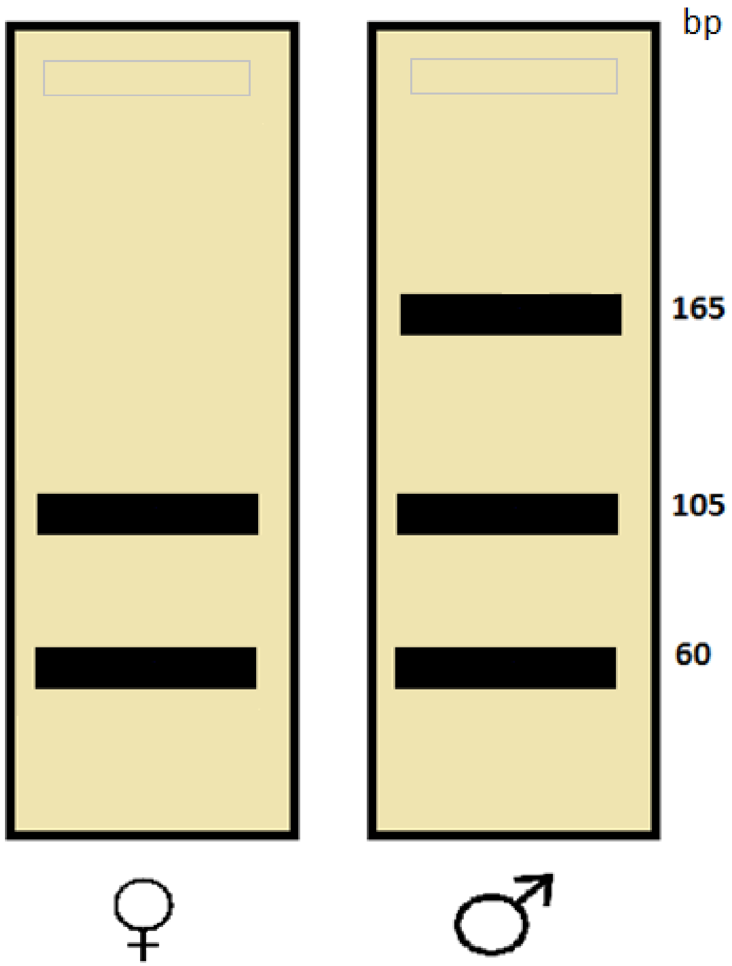

Domestic dog (*Canis lupus familiaris*). *AMELX, AMEY*

Yan et al., 2013

F:5′-ATAATGACAAAGAAAACATGAC-3′
R:5′-CTGCTGAGCTGGCACCAT-3′

```
AMELX  7828116   aTGAATAACTATTCATAGTA--ATAATGACAAAGAAAACATGACTACTTCTCCAGCTGGA   7828173
                  || |||| |||||||| ||  ||||||||||||||||||||||||  ||||| | ||||
AMELY  1385      -TGGATAAATATTCATACTATCATAATGACAAAGAAAACATGACTATATCTCCTGTTGGA   1443

AMELX  7828174   AGTCAATG--------------------------------GAGCCGATGGTAAACCTGAC   7828201
                 ||||| |                                 ||||  ||| | |||||||
AMELY  1444      AGTCAGTTTGACTGAGTCACTGTAACTGAGTCAGTTGTGTGAGCTAATGATGAACCTGAT   1503
                         ********************************

AMELX  7828202   TCTTTGTTTCTCACCAGTACCCTTCCTATGGTTACGAACCCATGGGTGGATGGCTGCACC   7828261
                 |||||||||| |||||||||||||||||||||||||||||||||||||| ||||||||||
AMELY  1504      TCTTTGTTTCCCACCAGTACCCTTCCTATGGTTACGAACCCATGGGTGGTTGGCTGCACC   1563

AMELX  7828262   ACCAAATCATTCCCGTGCTGTCCCAGCAAAATCCCTCGAATCACGCCCTGCAGCCTCATC   7828321
                 |||||||||||||| |||||||||||||||||||||||||||| ||||||||||||||||
AMELY  1564      ACCAAATCATTCCCATGCTGTCCCAGCAAAATCCCTCGAATCAAGCCCTGCAGCCTCATC   1623

AMELX  7828322   ACCACATCCCCATGGTGCCAGCTCAGCAGCCCGTGGTCCCCCAGCAACCAATGATGCCAG   7828381
                 | |||||| ||||||||||||||||||||| |||||||||||||||||||||||||||||
AMELY  1624      ATCACATCTCCATGGTGCCAGCTCAGCAGCCTGTGGTCCCCCAGCAACCAATGATGCCAG   1683
Primers SSIndel  ******
```

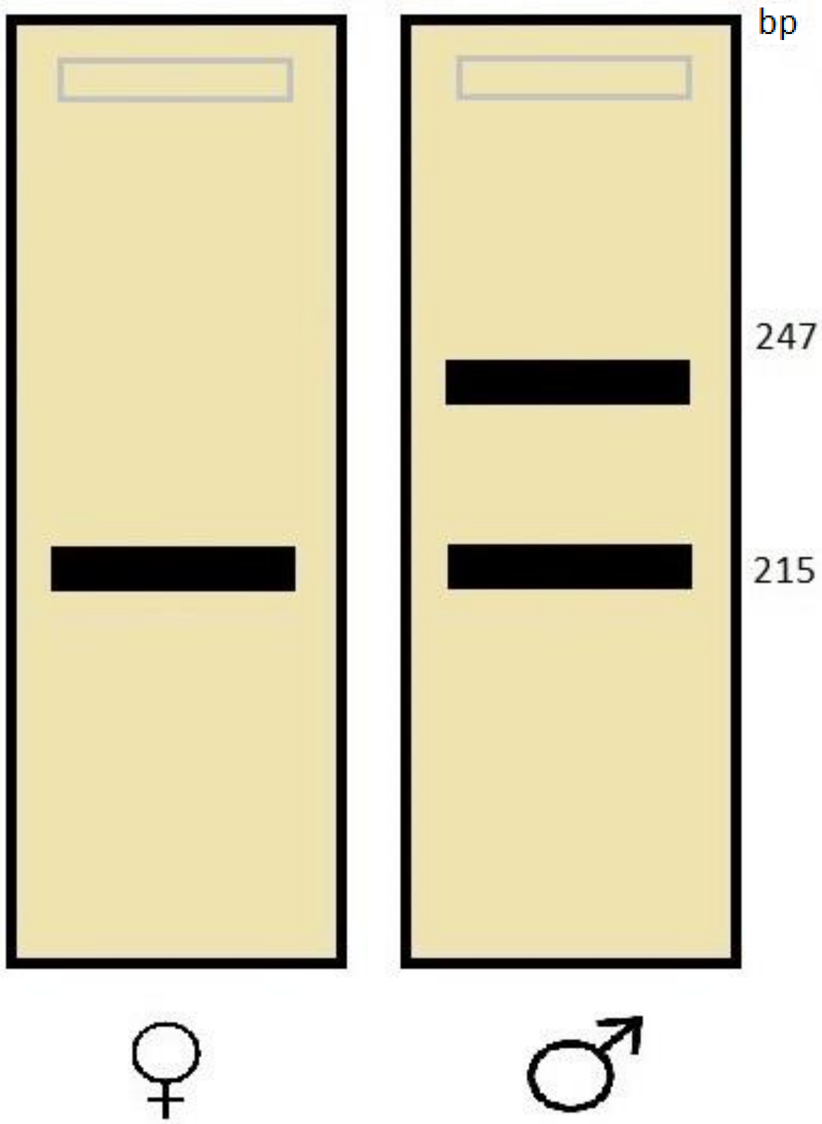

EMBL acc. No.: NC_006621, KC763835

Domestic dog (*Canis lupus familiaris*). *ZFX, ZFY*

Ortega et al., 2004

F: 5′-CAAAAGGTGGCGATTCAATAA-3′

R: 5′-ATGGAGAGCCACAAGCTRAC-3′

*Taq*I recognition site: 5′...TCGA...3′ / 3′...AGCT...5′

```
ZFX  188  GGAAAGTTCTTGCTGTGGACCGCCAAAAGGTGGCGATTCAATAACCCTTGTTCAGCTGTC  247
          ||||||||||||||||| |||||||||||||||||||||||||| |||||||||||||||
ZFY  181  GGAAAGTTCTTGCTGTGAACCGCCAAAAGGTGGCGATTCAATAAGCCTTGTTCAGCTGTC  240

ZFX  248  TCGTATTCACAGAATTTACACTTGTGCATTTTGTTGGCTCCTTTCTCCTTATGCACCATT  307
          || |||||||||||||||||||||||||||||||||| ||| || |||||||||||||||
ZFY  241  TCATATTCACAGAATTTACACTTGTGCATTTTGTTGGTTCCCTTTTCCTTATGCACCATT  300

ZFX  308  TTGTGCGTAAACAAAGCCCCAGCATGAGAGAAATGCTTCCCACACTCGTCGCATTCAATG  367
          ||||| ||||||||||||||||  ||||||||||||||||||||||| |||||||| || 
ZFY  301  TTGTGTGTAAACAAAGCCCCAGAGTGAGAGAAATGCTTCCCACACTCCTCGCATTCGATA  360

ZFX  368  GCCTTCTCTGCTTTGCTGGTCAGCTTGTGGCTCTCCATGTGA  409
          | ||| ||||| |||||||| |||||||||||||||||||||
ZFY  361  GACTTTTCTGCCTTGCTGGTTAGCTTGTGGCTCTCCATGTGA  402
```

Primer   *TaqI* recognition site

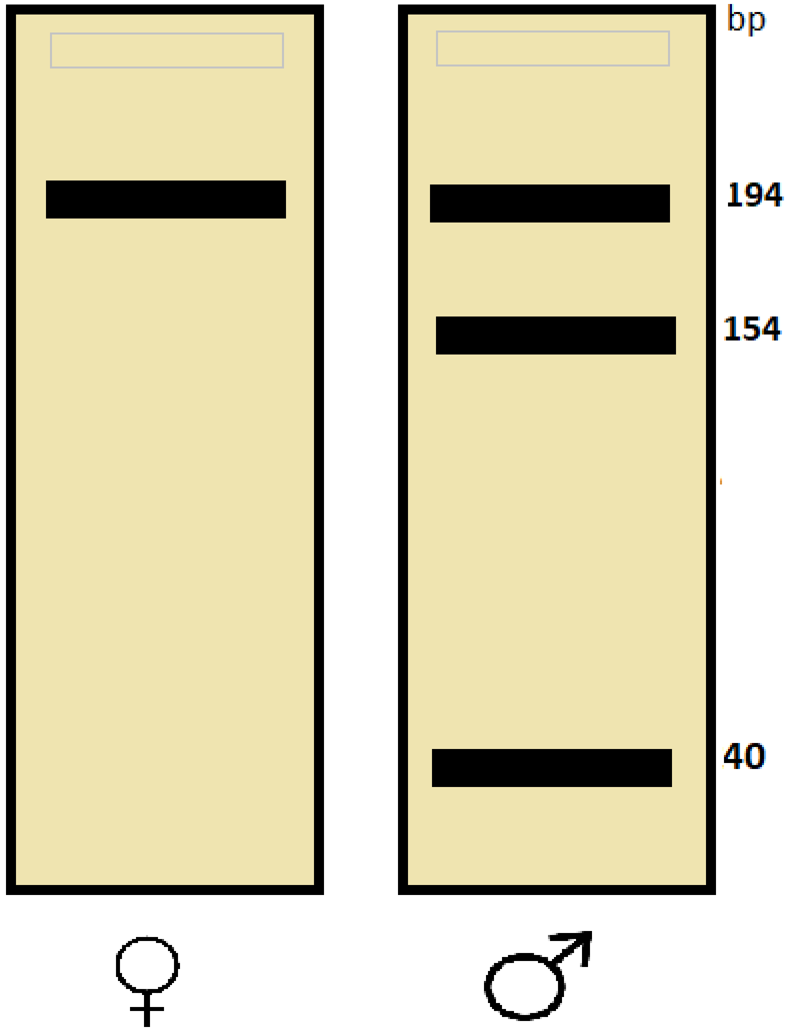

EMBL acc. No.:AY310919, AY310920

Domestic dog (*Canis lupus familiaris*). *ZFX, ZFY*

Fernando and Melnick, 2001

F:5'-ATAATCACATGGAGAGCCACAAGCT-3'
R:5'-GCACTTCTTTGGTATCTGAGAAAGT-3'

*Mse*I recognition site: 5'...T▼TAA...3' / 3'...AAT▲T...5'

```
ZFX 1588   GTTTACACAACCACCTGGAGAGCCACAAGCTGACCAGCAAAGCAGAGAAGGCCATTGAAT 1647
ZFY 654508 GTTTACACAACCACCTGGAGAGCCACAAGCTAACAAGCAAGGCAGAAAAGTCTATCGAAT 654449

ZFX 1648   GCGACGAGTGTGGGAAGCATTTCTCTCATGCTGGGGCTTTGTTTACGCACAAAATGGTGC 1707
ZFY 654448 GCGAGGAGTGTGGGAAGCATTTCTCTCACGCTGGGGCTTTGTTTACACACAAAATGGTGC 654389

ZFX 1708   ATAAGGAGAAAGGAGCCAACAAAATGCACAAGTGTAAATTCTGTGAATACGAGACAGCTG 1767
ZFY 654388 ATAAGGAAAAGGGAACCAACAAAATGCACAAGTGTAAATTCTGTGAATATGAGACAGCTG 654329

ZFX 1768   AACAAGGGTTATTGAATCGCCACCTTTTGGCGGTCCACAGCAAGAACTTTCCTCATATTT 1827
ZFY 654328 AACAAGGCTTATTGAATCGCCACCTTTTGGCGGTTCACAGCAAGAACTTTCCTCATATTT 654269

ZFX 1828   GTGTGGAGTGCGGCAAAGGTTTTCGTCACCCATCAGAGCTCAAAAAGCACATGCGAATCC 1887
ZFY 654268 GTGTGGAGTGTGGTAAAGGTTTTCGTCACCCGTCAGAGCTCAAAAAGCACATGCGAATCC 654209

ZFX 1888   ACACTGGGGAGAAGCCGTACCAGTGCCAGTACTGCGAATATAGGTCTGCAGACTCTTCTA 1947
ZFY 654208 ACACTGGCGAGAAGCCATACCAGTGCCAATACTGCGAATATAGGTCTGCAGACTCTTCTA 654149

ZFX 1948   ACTTGAAAACACATGTAAAAACTAAGCATAGTAAAGAGATGCCATTCAAGTGTGACATCT 2007
ZFY 654148 ACTTAAAAACACATGTGAAAACCAAGCATAGTAAAGAGATGCCATTCAAGTGTGACATCT 654089

ZFX 2008   GTCTTCTGACTTTCTCAGATACCAAAGAGGTGCAGCAACATGCTCTTATCCACCAAGAAA 2067
ZFY 654088 GTCTTCTGACTTTCTCAGATACCAAAGAGGTGCAGCAACATGCTGTCATCCACCAAGAAA 654029
```

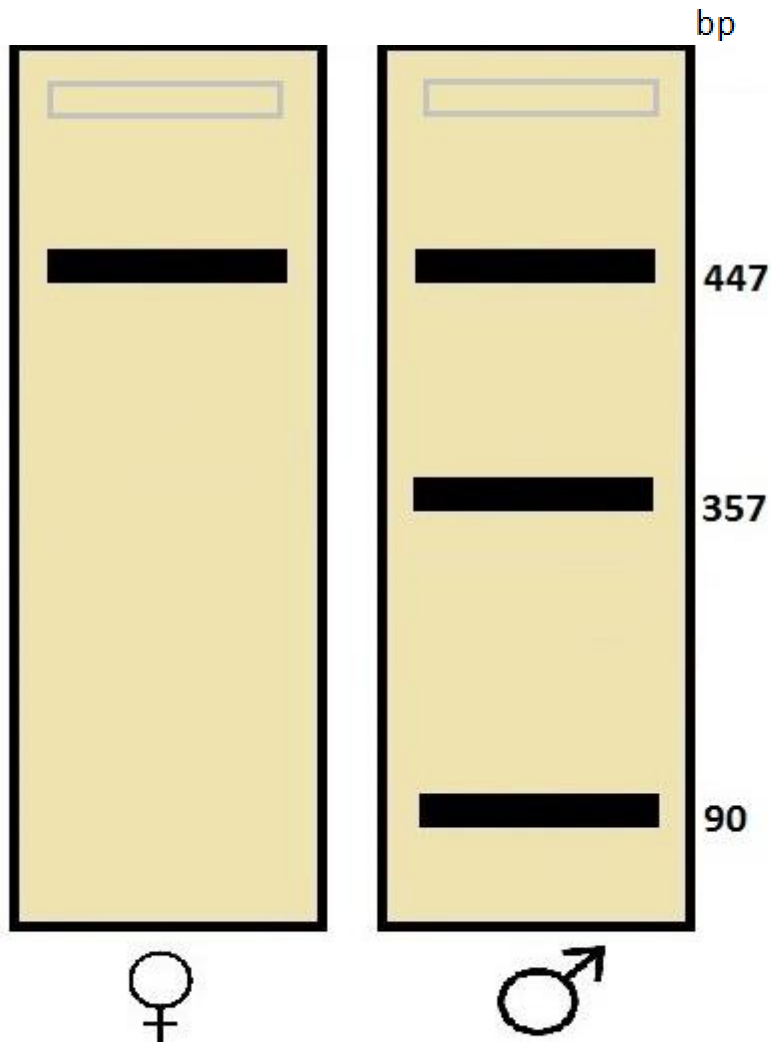

Primers | Mismatch to primers | *Mse*I recognition site | Restriction site ▲ | SNP

EMBL acc. No.: XM_014111639.2, KP081776.1

Wolf (*Canis lupus*), Domestic dog (*Canis familiaris*). AHTx40 marker, *DDX3Y*

Sastre et al., 2009

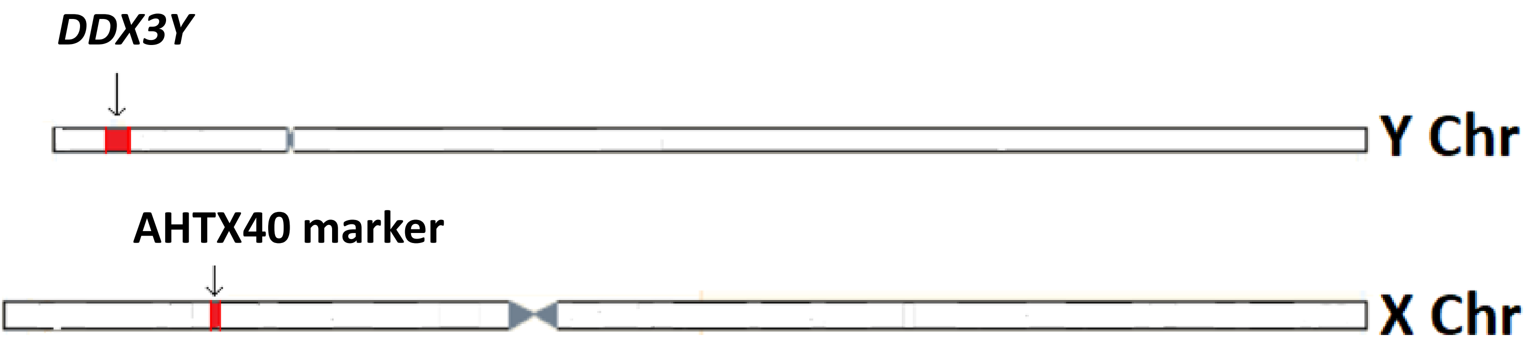

AHTX40 marker:

F:5′-GTAGCCCATTTGTTTATATTTGC-3′

R:5′-AAAACTGGACAGCCACATGC-3′

R:5′-GATCACTGTCTTACACCACAGGC-3′

DDX3Y:

F:5′-GCAAATTTGGTTTGTAGTCACA-3′

F:5′-TTGGGGGTGGTTTTATTGTC-3′

R:5′-CCATCTCAACATCGCTGAAC-3′

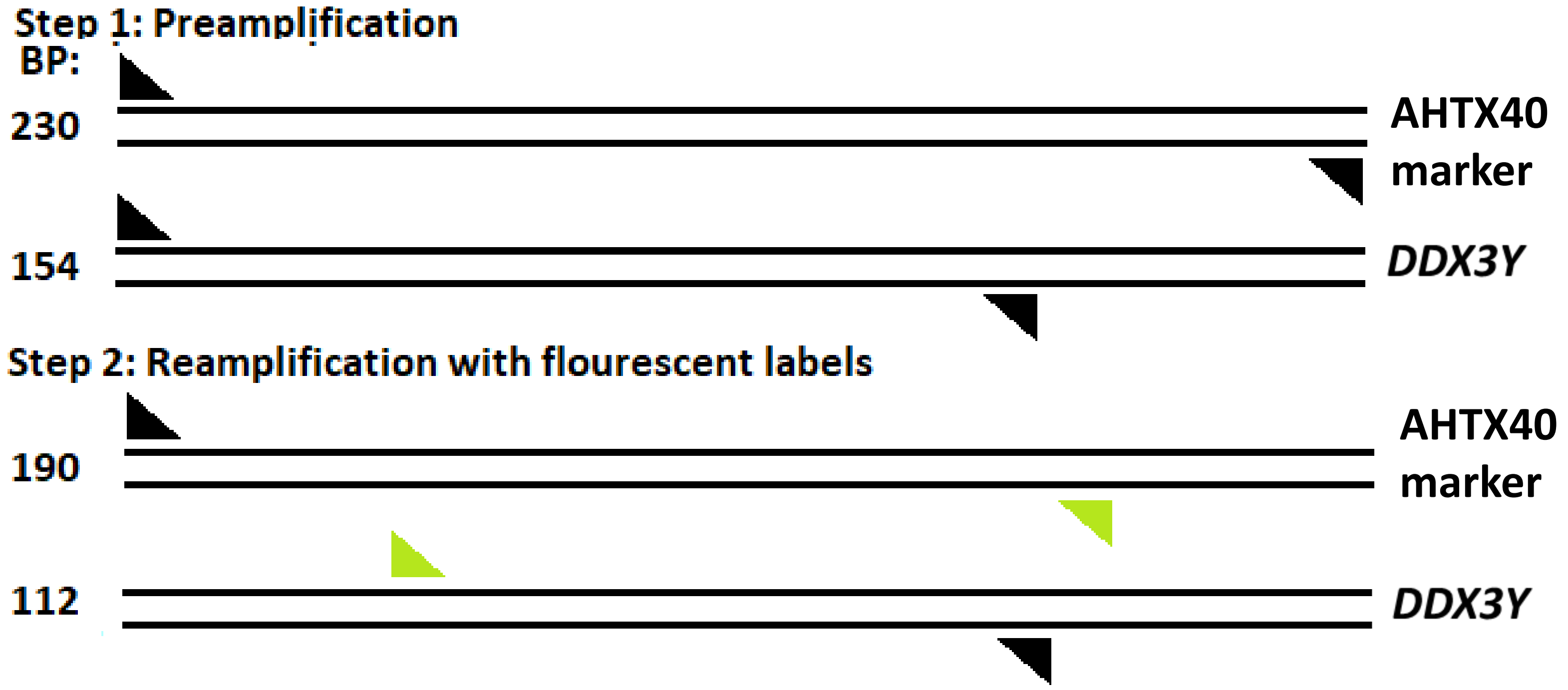

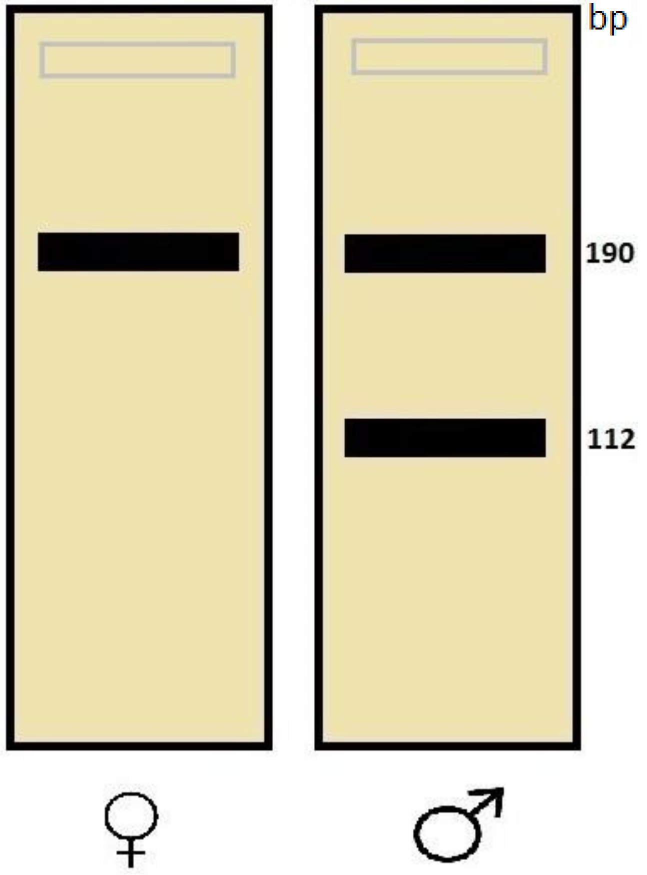

Coyote (*Canis latrans*). *ZFX, ZFY.*

Ortega et al., 2004

```
ZFX  1      ATAATCACATGGAGAGCCACAAGCTGACCAGCAAAGCAGAGAAGGCCATTGAATGCGACG  60
            ||||||||||||||||||||||||| |||||||| ||||| ||| | ||||||||||| |
ZFY  1      ATAATCACATGGAGAGCCACAAGCTAACCAGCAAGGCAGAAAAGTCTATTGAATGCGAGG  60

ZFX  61     AGTGTGGGAAGCATTTCTCTCATGCTGGGGCTTTGTTTACGCACAAAATGGTGCATAAGG  120
            |||||||||||||||||||||| ||||||||||||||||| |||||||||||||||||||
ZFY  61     AGTGTGGGAAGCATTTCTCTCACGCTGGGGCTTTGTTTACACACAAAATGGTGCATAAGG  120

ZFX  121    AGAAAGGAGCCAACAAAATGCACAAGTGTAAATTCTGTGAATACGAGACAGCTGAACAAG  180
            | || ||| |||||||||||||||||||||||||||||||||| ||||||||||||||||
ZFY  121    AAAAGGGAACCAACAAAATGCACAAGTGTAAATTCTGTGAATATGAGACAGCTGAACAAG  180

ZFX  181    GGTTATTGAATCGCCACCTTTTGGCGGTCCACAGCAAGAACTTTCCTCATATTTGTGTGG  240
            |||||||||||||||||||||||||||| |||||||||||||||||||||||||||||||
ZFY  181    GGTTATTGAATCGCCACCTTTTGGCGGTTCACAGCAAGAACTTTCCTCATATTTGTGTGG  240

ZFX  241    AGTGCGGCAAAGGTTTTCGTCACCCATCAGAGCTCAAAAAGCACATGCGAATCCACACTG  300
            |||| || ||||||||||||||||| ||||||||||||||||||||||||||||||||||
ZFY  241    AGTGTGGTAAAGGTTTTCGTCACCCGTCAGAGCTCAAAAAGCACATGCGAATCCACACTG  300

ZFX  301    GGGAGAAGCCGTACCAGTGCCAGTACTGCGAATATAGGTCTGCAGACTCTTCTAACTTGA  360
            |||||||||| ||||||||||| ||||||||||||||||||||||||||||||||||| |
ZFY  301    GGGAGAAGCCATACCAGTGCCAATACTGCGAATATAGGTCTGCAGACTCTTCTAACTTAA  360

ZFX  361    AAACACATGTAAAAACTAAGCATAGTAAAGAGATGCCATTCAAGTGTGACATCTGTCTTC  420
            |||||||||| ||||| |||||||||||||||||||||||||||||||||||||||||||
ZFY  361    AAACACATGTGAAAACCAAGCATAGTAAAGAGATGCCATTCAAGTGTGACATCTGTCTTC  420

ZFX  421    TGACTTTCTCAGATACCAAAGAAGTGC  447
            |||||||||||||||||||||||||||
ZFY  421    TGACTTTCTCAGATACCAAAGAAGTGC  447
```

Primers

EMBL acc. No.: AY145847.1, AY145848.1

F: 5′-CAAAAGGTGGCGATTCAATAA-3′

R: 5′-ATGGAGAGCCACAAGCTRAC-3′

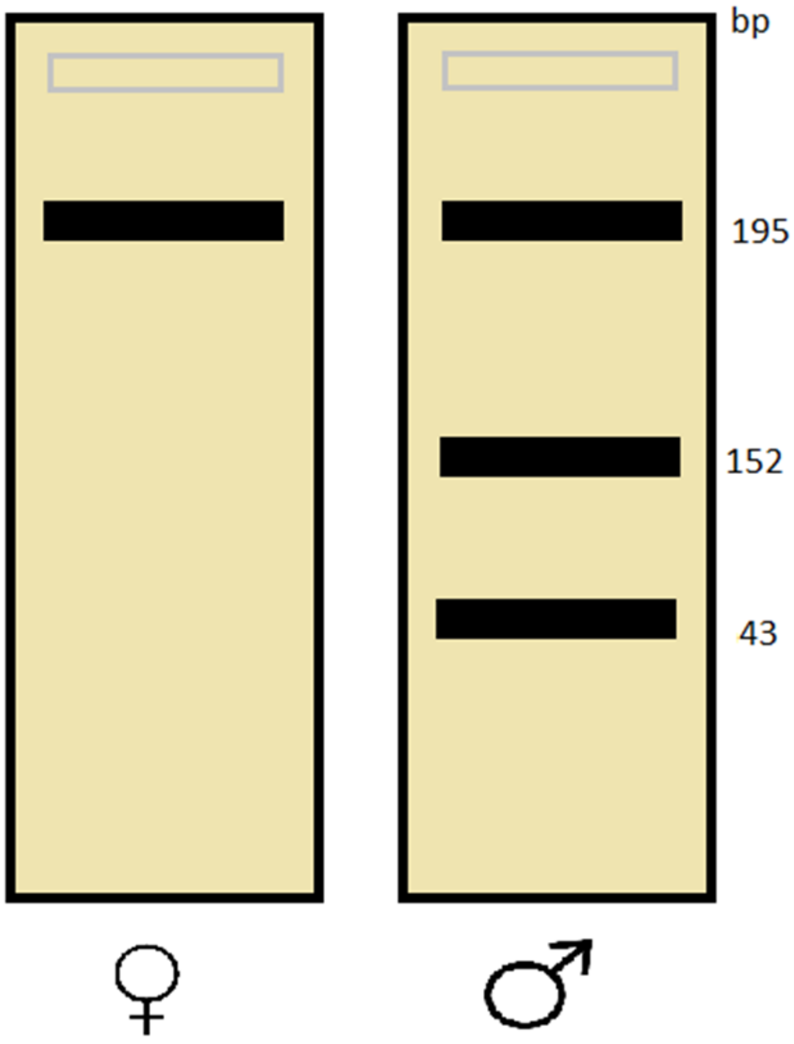

Band length estimated based on the agarose gel.

San Joaquin kit fox (*Vulpes macrotis mutica*). *ZFX, ZFY*

Ortega et al., 2004

F: 5′-CAAAAGGTGGCGATTCAATAA-3′

R: 5′-ATGGAGAGCCACAAGCTRAC-3′

*Taq*I recognition site: 5′...T▼CGA...3′ / 3′...AGC▲T...5′

```
ZFX 188  GGAAAGTTCTTGCTGTGGACCGCCAAAAGGTGGCGATTCAATAACCCTTGTTCAGCTGTC  247
         ||||||||||||||||| |||||||||||||||||||||||||| |||||||||||||||
ZFY 181  GGAAAGTTCTTGCTGTGAACCGCCAAAAGGTGGCGATTCAATAAGCCTTGTTCAGCTGTC  240

ZFX 248  TCGTATTCACAGAATTTACACTTGTGCATTTTGTTGGCTCCTTTCTCCTTATGCACCATT  307
         || |||||||||||||||||||||||||||||||||| ||| || |||||||||||||||
ZFY 241  TCATATTCACAGAATTTACACTTGTGCATTTTGTTGGTTCCCTTTTCCTTATGCACCATT  300

ZFX 308  TTGTGCGTAAACAAAGCCCCAGCATGAGAGAAATGCTTCCCACACTCGTCGCATTCAATG  367
         ||||| ||||||||||||||||  ||||||||||||||||||||||| |||||||| || 
ZFY 301  TTGTGTGTAAACAAAGCCCCAGAGTGAGAGAAATGCTTCCCACACTCCTCGCATTCGATA  360
                                                                ▲
ZFX 368  GCCTTCTCTGCTTTGCTGGTCAGCTTGTGGCTCTCCATGTGA  409
         | ||| ||||| |||||||| |||||||||||||||||||||
ZFY 361  GACTTTTCTGCCTTGCTGGTTAGCTTGTGGCTCTCCATGTGA  402
```

Primers Enzyme recognition site Restriction site ▲

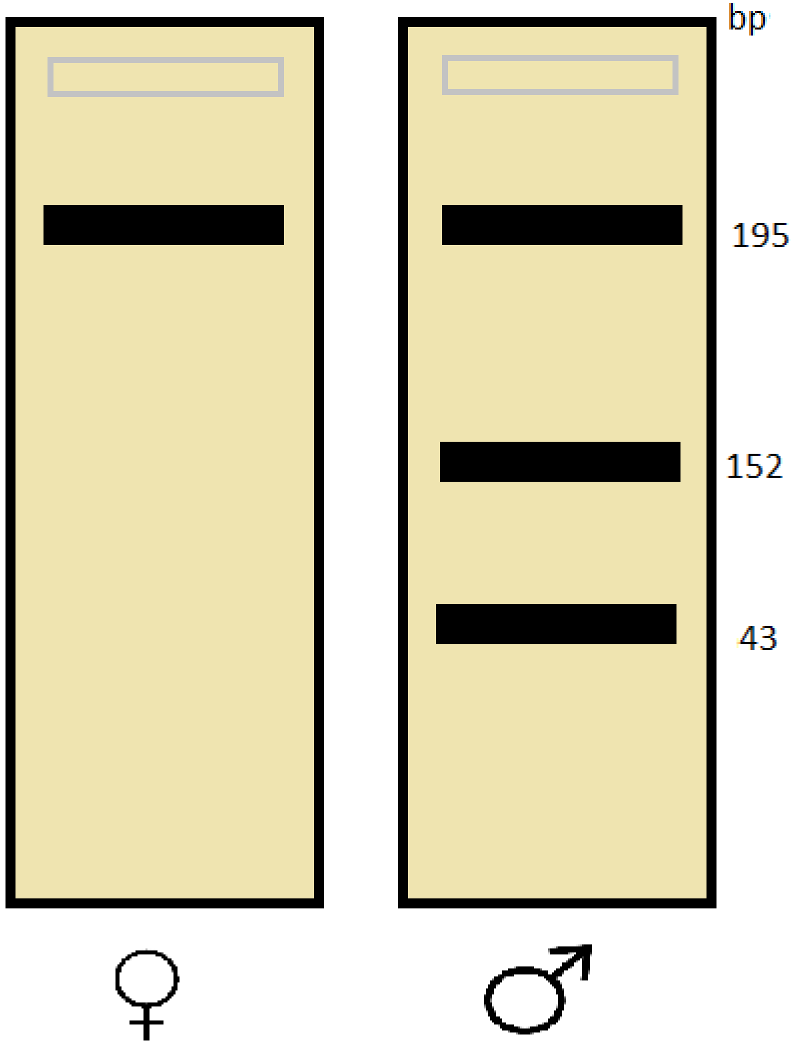

EMBL acc. No.: AY10919, AY310920

Brown bear (*Ursus arctos*), polar bear (*Ursus maritimus*), black bear (*Ursus americanus*), Asian black bear (*Ursus thibetanus*), sloth bear (*Melursus ursinus*), sun bear (*Helarctos malayanus*). *SMYC, ZFX,* 318.2

Bidon et al, 2013

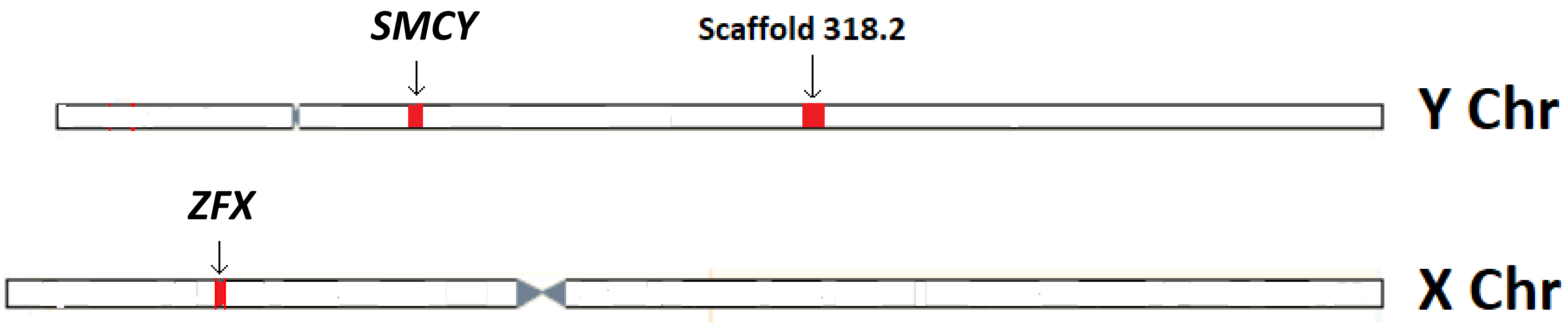

318.2
F:5′-AAGAAAAGTCATGCAACAGATACAG-3′
R:5′-TGATGCTTTGTGATCCTAATGTG-3′
SMCY
F:5′-GTCTTCCTCCTTAGAGGGTAATTAGG-3′
R:5′-TTCGTTTGATAATGGCCTAAAACTG-3′
ZFX
F:5′-AAAGAAATCCCTCAAACACGTTAC-3′
R:5′-TCGCCACCCRCAAATAG-3′

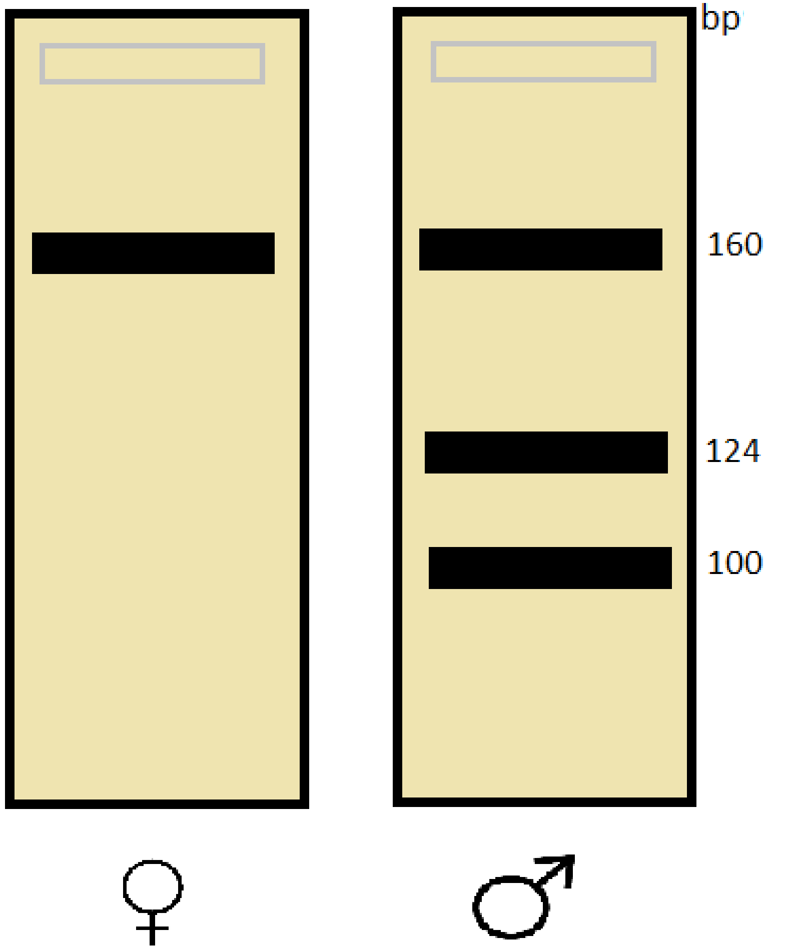

Giant panda *(Ailuropoda melanoleuca)*. *ZFX, ZFY*

Durnin et al., 2007

PS1
F:5′-CATCCTGAACACCTTACCAAGAAGA-3′
R:5′-AGCATGAGAGAAATGCTTCCCAC-3′
R:5′-CTTGTGCATTTTGTTGGCTCCTTTT-3′

PS2
F:5′-GAAGATAAGTTTACACAACCA-3′
R:5′-CTTGTGCATTTTGTTGGCTCC-3′
R:5′-CATTTTGTGAGTAAACAAAGCT-3′

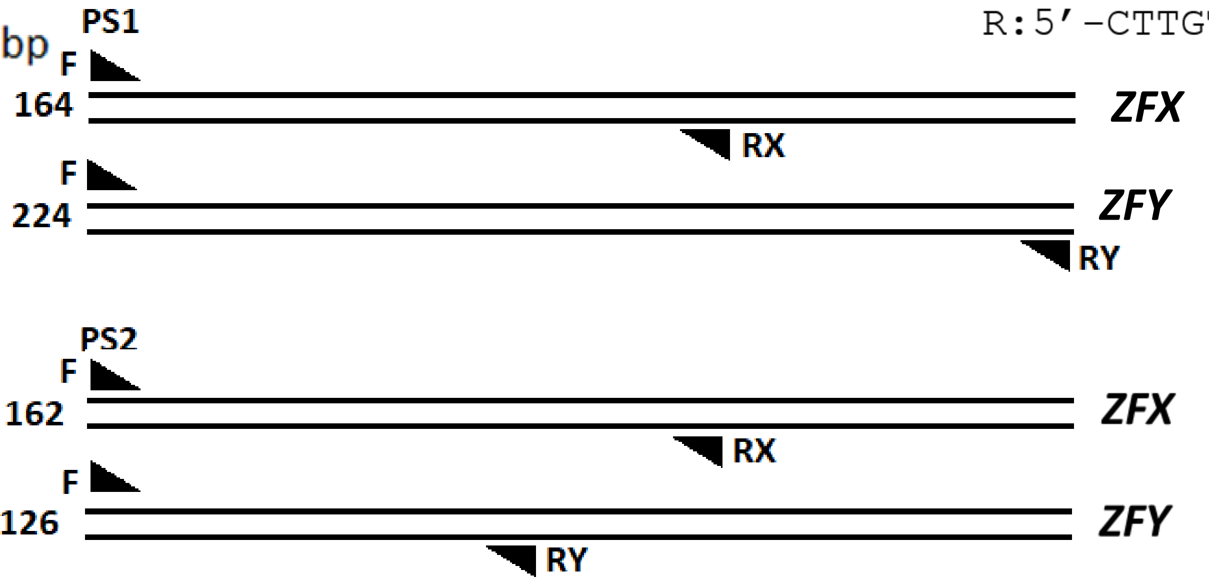

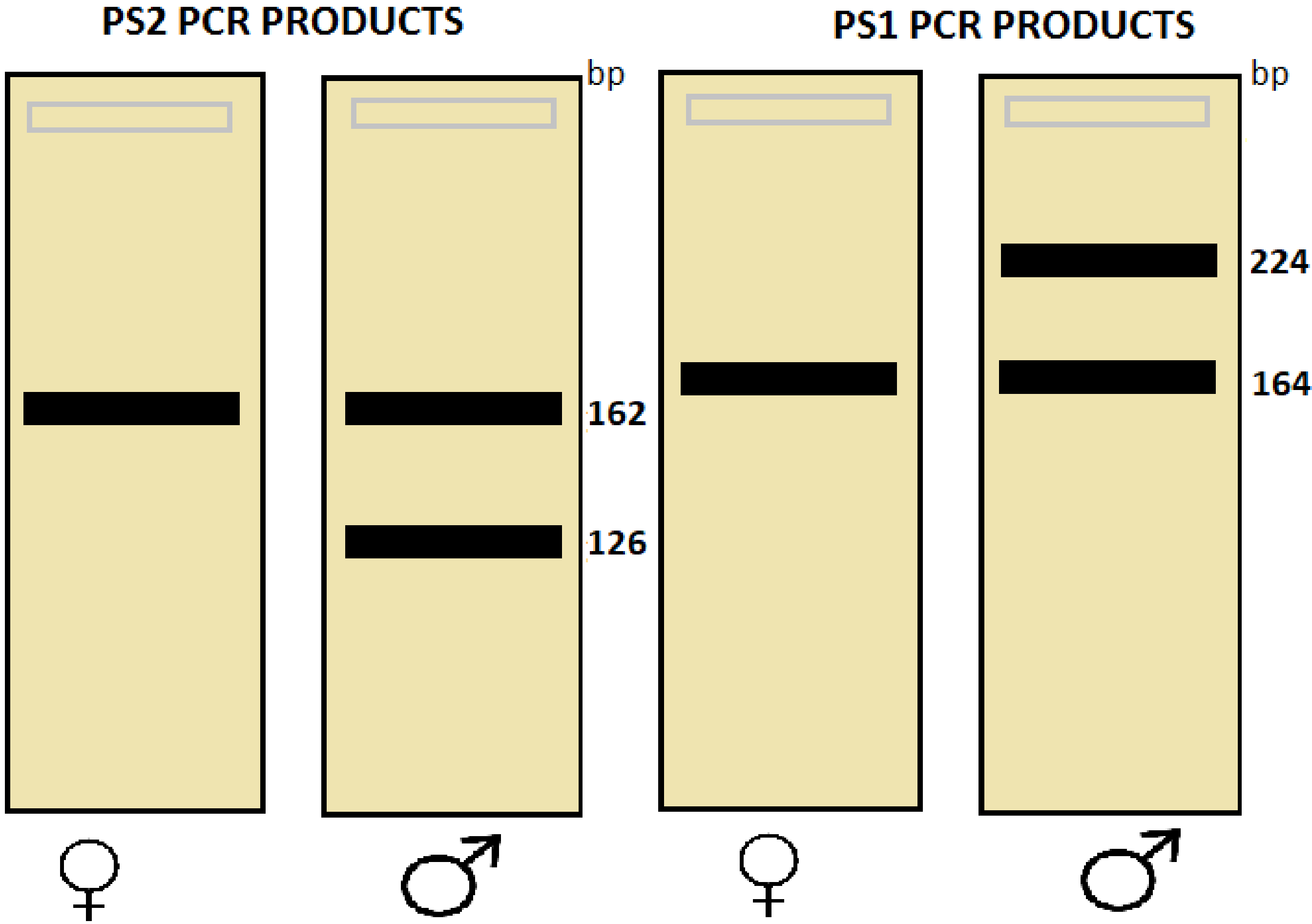

Red panda (*Ailurus fulgens*). *AMELX, AMELY*

Kumar et al., 2015

```
AMELX 10   TCCCCATGATGCCAGCTCAGCAGCCCGTGGTCCCCAGCAGCCAATGATGCCAGTTCCCG   69
           |||||||||||||||||||||||||| |||||||||||||||||||||||||||||||||
AMELY 1    TCCCCATGATGCCAGCTCAGCAGCCTGTGGTCCCCAGCAGCCAATGATGCCAGTTCCCG   60

AMELX 70   GCCAACACTCCATGACTCCAACCCAACACCACCAGCCAAACCTCCCCCTGCCTGCCCAGC   129
           ||||||||||||||||||||||||||||||||||||||||||||||||||||||||||||
AMELY 61   GCCAACACTCCATGACTCCAACCCAACACCACCAGCCAAACCTCCCCCTGCCTGCCCAGC   120

AMELX 130  AGCCCTTCCAGCCCCAGCCGGTCCAGCCCCAGCCTCACCAGCCCATCCAGCCCATCCAGC   189
           |||||||||||||||||| |||||||||||||||||||||||||||||||||||||||||
AMELY 121  AGCCCTTCCAGCCCCAGCAGGTCCAGCCCCAGCCTCACCAGCCCATCCAGCCCATCCAGC   180

AMELX 190  CCATCCAGCCATCCGACCCATCCGGCCCATCCAGCCACCCCTGCACCCCATCCCGCCCC   249
           ||||||||||||||| || | ||  |       |       ||||||||||||||||||
AMELY 181  CCATCCAGCCATCCGGCC-A-CC--C-------G-------TGCACCCCATCCCGCCCC   222
                             * *  **  ******* *******

AMELX 250  TGCCGCCGCAGCCACCTCTGCCTCCGATGTTCCCATACAGCCCCTGCCCCCCATGCTTC   309
           |||||||||||||||||||||||||||||||||||||| |||||||||||||||||||||
AMELY 223  TGCCGCCGCAGCCACCTCTGCCTCCGATGTTCCCATACCGCCCCTGCCCCCCATGCTTC   282

AMELX 310  CTGACCTGCCTCTGGAAGCCTGGCCAGCAACAGACAAGACCAAGCGGGAACAA   362
           ||||||||||||||||||||||||| ||||||||| |||||||||||||||||
AMELY 283  CTGACCTGCCTCTGGAAGCCTGGCCGGCAACAGACGAGACCAAGCGGGAACAA   335
```

Primers SSIndel ******

F:5′-ACACTCCATGACTCCAACCC-3′

R:5′-CTGTATGGGGAACATCGGAG-3′

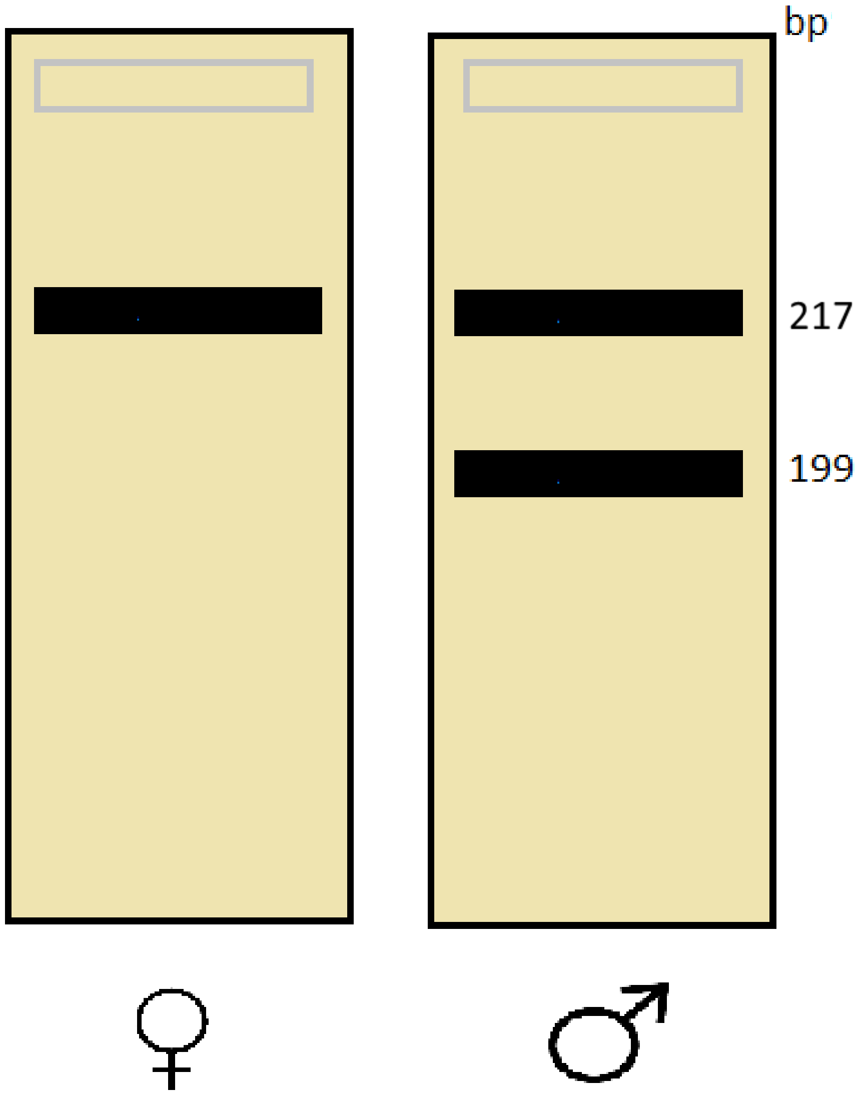

EMBL acc. No.: KM017653, KM017654

Raccoon (Procyon lotor). *ZFX, ZFY*

Okuyama et al., 2014

Universal primers: F:5′-ATAATCACATGGAGAGCCACAAGCT-3′
R:5′-GCACTTCTTTGGTATCTGAGAAAGT-3′

Primer set 1:
ZFX-IPfw:5′-AGCCGTACCAGTGCCAGTA-3′
ZFY-IPrw:5′-TGCAGACCTATACTCGCAGAAT-3′

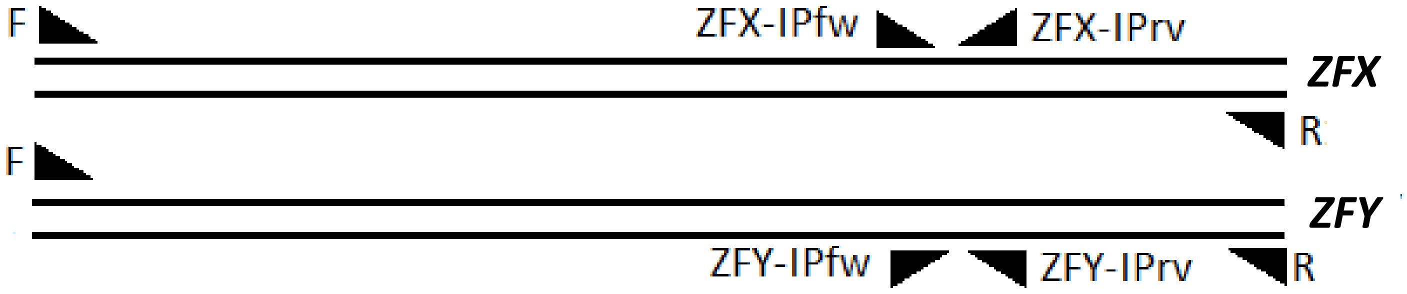

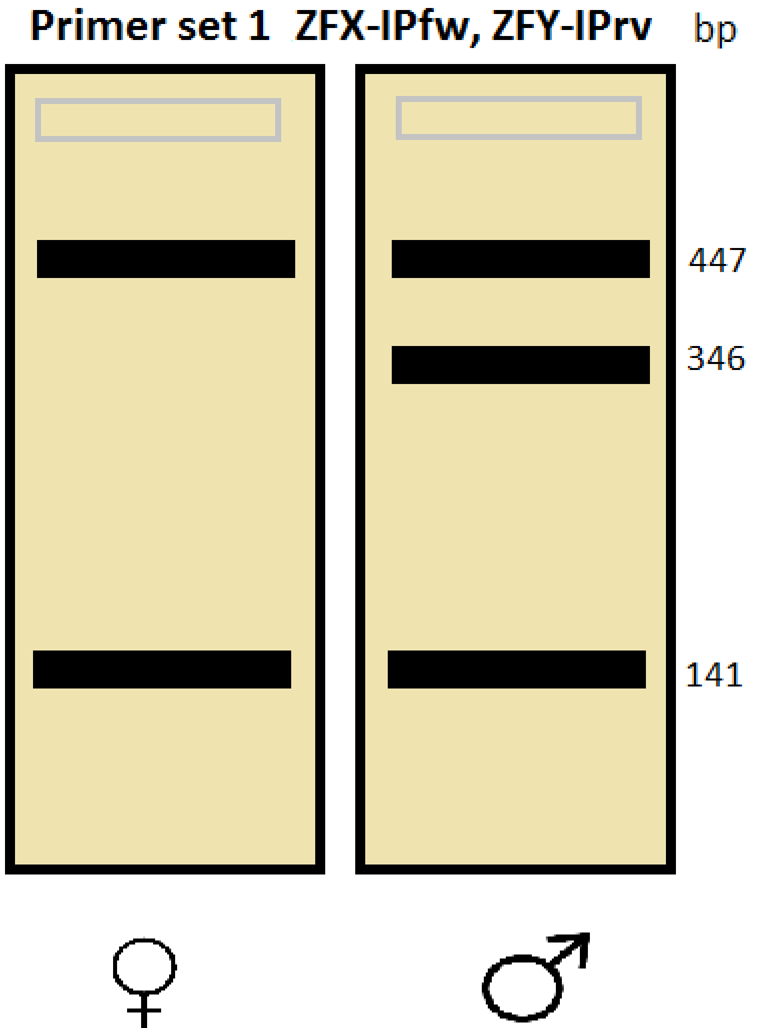

EMBL acc. No.: AB856034, AB856035

Raccoon (Procyon lotor). *ZFX, ZFY*

Okuyama et al., 2014

Universal primers:
F:5′-ATAATCACATGGAGAGCCACAAGCT-3′
R:5′-GCACTTCTTTGGTATCTGAGAAAGT-3′

Primer set 2:
ZFY-IPfw:5′-AGCCGTACCAGTGCCAATT-3′
ZFX-IPrw:5′-GCGGACCTATACTCGCAGTA-3′

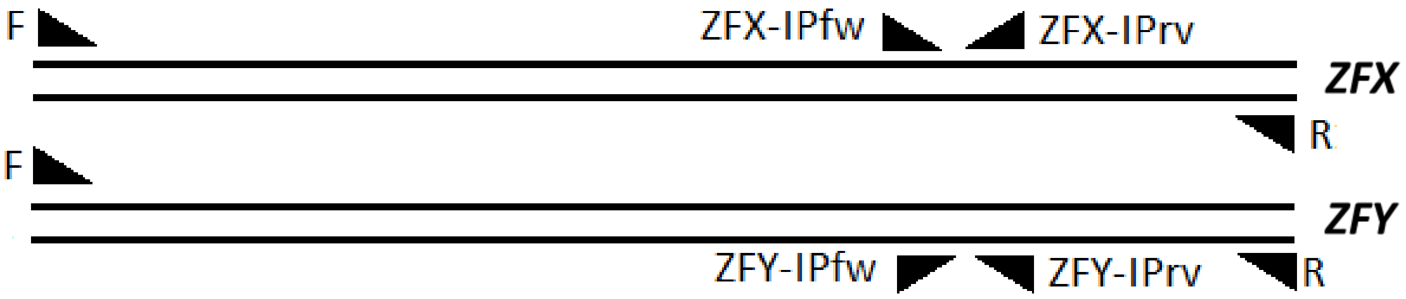

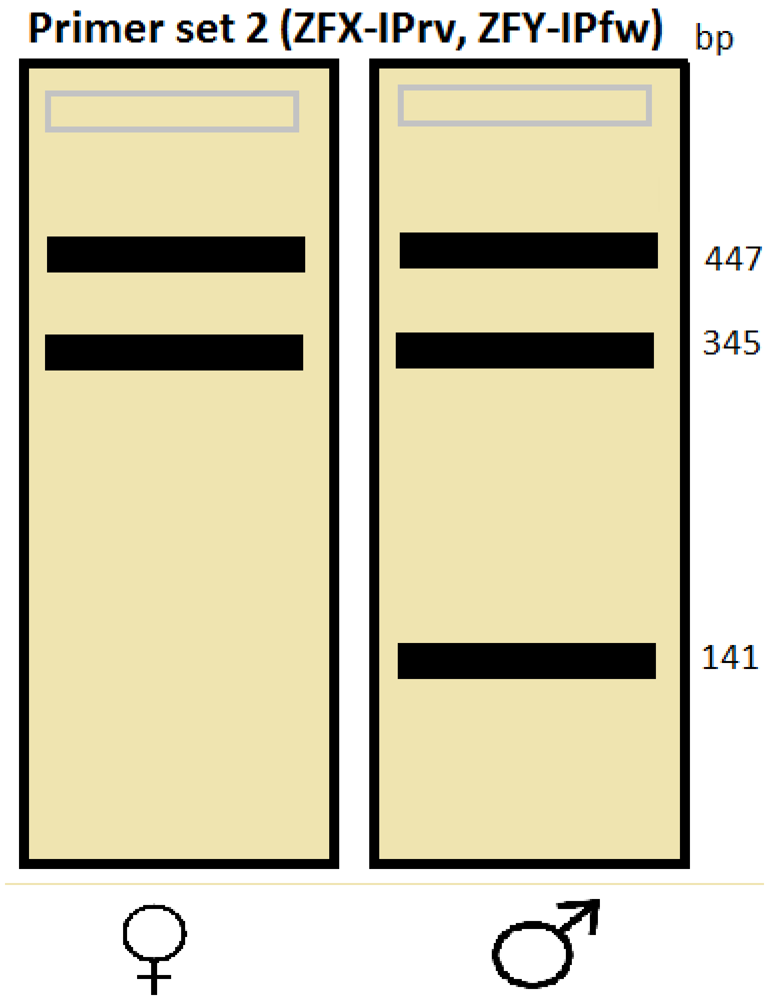

EMBL acc. No.: AB856034, AB856035

White-tailed deer (*Odocoileus virginianus*). *ZFX, ZFY*

Lindsay and Belant, 2007

```
ZFX        ------------------------------------------------------------
ZFY        GGTAAGGAGAAACCTTTAGCATGTCATGTGTGCTTTGTGTTTGCTTGTATCTAGCTCTCT
            *********** ************ ************ ************ ***********

ZFX        ------------------------------------------------------------
ZFY        GTTCGGGGGATTCTCTAGGCAAGAGTACTGGAGTTGGTTGCCATTTCCTTCTCCAGGTGA
            *********** ************ ************ ************ ***********

ZFX        -----------------------------------------------GGTAAGAGAAGAC
ZFY        TCTTCCCAACCCAGGGATCGAACCCGCATCTCCTGCCTCTCCTGCAGTGGCAGAGAGATT
           *********** ************ ************ ************

ZFX        CTTCAG-CATAGTATC---CATCCTGATCAAATACTTCACCCTATTTACTGTTTGGTGGT
ZFY        CTTTTCCTCTGCTGCACATGGAAGATCCCGATCAAACACTTTAATTACTTTCAGGTGTT
                 *         ***

ZFX        TTAGACAGTGAATATTTGTAATCTTTTCTGAAAATAACCTTTTAAGTGAGGATTCCAATA
ZFY        TTAGACTGAAAATACTTGTAATTGTTTCTGAAGATAACTTTTAAAACAGGTTT-TTATTG

ZFX        TAGC-----------AGTAGAAAAGTGTGCAAATGTAATAAGTAAAACAAGTGGAAATGA
ZFY        TATTAATACAAAGGTATTTAAAGAGCTAATTACTAACTGAGATGAAACAGGAGGAAATGA
               ************

ZFX        AGTTTTCACATAGATTTTGGAGCTGTCATCTTCCCTGATAGGTGGGACTGTAGACAT
ZFY        AGTTTTCACATAAATACTTGGAGTTGTCGTCTTCCCTGTTAGGTGGGACTGTTGACAT

SSIndel****** Primers not included in alignment
```

F:5′-GCTGACCCTGGAGAAGATGACTTA-3′

R:5′-TCATTCTCAGGCTCACTCTCCACA-3′

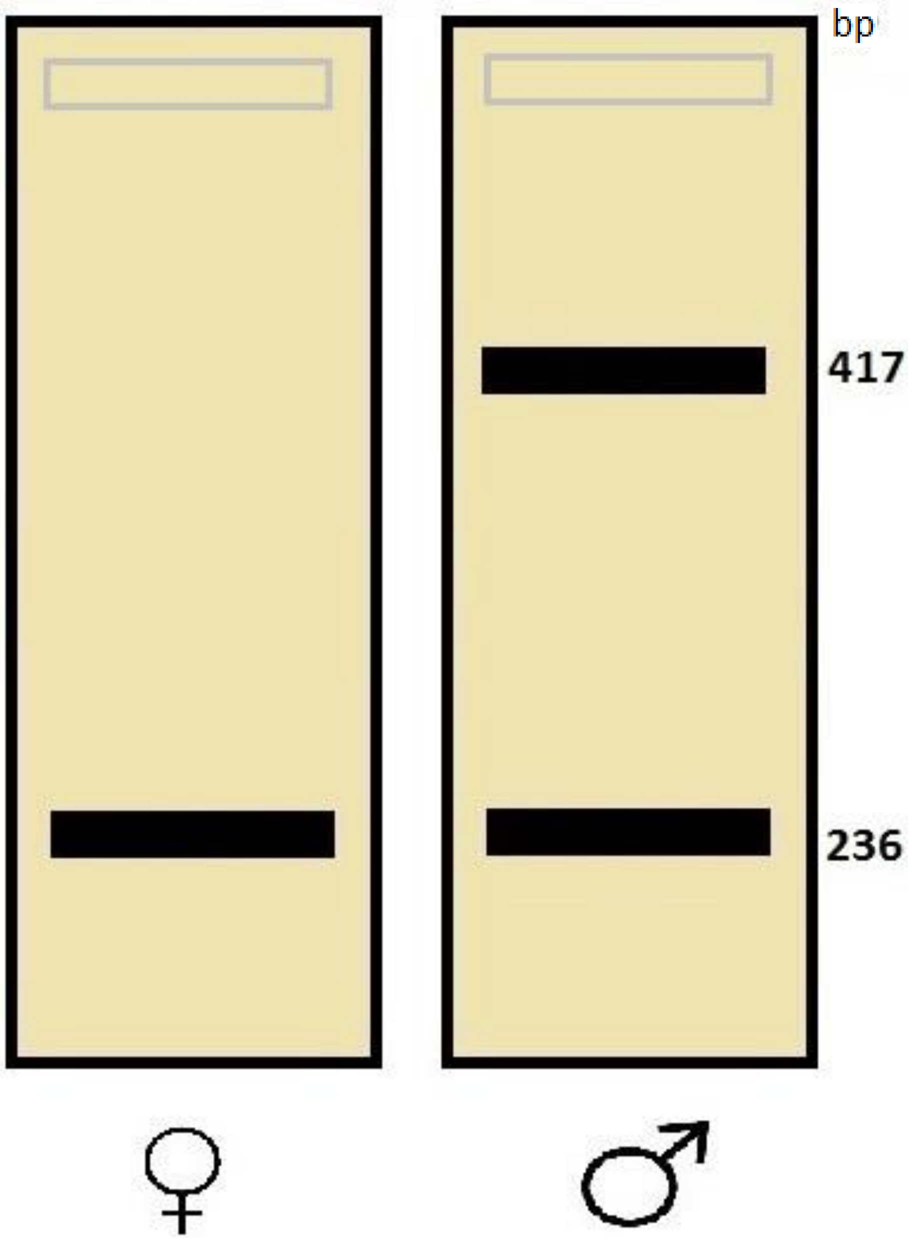

EMBL acc. No.: EF077165, EF077163

Cattle (*Bos taurus*). *AMELX, AMELY*

Gokulakrishnan et al., 2013

F:5′-CCGCCCAGCAGYCCTTCCAG-3′
R:5′-TGGGGAATATYGGAGGCAGAGG-3′

```
AMELX      CAGCCAAACCTCCCTCTGCCCGCCCAGCAGCCCTTCCAGCCCCAGTCCATCCAGCCGCAG 60
AMELY      ---------------------------------------------------------CAG 3
                                                 ******************

AMELX      CCTCACCAGCCCCTGCAGCCTCACCAGCCCCTGCAGCCCATGCAGCCCATGCAGCCCTTG 120
AMELY      CCAAACCTCCCTCTGCCCGCCCAGCAGTCCTTCCAGC------CCCAGCCCATCCAGCCA 57
                                                ******

AMELX      CAGCCCCTGCAGCCCCTGCAGCCCCAGCCACCTGTGCACCCCATCCAGCCCTTGCCGCCA 180
AMELY      CAGCCTCACCAACCCCTGCAGCCCCAGCCACCTGTGCACCCCATCCAGCGCTTGCCACCA 117

AMELX      CAGCCACCTCTGCCTCCGATATTCCCCATGCAGCCTCTGCCCCCCATGCTTCCTGACCTG 240
AMELY      CAGCCACCTCTGCCTCCAATATTCCCCATGCAACCTCTGCCCCCTGTGCTTCCTGACCTG 177
Primers  SSIndel  *****
```

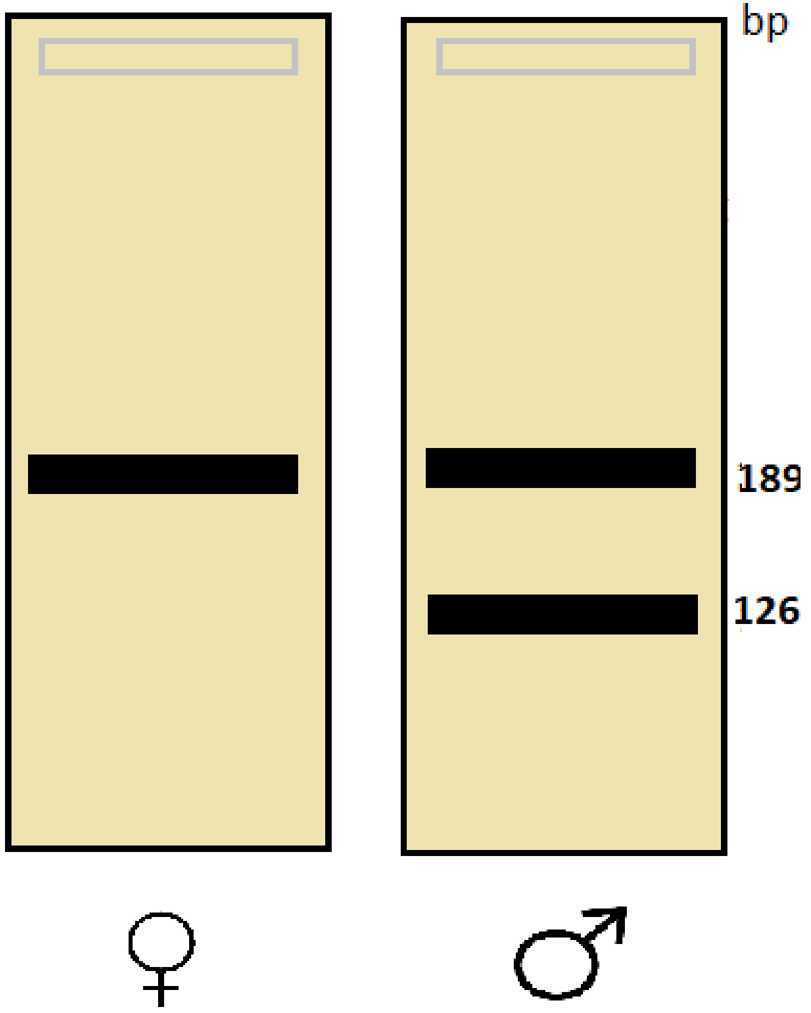

ENSBTAG00000012089, EMBL acc. No.: AC216937.5

Cattle (*Bos taurus*). *AMELX, AMELY*

Chen et al., 1999

F: 5′-AAATTCTCTCACAGTCCAAG-3′
R: 5′-CAACAGGTAATTTTCCTTTAG-3′

```
AMELX      CCCAGAGATTGAAAATCTCTCACAGTCCAAGGCCTACAGTTTCAGTAGCTACAGCTACAG
AMELY      CACAGAAATCAAAATTCTCTCACAGTCCAAGACCTAGAGTTTCAACTGCA----------
                                                             **********

AMELX      CTCAGTGATTCAGTTAGCCCAAGTGTGTGCTGTAAGTTTATATTATAAAGAGTTCATCTA
AMELY      GTTAGTGATTCTATTATTCCAAGTGTGTGTTGTAAGTTTATAAATGAGCTTGTTTATCTA

AMELX      TATGGCTTAGAATTAACACAATAGCTTTTATGGTGGACAGGGAGGCCTGGCGTGCTGCGA
AMELY      TATGGCATACACTTTGTGAATTCAAAATCTACAATTGTTTGGAATTTTAA--TGG----C
                                                             **   ****

AMELX      TTCATGGGGTCGTAAAGAGTCGGA--CATGACTGAGCTACTGATCTGATCTGATCTGAAA
AMELY      AATATGAATTGCTATTGAAATGCAGTATAATAAAGGCAACAATTCTTATCTTCTGGTTGC

AMELX      TTCTATAATTACTTTGAATTCCTACCAGAATATCTATTATTGAAACCTGTTAATTTTACA
AMELY      TTAACTAGTACATT-AGAC----------------------------------TTATACA
                              **********************************

AMELX      GACAGTACTATAAGGGAACATTAGTTCTCTTTATCTTTCAAAGGTTTGACCAGCAATAAT
AMELY      GAAATAAATA--------------------------------------------------
                     **************************************************

AMELX      AGGCTAGAATTGAACCGGAGATTTCTTTTAAAAGGGTAATTTTTGCCAGTGAGGGGATGC
AMELY      -----------------------------AAAA-GAGTATTTCTGGGATTGAGGAGAAGG
           *****************************

AMELX      AGATATTTGAAC--AGGTAGGTGGGAGGTCTCTTTAAAAACCTAAAGGAAAATTACTTGT
AMELY      AAGTATCAGTTTTAAAAAATTATAGCTGGATCTTCAAGGCTCTAAAGGAATATTACCTGT

AMELX      TTAAAGTGGTTTTCAATCTAACTTGGAGGAAGCTAAACTACACAGATTGCTAGATTCCTT
AMELY      TGTAGGTGGTTTTAAAGT-AATTTGGAAACAGTTAAAGTAGACAA--ACGTAGATTCTAT
```

Primers Mismatch to primers SSIndel ******

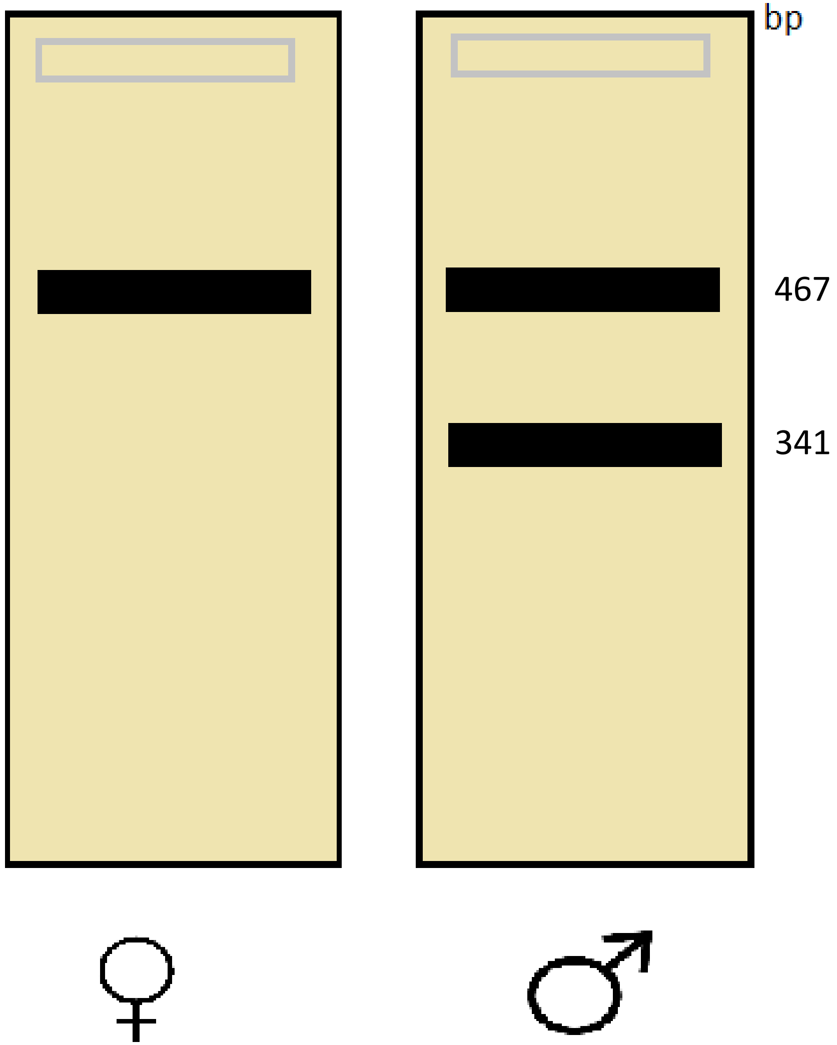

EMBL acc. No.: L48608.1, L48609.1

Cattle (*Bos taurus*). *DDX3X*, *DDX3Y*

Gokulakrishnan et al., 2012

F: 5‘-AGGAAGCCAGGAAAGTAA-3‘

R: 3‘-CATCCACGTTCTAAGTCTC-5‘

```
DDX3X   1    GAAAATGGAAGATATGGGCGCCGCAAACAATACCCAATCTCTTTGGTGTTAGCACCAACT   60
             |||| |||||| ||||| ||||| ||||| || |||||||| ||||| ||||| ||||| 
DDX3Y   1    GAAAGTGGAAGGTATGGACGCCGTAAACAGTATCCAATCTCCTTGGTTTTAGCCCCAACA   60

DDX3X   61   AGAGAATTGGCCGTACAGATCTATGAGGAAGCCAGGAAAGTAAGTATGC--CTTTCAGGT   118
             ||||| ||||| |||||||||||||||||||||||||||||||||||||  ||| | | |
DDX3Y   61   AGAGAGTTGGCTGTACAGATCTATGAGGAAGCCAGGAAAGTAAGTATGCGACTTGCTGAT   120

DDX3X   119  TATTGGCCTTCTCATTGATTCTGATGAAGTGTATTATGACCATGTAAAAATCTTGGTCTT   178
              |||| | || | || ||||||  || | | | ||||||||||| |||   |         
DDX3Y   121  AATTGACTTTTAATAGATTCTAGTGTAATTT-TTATGACCATCTAA---T---------   167
                                                           ****  *************

DDX3X   179  AAATTTAATAAATTTTTCTTTGCTTATAGTTTTCATACCGATCTAGAGTTCGTCCTTGTG   238
             ||||| | |   |    ||| |    |||||||||||||| ||| |||||| ||||||||
DDX3Y   168  AAATT-A-T---TG---CTT-G----TAGTTTTCATACCGGTCTCGAGTTCTTCCTTGTG   214
                  *  *  ****  ****    *  ******

DDX3X   239  TGGTTTATGGTGGTGCTGATATTGGTCAGCAAATTCGAGACTTAGAACGTGGATGCCATT   298
             |||| || |||||||||||||||||||| |||||||||||||||||||||||||| || |
DDX3Y   215  TGGTATACGGTGGTGCTGATATTGGTCAACAAATTCGAGACTTAGAACGTGGATGTCACT   274

DDX3X   299  TATTAGTTGCGACTCCAGGACGTCTAGTGGATATGATGGAAAGAGGAAAAATTGGATTAG   358
             | || || || ||||||||||| || ||||||||||||||||| ||||| ||||| ||||
DDX3Y   275  TGTTGGTGGCCACTCCAGGACGCCTGGTGGATATGATGGAAAGGGGAAAGATTGGGTTAG   334

DDX3X   359  ACTTCTGCAA   368
             | ||||||||
DDX3Y   335  ATTTCTGCAA   344
```

Primer SSIndel ******

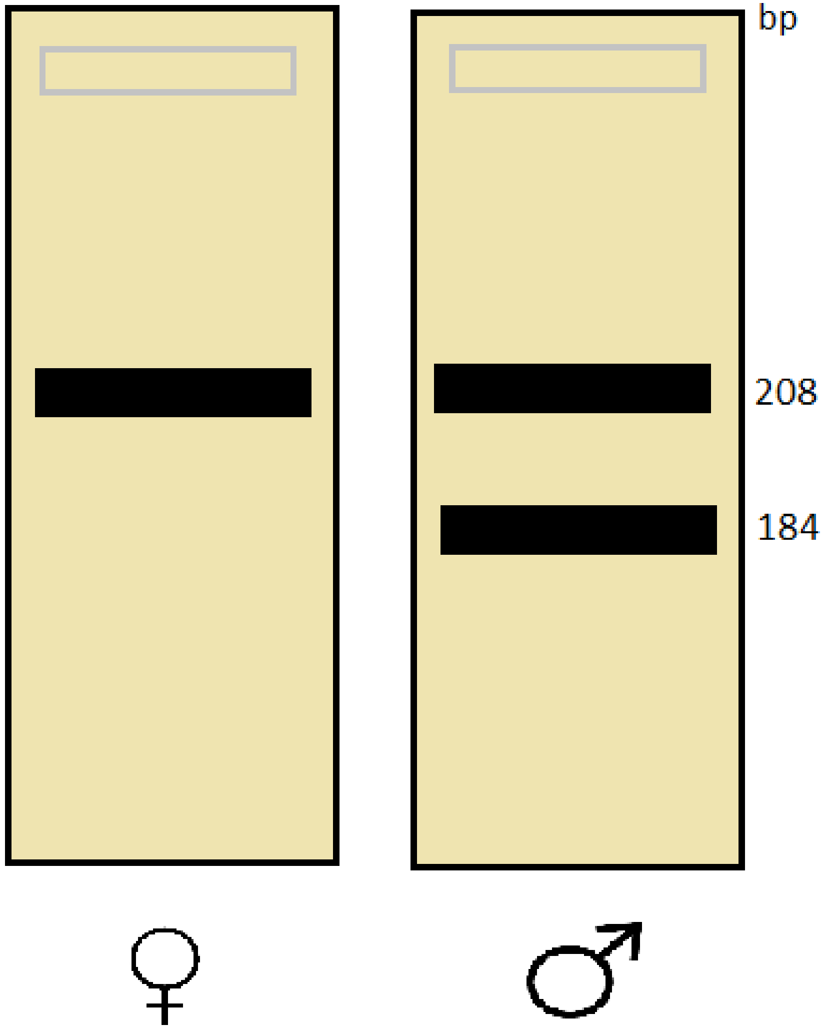

ENSBTAG00000035907, ENSBTAG00000045750.1

Cattle (*Bos taurus*). *DDX3X, DDX3Y*

Gokulakrishnan et al., 2012

```
DDX3X  1    GAAAATGGAAGATATGGGCGCCGCAAACAATACCCAATCTCTTTGGTGTTAGCACCAACT  60
            |||| |||||| ||||| ||||| ||||| || |||||||| ||||| ||||| ||||| 
DDX3Y  1    GAAAGTGGAAGGTATGGACGCCGTAAACAGTATCCAATCTCCTTGGTTTTAGCCCCAACA  60

DDX3X  61   AGAGAATTGGCCGTACAGATCTATGAGGAAGCCAGGAAAGTAAGTATGC--CTTTCAGGT  118
            ||||| ||||| |||||||||||||||||||||||||||||||||||||  ||| | | |
DDX3Y  61   AGAGAGTTGGCTGTACAGATCTATGAGGAAGCCAGGAAAGTAAGTATGCGACTTGCTGAT  120

DDX3X  119  TATTGGCCTTCTCATTGATTCTGATGAAGTGTATTATGACCATGTAAAAATCTTGGTCTT  178
             |||| | || | || ||||||  || | | | |||||||||| |||   |         
DDX3Y  121  AATTGACTTTTTAATAGATTCTAGTGTAATTT-TTATGACCATCTAA---T---------  167
                                                           *** **********

DDX3X  179  AAATTTAATAAATTTTTCTTTGCTTATAGTTTTCATACCGATCTAGAGTTCGTCCTTGTG  238
            ||||| | |   |    ||| |    |||||||||||||| ||| |||||| ||||||||
DDX3Y  168  AAATT-A-T---TG---CTT-G----TAGTTTTCATACCGGTCTCGAGTTCTTCCTTGTG  214
                 *  * ****  ***   *  ******

DDX3X  239  TGGTTTATGGTGGTGCTGATATTGGTCAGCAAATTCGAGACTTAGAACGTGGATGCCATT  298
            |||| || |||||||||||||||||||| |||||||||||||||||||||||||| || |
DDX3Y  215  TGGTATACGGTGGTGCTGATATTGGTCAACAAATTCGAGACTTAGAACGTGGATGTCACT  274

DDX3X  299  TATTAGTTGCGACTCCAGGACGTCTAGTGGATATGATGGAAAGAGGAAAAATTGGATTAG  358
            | || || || ||||||||||| || ||||||||||||||||| ||||| ||||| ||||
DDX3Y  275  TGTTGGTGGCCACTCCAGGACGCCTGGTGGATATGATGGAAAGGGGAAAGATTGGGTTAG  334

DDX3X  359  ACTTCTGCAA  368
            | ||||||||
DDX3Y  335  ATTTCTGCAA  344
```

Primer (TGAGGAAGCCAGGAAAGTAAGTAT, TTGTGTGGTTTATGGTGGTGC / TTGTGTGGTATACGGTGGTGC highlighted) SSIndel ******

F: 5'-TGAGGAAGCCAGGAAAGTAAGTAT-3'

R: 3'-GCACCACCRTAWCCACACAA-5'

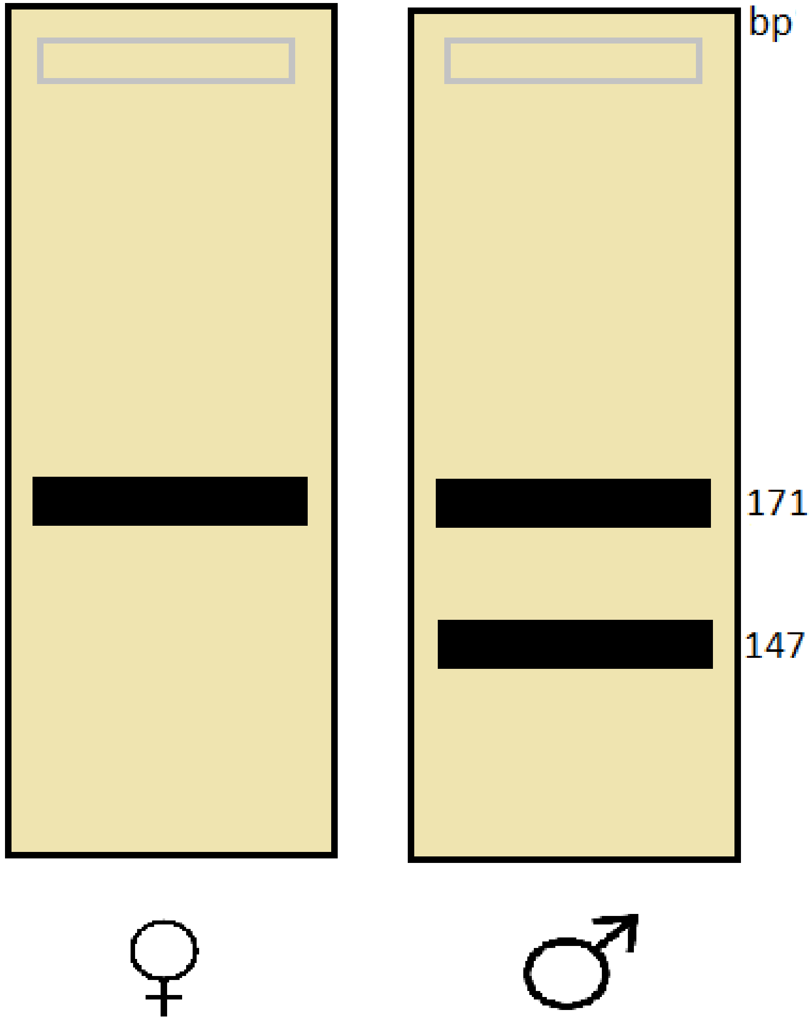

ENSBTAG00000035907, ENSBTAG00000045750.1

Cattle (*Bos taurus*). *ZFX, ZFY*

Aasen and Medrano, 1990

F:5'-ATAATCACATGGAGAGCCACAAGCT-3'
R:5'-GCACTTCTTTGGTATCTGAGAAAGT-3'

*Pst*I recognition site: 5'...CTGCA▼G...3' / 3'...G▲ACGTC...5'

```
ZFX 1402 TACCAACAAGAAGATAAGTTTACACAACCACCTGGAGAGCCACAAGCTTACCAGCAAGGC 1461
ZFY 1380 TACCAATAAGAAGATAAGTTTACACAATCACCTGGAGAGCCACAAGCTTACCAGCAAGTC 1439

ZFX 1462 GGAGAAGGCCATTGAATGCGATGAGTGCGGAAAGCATTTCTCTCATGCTGGGGCTTTGTT 1521
ZFY 1440 AGAGAAGGCCATCGAATGTGATGACTGTGGGAAGCATTTCTCCCATGCTGGGGCTTTGTT 1499

ZFX 1522 TACTCATAAAATGGTGCATAAGGAAAAAGGAGCTAACAAAATGCACAAATGTAAATTCTG 1581
ZFY 1500 CACTCACAAAATGGTGCATAAGGAAAAAGGAGCCAGCAAAATGCATAAATGTAAATTCTG 1559

ZFX 1582 TGAATACGAGACAGCTGAACAAGGGTTACTGAATCGCCACCTTTTGGCGGTCCATAGCAA 1641
ZFY 1560 TGAGTATGAGACAGCTGAACAAGGGTTATTAAATCGCCACCTTTTGGCAGTCCACAGCAA 1619

ZFX 1642 GAACTTTCCTCATATATGCGTGGAGTGTGGTAAAGGTTTTCGTCATCCATCAGAGCTCAA 1701
ZFY 1620 GAACTTTCCCCATATATGTGTAGAGTGTGGTAAAGGTTTTCGTCACCCATCAGAGCTCAA 1679

ZFX 1702 AAAGCACATGCGAATCCATACTGGCGAGAAGCCGTACCAGTGCCAGTACTGCGAATATAG 1761
ZFY 1680 AAAGCACATGCGAATCCATACTGGAGAGAAACCGTACCAATGCCAGTACTGCGAATATAG 1739

ZFX 1762 GTCCGGCAGACTCTTCTAACTTGAAAACGCATGTAAAAACTAAGCATAGTAAAGAGATGCC 1821
ZFY 1740 GTCTGCAGACTCTTCTAATTTGAAGACGCATGTGAAAACTAAGCATAGTAAAGAAATGTC 1799

ZFX 1822 ATTCAAGTGTGACATTTGTCTTCTGACTTTCTCAGATACCAAAGAGGTCCAGCAACATGC 1881
ZFY 1800 TTTCAAGTGTGACATTTGTCTTCTGACTTTCTCCGATACCAAAGAGGTTCAGCAACATGC 1859
```

Primers | Mismatch to primers | *Pst*I recognition site | Restriction site ▲ | SNP

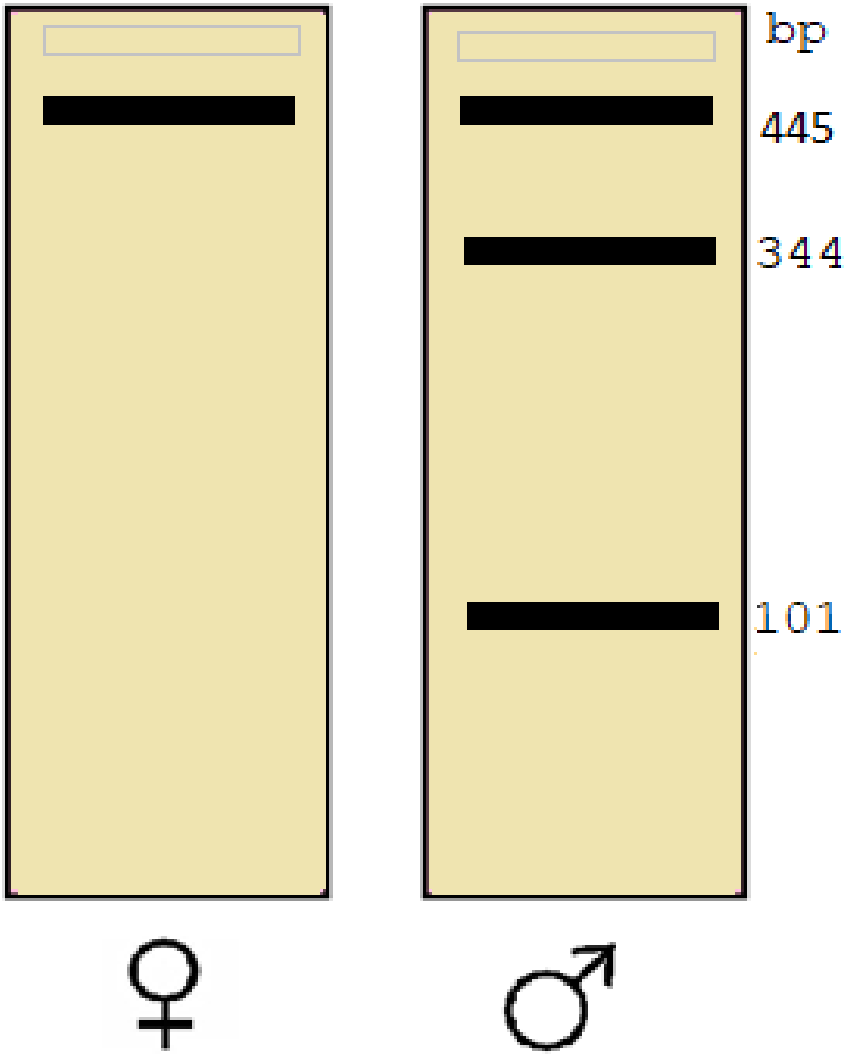

EMBL acc. No.: NM_177490.1, NM_177491.1

Water buffalo (*Bubalus bubalis*). *ZFX, ZFY*

Pande and Totey, 1998

```
ZFX 1528 ACACAACCACCTGGAGAGCCACAAGCTTACCAGCAAGGCGGAGAAGGCCATTGAATGCGA 1587
         |||||| |||||||||||||||||||||||||||||| | ||||||||||| ||||| ||
ZFY 900  ACACAATCACCTGGAGAGCCACAAGCTTACCAGCAAGTCAGAGAAGGCCATCGAATGTGA 959

ZFX 1588 TGAGTGCGGAAAGCATTTCTCTCATGCTGGGGCTTTGTTTACTCATAAAATGGTGCATAA 1647
         |||||| || ||||||||||| ||||||||||||||||| ||||| ||||||||||||||
ZFY 960  TGAGTGTGGGAAGCATTTCTCCCATGCTGGGGCTTTGTTCACTCACAAAATGGTGCATAA 1019

ZFX 1648 GGAAAAAGGAGCTAACAAAATGCATAAATGTAAATTCTGTGAATACGAGACAGCTGAACA 1707
         |||||||||||| | ||||||||||||||||||||||||||| || ||||||||||||||
ZFY 1020 GGAAAAAGGAGCAAGCAAAATGCATAAATGTAAATTCTGTGAGTATGAGACAGCTGAACA 1079

ZFX 1708 AGGGTTACTGAATCGCCACCTTTTGGCGGTCCATAGCAAGAACTTTCCTCATATATGCGT 1767
         ||||||| | ||||||||||||||||| ||||| ||||||||||||||||||||||| ||
ZFY 1080 AGGGTTATTAAATCGCCACCTTTTGGCAGTCCACAGCAAGAACTTTCCTCATATATGTGT 1139

ZFX 1768 GGAGTGTGGTAAAGGTTTTCGTCATCCATCAGAGCTCAAAAAGCACATGCGAATCCATAC 1827
          ||||||||||||||||||||||| |||||||||||||||||||||||||||||||||||
ZFY 1140 AGAGTGTGGTAAAGGTTTTCGTCACCCATCAGAGCTCAAAAAGCACATGCGAATCCATAC 1199

ZFX 1828 TGGCGAGAAGCCGTACCAATGCCAGTACTGCGAATATAGGTCTGCAGACTCTTCTAACTT 1887
         ||| ||||| || |||||||||||||||||||||||||||||||||||||||||||| ||
ZFY 1200 TGGAGAGAAACCATACCAATGCCAGTACTGCGAATATAGGTCTGCAGACTCTTCTAATTT 1259

ZFX 1888 GAAAACGCATGTAAAAACTAAGCATAGTAAAGAGATGCCATTCAAGTGTGACATTTGTCT 1947
         ||| |||||||| |||||||||||||||||||| ||| | ||||||||||||||||||||
ZFY 1260 GAAGACGCATGTGAAAACTAAGCATAGTAAAGAAATGTCTTTCAAGTGTGACATTTGTCT 1319

ZFX 1948 TCTGACTTTCTCAGATACCAAAGAGGTCCAGCAACATGCTCTTATCCACCAAGAAAGCAA 2007
         ||||||||| ||||||||||||||||| ||||||||||||||||||||||||||||||||
ZFY 1320 TCTGACTTTTTCAGATACCAAAGAGGTTCAGCAACATGCTCTTATCCACCAAGAAAGCAA 1379
```

Primers | Mismatch to primers | *Taq*I recognition site

F: 5′-ATAATCACATGGAGAGCCACAAGCT-3′

R1: 5′-GCACTTCTTTGGTATCTGAGAAAGT-3′

R2: 5′-CGAATGTGATGAGTGTG-3′

*Taq*I recognition site: 5′...TCGA...3′ / 3′...AGCT...5′

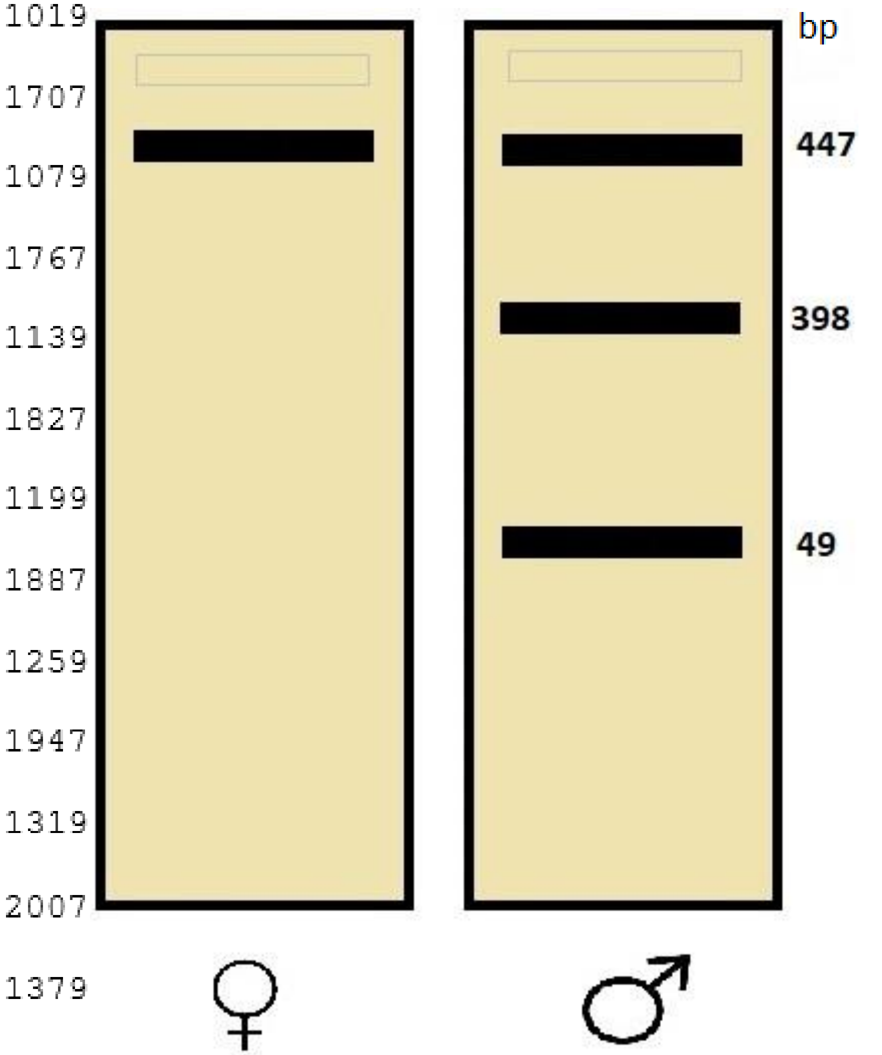

EMBL acc. No.:XM_006072177.1, GQ259330.1

Goat (*Capra hircus*). *AMELX, AMELY*

Tsai et al., 2011

F: 5′-AGCAACAGACAAGACCAAGC-3′

R: 5′-ACCCACCATAAAAGCTATTG-3′

R: 5′-TGCCATATAGATAGACAAGC-3′

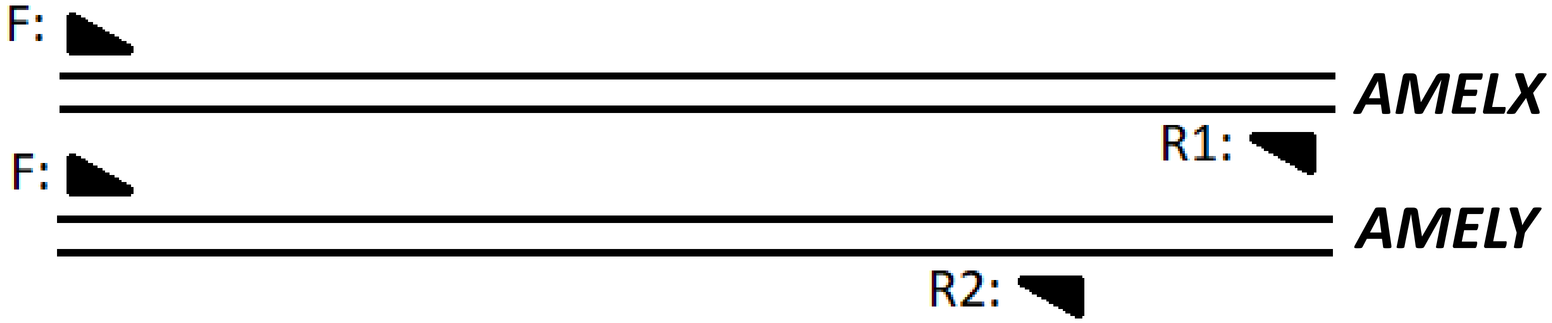

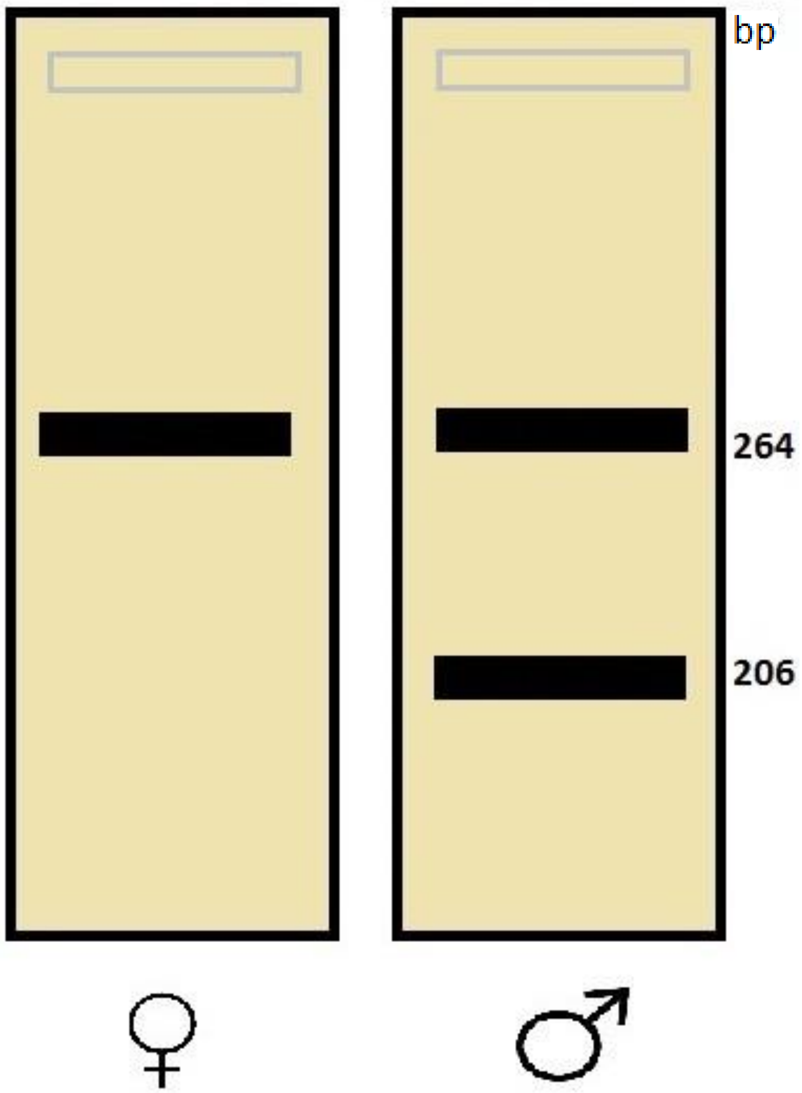

Goat (*Capra hircus*). *AMELX, AMELY*

Gokulakrishnan et al., 2013

F:5′-CCGCCCAGCAGYCCTTCCAG-3′

R:5′-TGGGAATATYGGAGGCAGAGG-3′

```
AMELX     CAGCCAAACCTCCCTCTGCCCGCCCAGCAGCCCTTCCAGCCCCAGTCCATCCAGCCGCAG
AMELY     ---------------------------------------------------------CAG
                             **************************************

AMELX     CCTCACCAGCCCCTGCAGCCCCTGCAGCCATGCAGCCCTTGCAGCCCTTGCAGCCCCTG
AMELY     CCAAACCTCCCTCTGCCCGCCCAGCAGC---CCTTCCAGCCACAGCCTCACCAACCCCTA
                                      ***

AMELX     CAGCCCCAGCCACCCGTGCACCCCATCCAGCCCTTGCCGCCACAGCCACCTCTGCCTCCG
AMELY     CAGCCCCAGCCACCTGTGCACCCCATCCAGCGCTTGCCACCACAGCCACCTCTGCCTTCA

AMELX     ATATTCCCCATGCAGCCTCTGCCCCCCATGCTTCCTGACCTGCCTCTGGAAGCTTGGCCA
AMELY     ATATTCCCCATGCAACCGCTGCCCCCTGTGCTTCCTGACCTGCCTCTGGAAGCTTGGCCA
Primers  Mismatch to primers  Diagnostic SSIndel ******
```

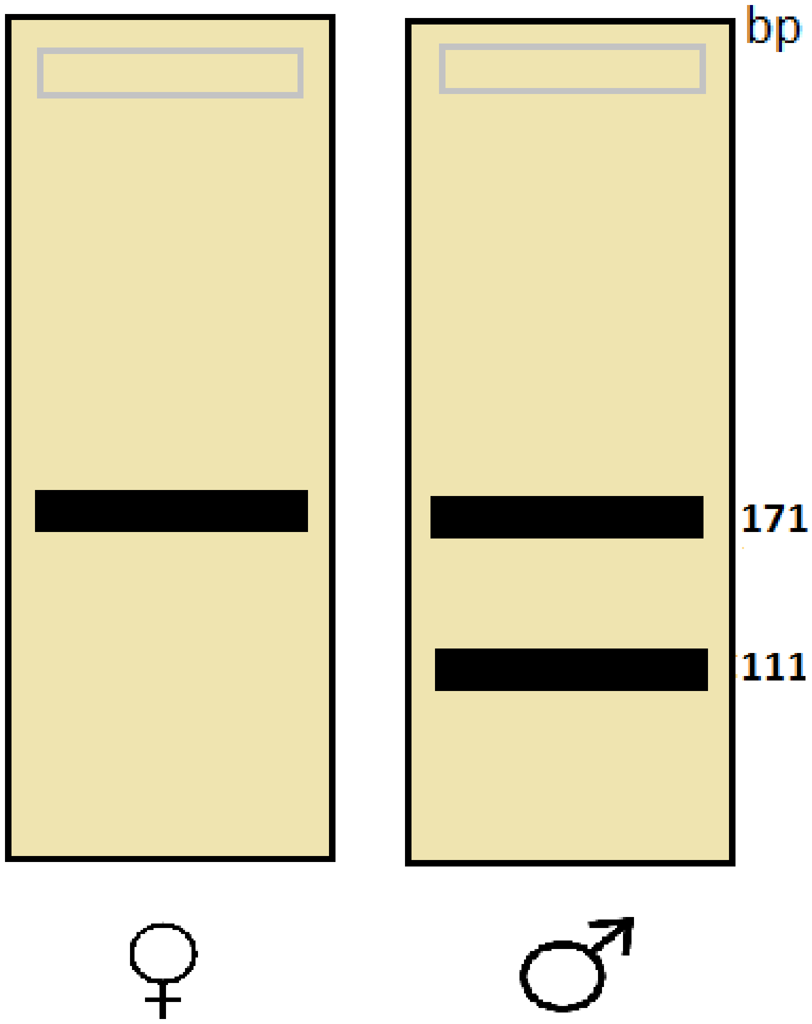

EMBL acc. No.: DQ469588.1, DQ469589.1

Goat (*Capra hircus*). *AMELX, AMELY*

Malik et al., 2013

F:5′-CAGCCAAACCTCCCTCTGC-3′
R:5′-CCCGCTTGGTCTTGTCTGTTGC-3′

```
AMELX   --------------------------------------------------------CAGC
AMELY   AGCAACCAATGATGCCAGTTCCTGGCCAACACTCCATGACTCCAATCCAACACCACCAGC

AMELX   CAAACCTCCCTCTGCCCGCCCAGCAGCCCTTCCAGCCCCAGTCCATCCAGCCGCAGCCTC
AMELY   CAAACCTCCCTCTGCCCGCCCAGCAGCCCTTCCAGCCACAGCCCATCCAGCCACAGCCTC

AMELX   ACCAGCCCCTGCAGCCCCTGCAGCCCATGCAGCCCTTGCAGCCCTTGCAGCCCCTGCAGC
AMELY   AC---------------------------------------------CAACCCCTACAGC
          *********************************************

AMELX   CCCAGCCACCCGTGCACCCCATCCAGCCCTTGCCGCCACAGCCACCTCTGCCTCCGATAT
AMELY   CCCAGCCACCTGTGCACCCCATCCAGCGCTTGCCACCACAGCCACCTCTGCCTTCAATAT

AMELX   TCCCCATGCAGCCTCTGCCCCCCATGCTTCCTGACCTGCCTCTGGAAGCTTGGCCAGCAA
AMELY   TCCCCATGCAACCGCTGCCCCCTG------------------------------------

AMELX   CAGACAAGACCAAGCGGG
AMELY   ------------------
```

Primers SSIndel ******

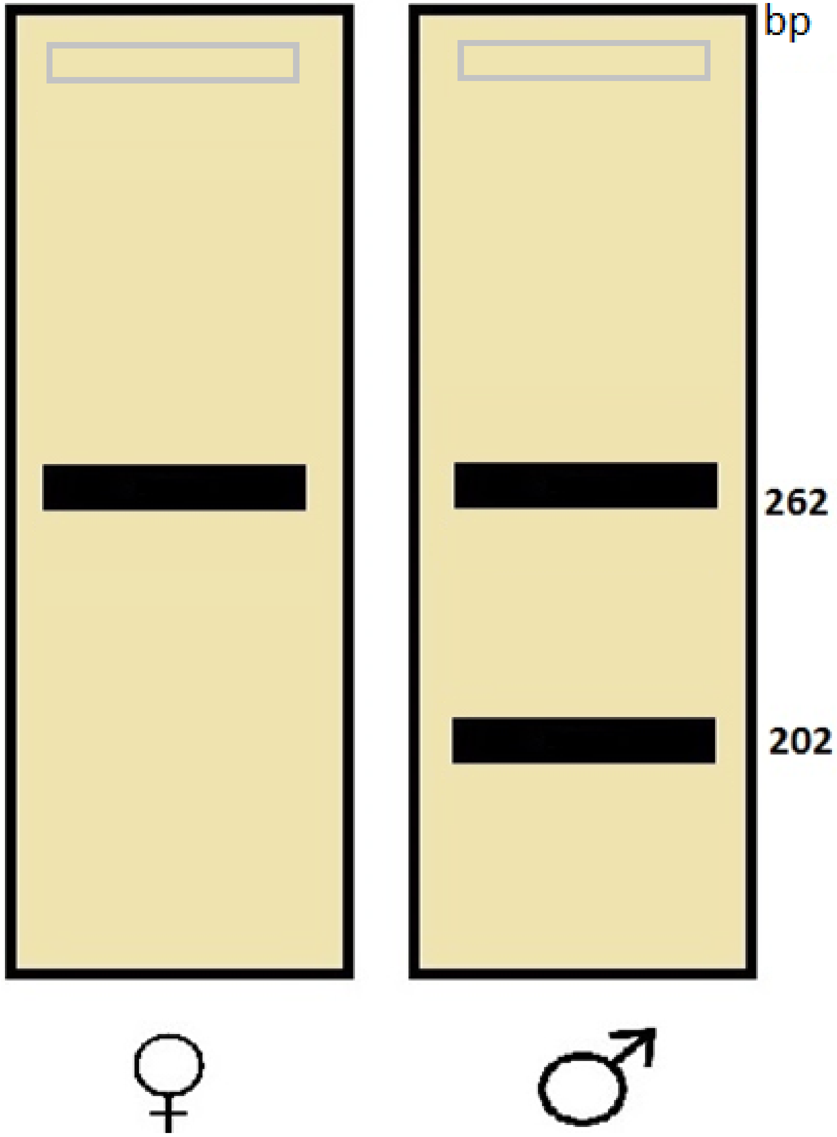

EMBL acc. No.: DQ469588, AY082491

Goat (*Capra hircus*). *ZFX, ZFY*

Aasen and Medrano, 1990

F: 5′-ATAATCACATGGAGAGCCACAAGCT-3′

R: 5′-GCACTTCTTTGGTATCTGAGAAAGT-3′

*Sac*I recognition site: 5′...GAGCTC...3′
3′...CTCGAG...5′

```
ZFX 1816  CACAACCACCTGGAGAGCCACAAGCTTACCAGCAAGGCGGAGAAGGCCATTGAATGCGAT  1875
          ||||| |||||||||||||||||||||||||||||||| ||||||||||| ||||| |||
ZFY 1591  CACAATCACCTGGAGAGCCACAAGCTTACCAGCAAGGCAGAGAAGGCCATCGAATGTGAT  1650

ZFX 1876  GAGTGCGGAAAGCATTTCTCTCATGCTGGGGCTTTGTTTACTCATAAAATGGTGCATAAG  1935
          || || || ||||||||||| ||||||||||||||||| ||||| |||||||||||||||
ZFY 1651  GAATGTGGGAAGCATTTCTCCCATGCTGGGGCTTTGTTCACTCACAAAATGGTGCATAAG  1710

ZFX 1936  GAGAAAGGAGCCAACAAAATGCACAAATGTAAATTCTGTGAATATGAGACAGCTGAACAA  1995
          || |||||||||| ||||||||| ||||||||||||||||| ||||||||||||||||||
ZFY 1711  GAAAAAGGAGCCAGCAAAATGCATAAATGTAAATTCTGTGAGTATGAGACAGCTGAACAA  1770

ZFX 1996  GGGTTACTGAATCGCCACCTTTTGGCGGTCCATAGCAAAAACTTTCCTCATATATGCGTG  2055
          |||||| | ||||||||||||||||| ||||| ||||| ||||||||||||||||| || 
ZFY 1771  GGGTTATTAAATCGCCACCTTTTGGCAGTCCACAGCAAGAACTTTCCTCATATATGTGTA  1830

ZFX 2056  GAGTGTGGTAAAGGTTTTCGTCATCCATCAGAGCTCAAAAAGCACATGCGAATCCATACT  2115
          ||||||||||||||||||||||| ||||||||||| | ||||||||||||||||||||||
ZFY 1831  GAGTGTGGTAAAGGTTTTCGTCACCCATCAGAGCTTAGAAAGCACATGCGAATCCATACT  1890

ZFX 2116  GGCGAGAAGCCGTACCAATGCCAGTACTGCGAATATAGGTCTGCAGACTCTTCTAACTTG  2175
          || ||||| ||||||||||||||||||||||||||||||||||||||||||||||| |||
ZFY 1891  GGAGAGAAACCGTACCAATGCCAGTACTGCGAATATAGGTCTGCAGACTCTTCTAATTTG  1950

ZFX 2176  AAAACGCATGTAAAAACTAAGCATAGTAAAGAGATGCCATTCAAGTGTGACATTTGTCTT  2235
          ||||||||||| |||||||||||||||||||| ||| | |||||||||||||||||||||
ZFY 1951  AAAACGCATGTGAAAACTAAGCATAGTAAAGAAATGTCTTTCAAGTGTGACATTTGTCTT  2010

ZFX 2236  CTGACTTTCTCAGATACCAAAGAGGTCCAGCAACATGCTCTTATCCACCAAGAAAGCAAA  2295
          |||||||||||||||||||||||||| |||||||||||||||||||||||||||||||||
ZFY 2011  CTGACTTTCTCAGATACCAAAGAGGTTCAGCAACATGCTCTTATCCACCAAGAAAGCAAA  2070
```

Primers | Mismatch to primers | *Sac*I recognition site | Restriction site▲

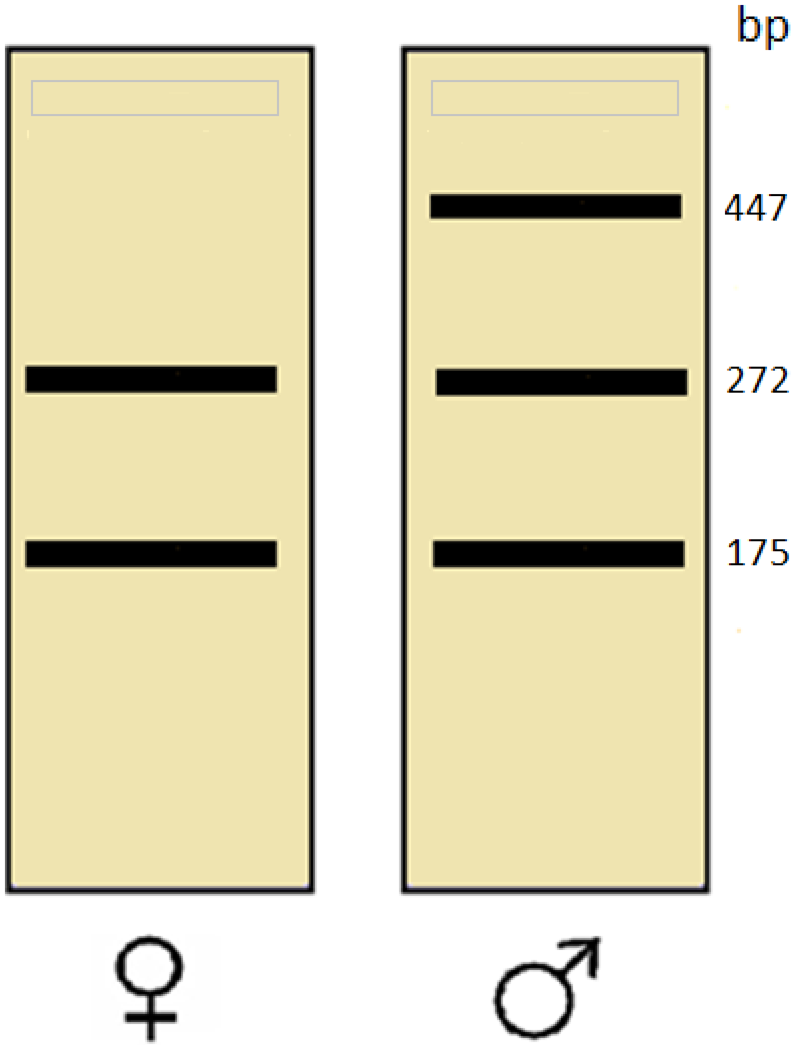

EMBL acc. No.: XM_018043816.1, XM_018044893.1

Sheep (*Ovis aries*). *AMELX, AMELY*

Pfeiffer and Brening, 2005

F:5′-AGCCAAACCTCCCTCTGC-3′

R:5′-CCCGCTTGGTCTTGTCTGTTGC-3′

```
AMELX      AGCCAAACCTCCCTCTGCCCGCCCAGCAGCCCTTCCAGCCCCAGTCCATCCAGCCGCAGC
AMELY      --------------------------------------------------------CAGC
                             **************************************

AMELX      CTCACCAGCCCCTGCAGCCCCTGCAGCCCATGCAGCCCTTGCAGCCCTTGCAGCCCCTGC
AMELY      CAAACCTCCCTCTGCCCGCCCAGCAGC---CCTTCCAGCCACAGCCTCACCAACCCCTAC
                                      ***

AMELX      AGCCCCAGCCACCCGTGCACCCCATCCAGCCCTTGCCGCCACAGCCACCTCTGCCTCCGA
AMELY      AGCCCCAGCCACCTGTGCACCCCATCCAGCGCTTGCCACCACAGCCACCTCTGCCTTCAA

AMELX      TATTCCCCATGCAGCCTCTGCCCCCCATGCTTCCTGACCTGCCTCTGGAAGCTTGGCCAG
AMELY      TATTCCCCATGCAACCGCTGCCCCCTGTGCTTCCTGACCTGCCTCTGGAAGCTTGGCCAG

AMELX      CAACAGACAAGACCAAGCGGGAGGAAGTG   269
AMELY      CAACAGACAAGACCAAGCGGG--------   202
Primers SSIndel *****
```

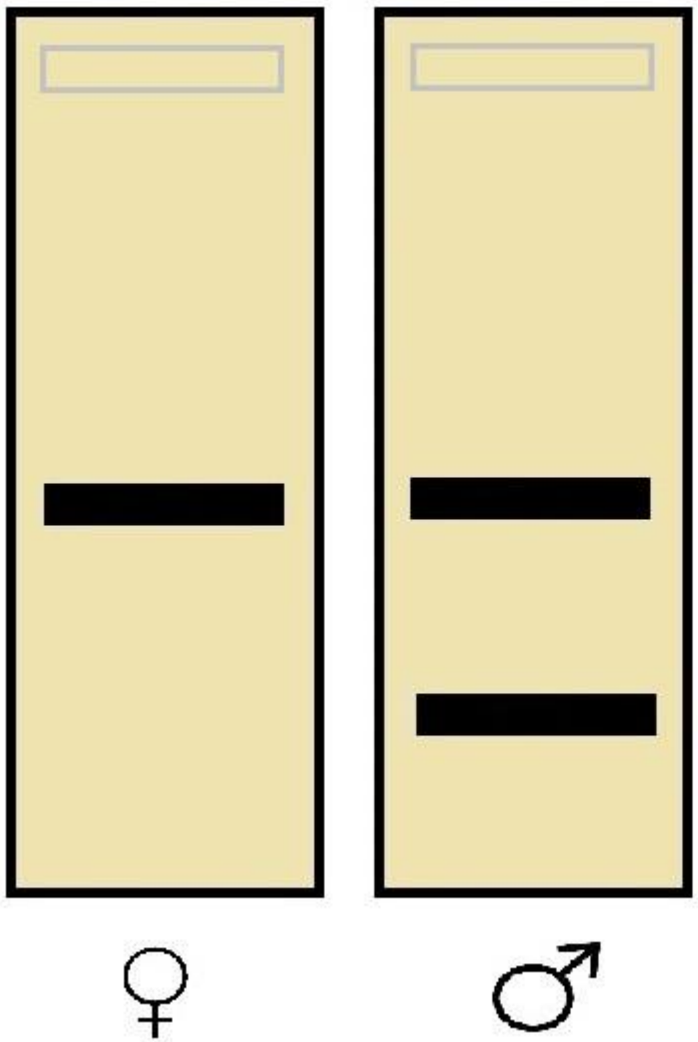

Exact fragment sizes could not be determined.

EMBL acc. No.: ENSOARG00000010966, DQ469592.1

Sheep (*Ovis aries*). *AMELX, AMELY*

Gokulakrishnan et al., 2013

F:5′-CCGCCCAGCAGYCCTTCCAG-3′
R:5′-TGGGGAATATYGGAGGCAGAGG-3′

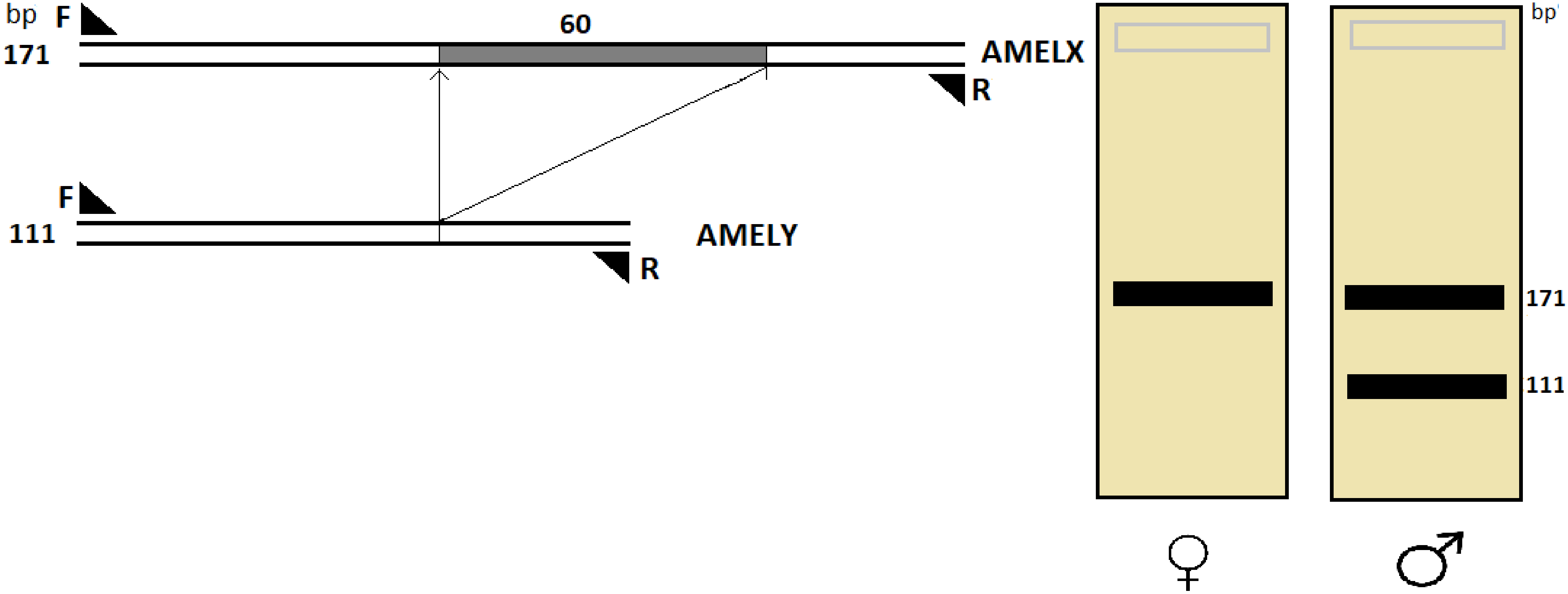

Sheep (*Ovis aries*). *ZFX, ZFY*

Aasen and Medrano, 1990

F:5′-ATAATCACATGGAGAGCCACAAGCT-3′
R:5′-GCACTTCTTTGGTATCTGAGAAAGT-3′

*Sac*I recognition site: 5′...GAGCTC...3′
3′...CTCGAG...5′

```
ZFX  1389  ACAACCACCTGGAGAGCCACAAGCTTACCAGCAAGGCGGAGAAGGCCATTGAATGCGATGAG  1448
           | || ||| |||||||||||||||||||||||||||||||||||||||| ||||| |||||
ZFY  3     ATAATCACATGGAGAGCCACAAGCTTACCAGCAAGGCGGAGAAGGCCATCGAATGTGATGAA  62

ZFX  1449  TGCGGAAAGCATTTCTCTCATGCTGGGGCTTTGTTTACTCATAAAATGGTGCATAAGGAG  1508
           || || ||||||||||| ||||||||||||||||| ||||| ||||||||||||||||||
ZFY  63    TGTGGGAAGCATTTCTCCCATGCTGGGGCTTTGTTCACTCACAAAATGGTGCATAAGGAG  122

ZFX  1509  AAAGGAGCCAACAAAATGCACAAATGTAAATTCTGTGAATATGAGACAGCTGAACAAGGG  1568
           |||||||||| ||||||||| ||||||||||||||||| |||||||||||||||||||||
ZFY  123   AAAGGAGCCAGCAAAATGCATAAATGTAAATTCTGTGAGTATGAGACAGCTGAACAAGGG  182

ZFX  1569  TTACTGAATCGCCACCTTTTGGCGGTCCATAGCAAAAACTTTCCTCATATATGCGTGGAG  1628
           ||| ||||||||||||||||||| ||||| ||||| ||||||||||||||||| || |||
ZFX  183   TTATTGAATCGCCACCTTTTGGCAGTCCACAGCAAGAACTTTCCTCATATATGTGTAGAG  242

ZFX  1629  TGTGGTAAAGGTTTTCGTCATCCATCAGAGCTCAAAAAGCACATGCGAATCCATACTGGC  1688
           |||||||||||||||||||| ||||||||||| | |||||||||||| |||||||||||
ZFY  243   TGTGGTAAAGGTTTTCGTCACCCATCAGAGCTTAGAAAGCACATGCGGATCCATACTGGA  302

ZFX  1689  GAGAAGCCGTACCAATGCCAGTACTGCGAATATAGGTCTGCAGACTCTTCTAACTTGAAA  1748
           ||||| ||||||||||||||||||||||||||||||||||||||||||||| ||||||
ZFY  303   GAGAAACCGTACCAATGCCAGTACTGCGAATATAGGTCTGCAGACTCTTCTAATTTGAAA  362

ZFX  1749  ACGCATGTAAAAACTAAGCATAGTAAAGAGATGCCATTCAAGTGTGACATTTGTCTTCTG  1808
           |||||||| |||||||||||||||||||||||| | ||||||||||| ||||||||||||
ZFY  363   ACGCATGTGAAAACTAAGCATAGTAAAGAGATGTCTTTCAAGTGTGAAATTTGTCTTCTG  422

ZFX  1809  ACTTTCTCAGATACCAAAGAGGTCC  1828
           |||||||||||||||||||| || |
ZFY  423   ACTTTCTCAGATACCAAAGAAGTGC  442
```

Primers *Sac*I recognition site

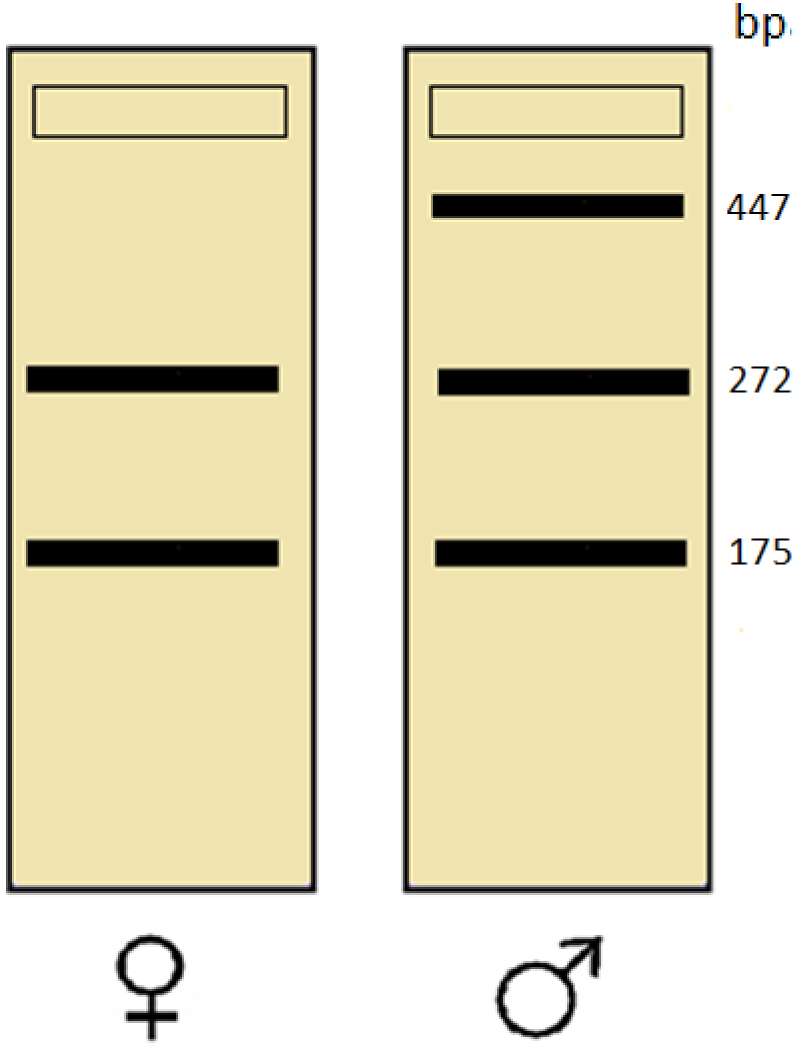

EMBL acc. No.: XM_015104836.1, AJ000270.1

Humpback whale (*Megaptera novaeangliae*), fin whale (*Balaenoptera physalus*), blue whale (*Balaenoptera musculus*), minke whale (*Balaenoptera acutorostrata*), beluga (*Delphinapterus leucas*), narwhal (*Monodon monoceros*), harbour porpoise (*Phocoena phocoena*). *ZFX, ZFY*

Bérubé and Palsbøll, 1996

F: 5′-ATAGGTCTGCAGACTCTTCTA-3′

R: 5′-AGAATATGGCGACTTAGAACG-3′

R: 5′-TTTGTGTGAACTGAAATTACA-3′

R: 5′-CACTTATGGGGTAGTCCTTT-3′

R: 5′-ATTACATGTCGTTTCAAATCA-3′

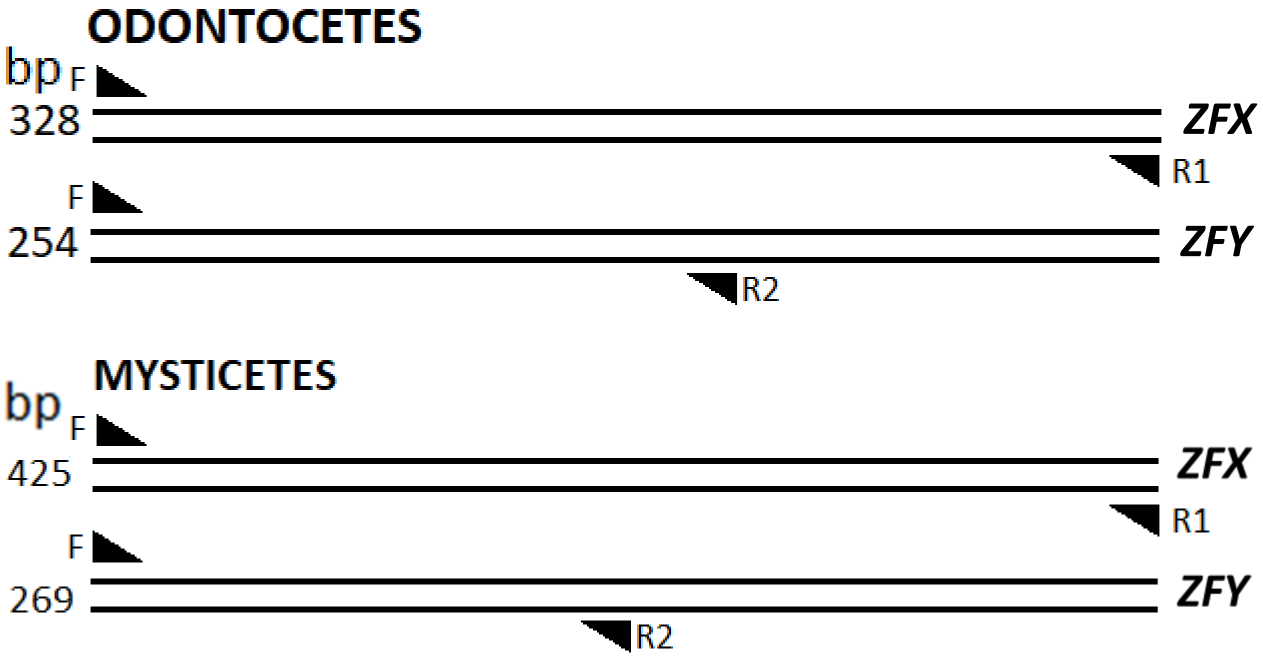

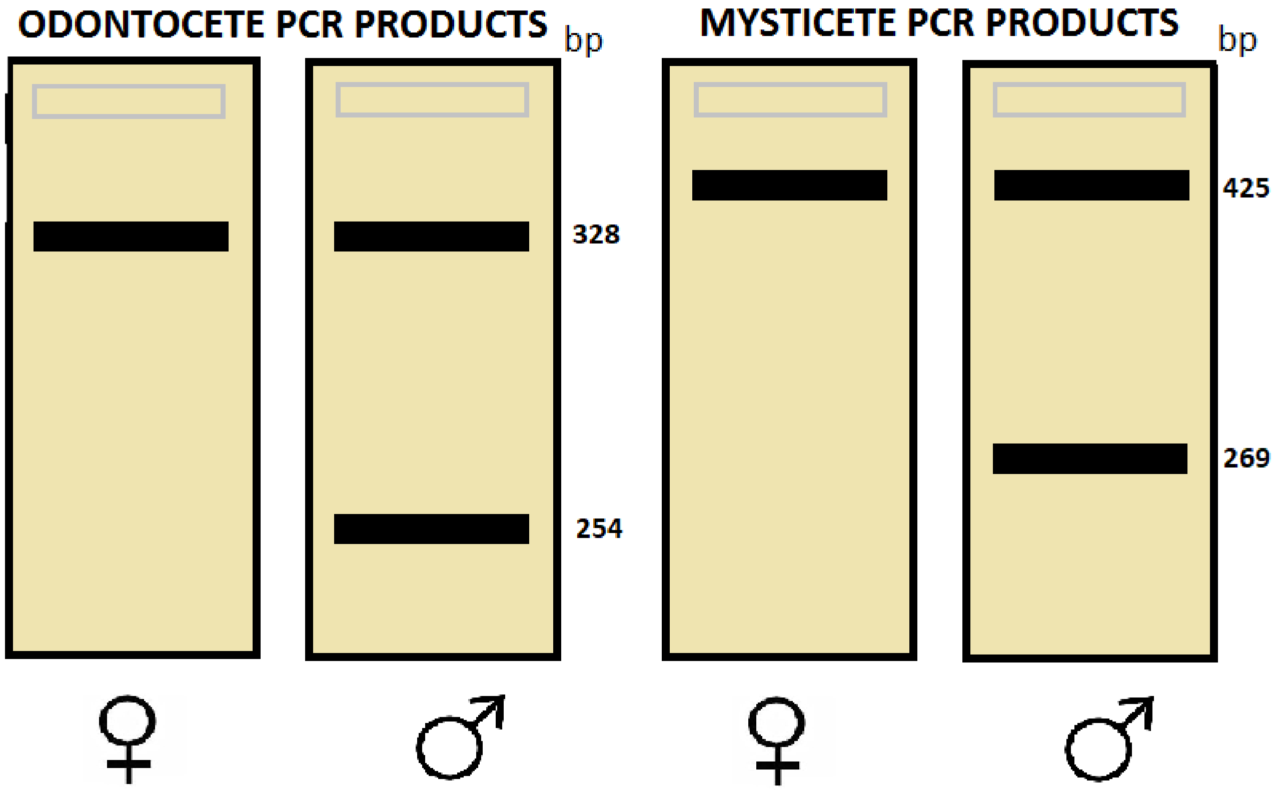

Bowhead whale (*Balaena mysticetus*), Grey whale (*Eschrichtius robustus*), Sperm whale (*Physeter microcephalus*), Pigmy sperm whale (*Kogia breviceps*), *Dwarf sperm whale* (*Kogia sima*), Beluga (*Delphinapterus leucas*), Eastern spinner dolphin (*Stenella longirostris orientalis*), *Whitebelly spinner dolphin* (*Stenella longirostris hybrid*), Harbor porpoise (*Phocoena phocoena*), Finless porpoise (*Neophocaena phocaenoides*). *ZFX, ZFY*

Morin et al., 2005

```
ZFX  1    CATCCTTTGACTGTCTATCCTTGCATGATTTGTGGAAAAAGTTTAAGTCGAGAGGTTTT   60
          ||||||||||||||||||||||| |||||||||||||||||||||||||||||||| |||
ZFY  1    CATCCTTTGACTGTCTATCCTTGTATGATTTGTGGAAAAAGTTTAAGTCGAGAGGGTTT   60

ZFX  61   TTGAAAAGGCACATGAAAAACCATCCTGAACACCTTACCAAGAAGAAGTACCGCTGTACT  120
          ||||||||||||||||||||||| ||||||||||| ||||||||||||||||||||||||
ZFY  61   TTGAAAAGGCACATGAAAAACCACCCTGAACACCTCACCAAGAAGAAGTACCGCTGTACT  120

ZFX  121  GACTGTGATTACACTACCAACAAGAAGATAAGTTTACACAACCACCTGGAGAGCCACAAG  180
          ||||||||||||||||||||||||||||||||||||||||||||||||||||||||||||
ZFY  121  GACTGTGATTACACTACCAACAAGAAGATAAGTTTACACAACCACCTGGAGAGCCACAAG  180
```

Primer Probe CETZFX Probe CETZFY

EMBL acc. No.: AF260784, AF260785

Bottlenose dolphin (*Tursiops truncatus*), bridled dolphin (*Stenella attenuata),* Clymene dolphin (*Stenella clymene),* striped dolphin (*Stenella coeruleoalba*), Atlantic spotted dolphin (*Stenella frontalis*), spinner dolphin (*Stenella longirostris*), saddleback dolphin (*Delphinus delphis*), harbor porpoise (*Phocoena phocoena*), boutu (*Inia geoffrensis*), false killer *whale (Pseudorca crassidens*). *ZFX, SRY*

Rosel et al., 2003

ZFX:

F:5′-ATAGGTCTGCAGACTCTTCTA-3′

R:5′-AGAATATGGCGACTTAGAACG-3′

SRY:

F:5′-CATTGTGTGGTCTCGTGATC-3′

R:5′-ACCGGCTTTCCATTCGTGAACG-3′

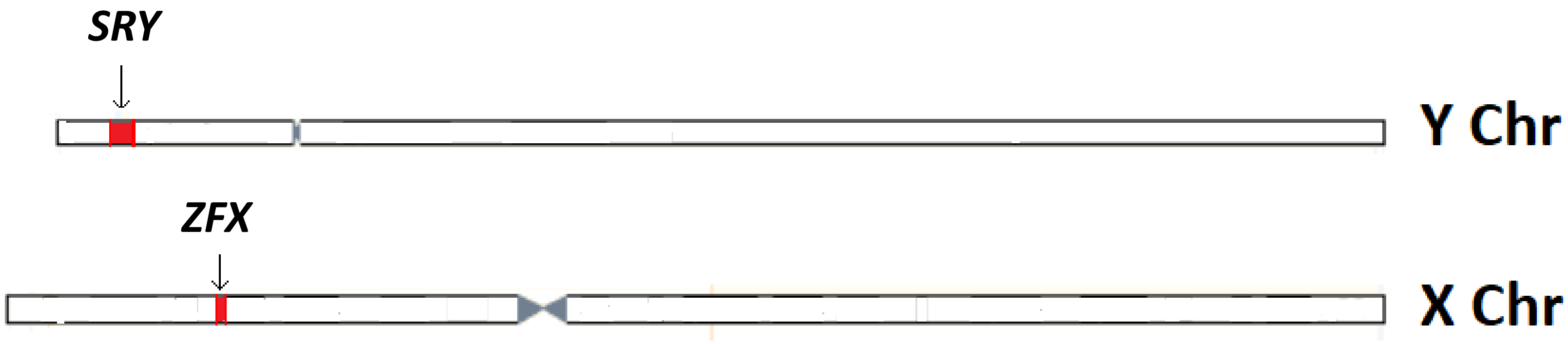

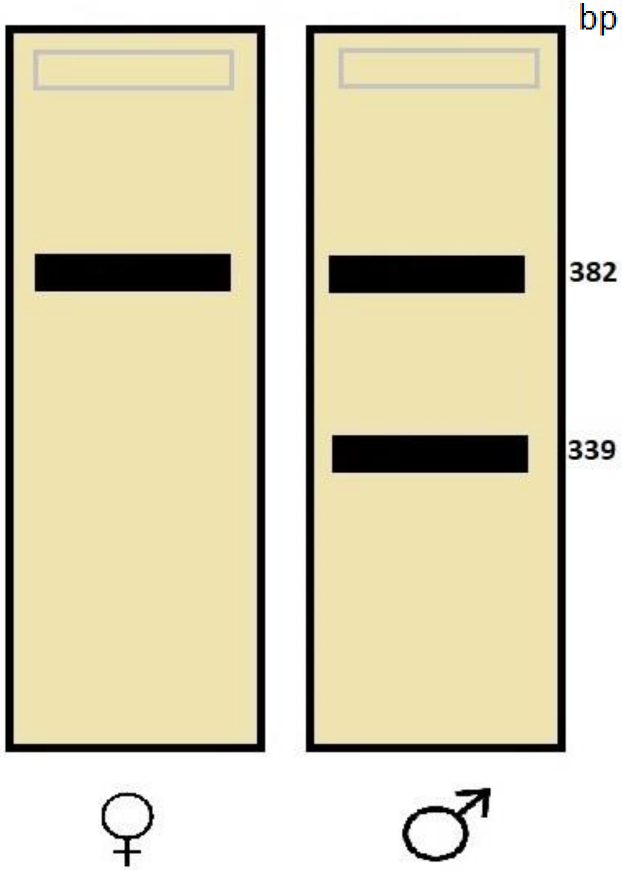

Domestic pig (*Sus scrofa domesticus*). *AMELX, AMELY*

Fontanesi et al., 2008

```
AMELX  1    TTTTGACCATTGTTTGACTTAACAATGCCCTGGGCTCTGTAAAGAATAGTGGGTGGATTC  60
            ||||||||||||||||||||||||||||||||||||||||||||||||||||||||||||
AMELY  1    TTTTGACCATTGTTTGACTTAACAATGCCCTGGGCTCTGTAAAGAATAGTGGGTGGATTC  60

AMELX  61   TTCATTCAGGATGTTTGTCAGTCCCATTTTTCAGTTCTCACTGCCAGCTTCCTAGTTTA  120
            ||||||||||||||||||||||||||||||||||||||||||||||||||||||||||||
AMELY  61   TTCATTCAGGATGTTTGTCAGTCCCATTTTTCAGTTCTCACTGCCAGCTTCCTAGTTTA  120

AMELX  121  AGCCCTGATGGGTCACCTCAAGCCTGCATTGCCCCAGAACCCTCCTACCTGcccccccac  180
            |||||||||||||||||||||||||||||||||||||||||||||||||||||||
AMELY  121  AGCCCTGATGGGTCACCTCAAGCCTGCATTGCCCCAGAACCCTCCTACCTGCCCC-----  175
                                                                   ******

AMELX  181  ccaacccccGACTCAGTCTCTCCTCCGTATACGGCTGTAAAATGAACACCCCCTGGAGGG  240
                ||||||||||||||||||||| ||||||| ||||||||||||||||||||||||||
AMELY  176  ----CCCCCGACTCAGTCTCTCCTCTGTATACAGCTGTAAAATGAACACCCCCTGGAGGG  231
            *****

AMELX  241  GGGACGACATGGTAGGGCAGAAACTGAACTCTGGCTGACCAGAGTTCTATCCCGGCCTGG  300
            |||||| |||||||||||||||||||||||||||||||||||||||||||||||||||||
AMELY  232  GGGACGGCATGGTAGGGCAGAAACTGAACTCTGGCTGACCAGAGTTCTATCCCGGCCTGG  291

AMELX  301  AAAATATGGGACTCAGGTAAGATGTTATCAACCTAAGGTCCTTGCCAGCCAGACCACTC  360
            ||||||||||||||||||||||||||||||||||||||||||| ||||||||||||||||
AMELY  292  AAAATATGGGACTCAGGTAAGATGTTATCAACCTAAGGTCCTTTCCAGCCAGACCACTC  351

AMELX  361  CTGGTTCTAAGACGTGCACACTCTACGTGTCTCCCTCGCT  400
            ||||||||||||||||||||||||||||||||||||||||
AMELY  352  CTGGTTCTAAGACGTGCACACTCTACGTGTCTCCCTCGCT  391
```

Primer pair Amel_2   SSIndel*******

Amel_2

F: 5′-GTTTAAGCCCTGATGGGTCA-3′

R: 5′-CCGGGATAGAACTCTGGTCA-3′

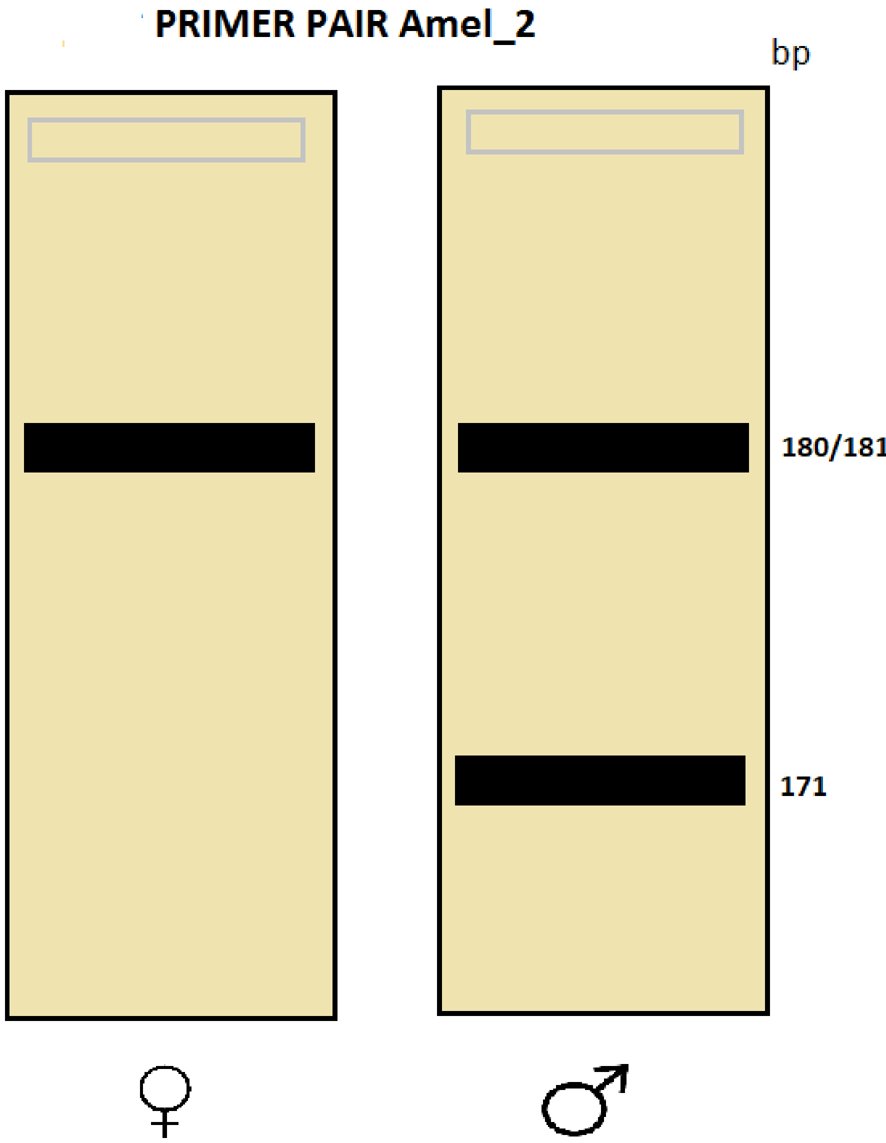

EMBL acc. No.: AM884151, AM884153

Domestic pig (Sus scrofa domesticus). AMELX, AMELY

Fontanesi et al., 2008

```
ZFX   1   TTTTGACCATTGTTTGACTTAACAATGCCCTGGGCTCTGTAAAGAATAGTGGGTGGATTC  60
          ||||||||||||||||||||||||||||||||||||||||||||||||||||||||||||
ZFY   1   TTTTGACCATTGTTTGACTTAACAATGCCCTGGGCTCTGTAAAGAATAGTGGGTGGATTC  60

ZFX   61  TTCATTCAGGATGTTTGTCAGTCCCATTTTTCAGTTCTCACTGCCAGCTTCCTAGTTTA  120
          ||||||||||||||||||||||||||||||||||||||||||||||||||||||||||||
ZFY   61  TTCATTCAGGATGTTTGTCAGTCCCATTTTTCAGTTCTCACTGCCAGCTTCCTAGTTTA  120

ZFX   121 AGCCCTGATGGGTCACCTCAAGCCTGCATTGCCCCAGAACCCTCCTACCTGCCCCCCCAC  180
          |||||||||||||||||||||||||||||||||||||||||||||||||||||||
ZFY   121 AGCCCTGATGGGTCACCTCAAGCCTGCATTGCCCCAGAACCCTCCTACCTGCCCC-----  175
                                                                ******

ZFX   181 CCAACCCCCGACTCAGTCTCTCCTCCGTATACGGCTGTAAAATGAACACCCCCTGGAGGG  240
              ||||||||||||||||||||| |||||| |||||||||||||||||||||||||||
ZFY   176 ----CCCCCGACTCAGTCTCTCCTCTGTATACAGCTGTAAAATGAACACCCCCTGGAGGG  231
          *****

ZFX   241 GGGACGACATGGTAGGGCAGAAACTGAACTCTGGCTGACCAGAGTTCTATCCCGGCCTGG  300
          |||||| |||||||||||||||||||||||||||||||||||||||||||||||||||||
ZFY   232 GGGACGGCATGGTAGGGCAGAAACTGAACTCTGGCTGACCAGAGTTCTATCCCGGCCTGG  291
```

Primer pair Amel_3 SSIndel ******

Amel_3

F:5′-GGGTGGATTCTTCATTCAGG-3′

R:5′-TCCAGGGGGTGTTCATTTTA-3′

PRIMER PAIR Amel_3

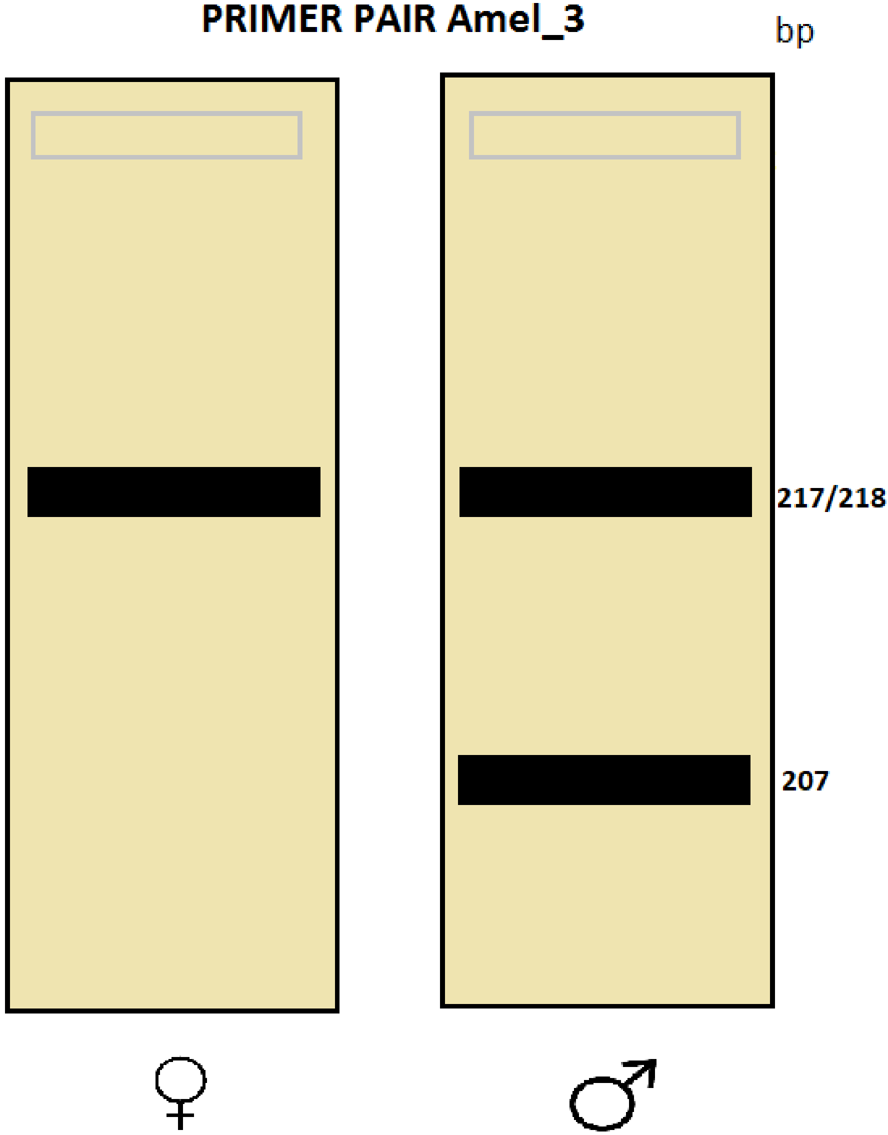

EMBL acc. No.: AM884151, AM884153

Domestic pig (Sus scrofa domesticus). ZFX, SRY

Blanes et al., 2018

SRY

FW: 5'-GGGAAAGGCTCCTCACTATTT-3'

RW: 5'-AGGGATACATCCTCTCCTCTAC-3'

ZFX

FW: 5'-GTGCTGCTTTGTCTTGGAATG-3'

RW: 5'-GAGGGAGTTAGGTCTGGATACT-3'

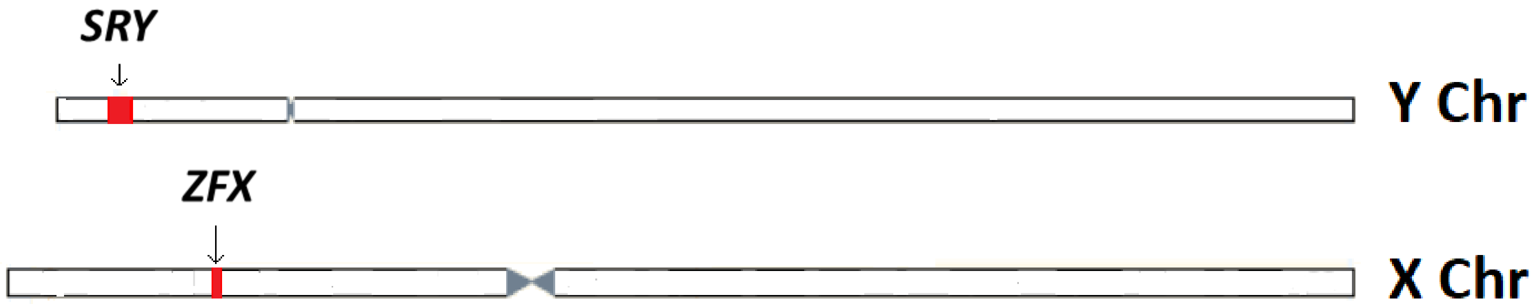

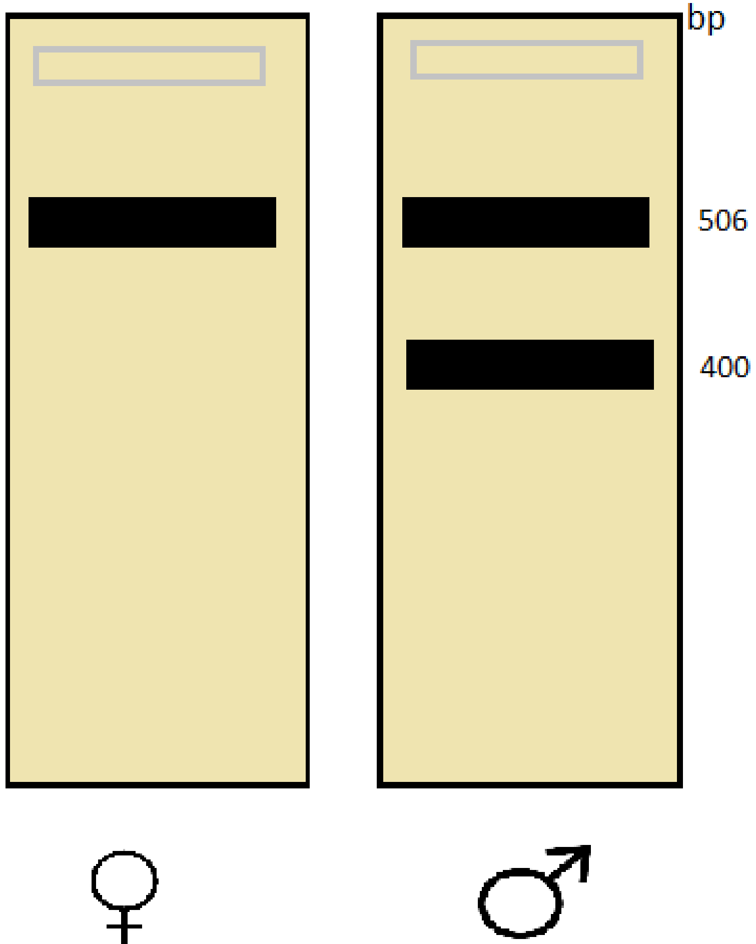

The common hippopotamus (*Hippopotamus amphibius*). *ZFX, ZFY*

Beckwitt et al., 2002

F:5′-ATAATCACATGGAGAGCCACAAGCT-3′

R:5′-GCACTTCTTTGGTATCTGAGAAAGT-3′

*Hae*III recognition site: 5′...GGCC...3′ / 3′...CCGG...5′

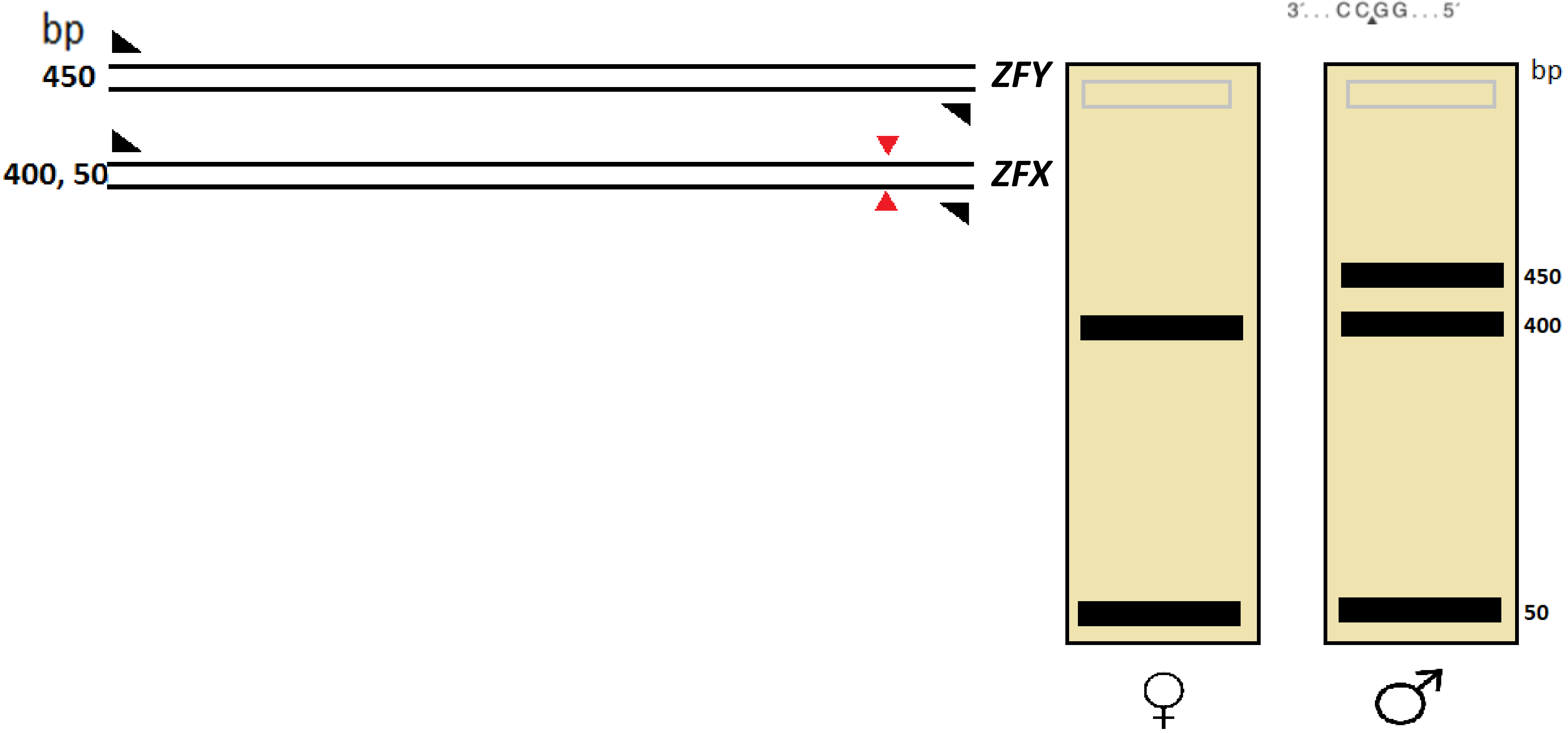

Horse (*Equus caballus*). *ZFX, ZFY*

Han et al., 2010

```
ZFX 1    AAATCAAAACCTTCATGCCAATAGCATGGGCAGCCGCTTACGGTGAGTCACAGAGCAGCT 60
ZFY 1    AAATCAAAACCTTCATGCCAATAGCATGGGCAGCAGCTTATGGTAAGTCACATAGCAGTT 60

ZFX 61   CTTGGGATTGAGTTGGTTCTTGATCATGAATTCATGATAGGAAAACAGTTTCTGTGGTCT 120
ZFY 61   CAAGG-ATTGCATAGTTT-TGAAACGTGAATTCAGTATTGAAAAATAGTTGCTATGGTCT 118

ZFX 121  TAGGTTTAAGAAATCTGAAATACCAGTACCCTGGAAGGGTTATTTGATTTGAACAAGAGT 180
ZFY 119  TAGGTTTCAGAAATATGAAGTATTAGCAC-----AA--------TGCTTTAAGCAAAAAT 165

ZFX 181  AGAGGAAGATTATAAAGTCTACTTTTTGTCTTTATTTTTAACAGGAATTTCCTTCATGTG 240
ZFY 166  AGAGGAA----------TTTCCTTTTTGCCTTTA------AGAGGAATTTCCTACATGCC 209

ZFX 241  TTTATTACGTAGAAGGAAGTACTGCAAGAAGTACACAGGCTTTCAAGCTGAAACTTTTC- 299
ZFY 210  CATATTATGTT---------------AGAAGT-CATAGGCTTTCAAGCTGAAATTTTTTT 253

ZFX 300  ATCTTGATTATATCTGGCCCAGGACTTTACAATTTTGGGAA--ACAACAAGGGAGTCCAT 357
ZFY 254  ATCTTGAGTATATCCGGCTCATGACTTTATAGTATTGAGTTTGACAACAAGCATGTTCAT 313

ZFX 358  GGGGCAAAACGCTGACATGTGTTTTTTAGATTTGGAAGCTAGGCATTTCCTACAGTCATG 417
ZFY 314  GGGGCAAACTGCTGC-----------TAGATTTAGAAACTAGGTATTTCCT---GTCATC 359

ZFX 418  CTATAA-TAAATGT--------CATAAA-CCCATTACCTAGAATGGGGAATTTCTGTCAT 467
ZFY 360  TTGTGTGTAAATCTAGTATGCCCATAAAGCCCATTACCTAGA-TAGGGAATTTCTGTCAT 418

ZFX 468  TCATGAGTATCATGGCTTACTTTTCGTTGTTATAGTTAGTAAACAATCCCTAATTCATTA 527
ZFY 419  TCATGACTATCATGGCTT----TTCATTGTTCTCTTTAATGTAGAATACCTAATTCTTTA 474

ZFX 528  TAATTTATA--ATACTGTATAATTTTGTTTTTTAATGCGCATTGTTAGGTAATAATTCTG 585
ZFY 475  TAATTTAAAGAATACTGTGTAATTTTGTTTTTTAATACACATTGTTAGGTAATAATTCTG 534

ZFX 586  ATGGAATTGAAAACCGGAA 604
ZFY 535  ATGGAATTGAAAACCGGAA 553
```

Primers SSIndel ******

EMBL acc. No.: DQ179230 and DQ179229

F:5′-AAATCAAAACCTTCATGCCAA-3′

R:5′-TTCCGGTTTTCAATTCCA-3′

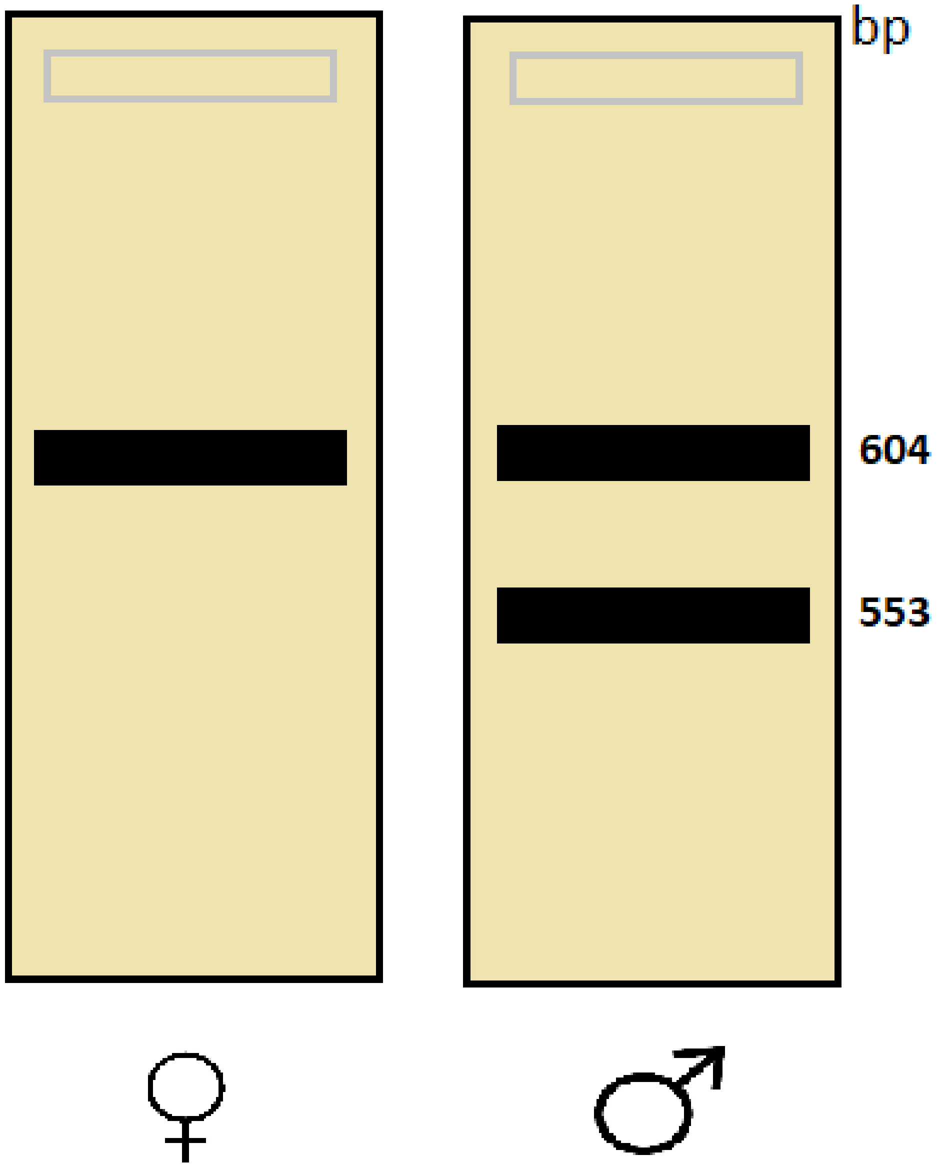

Indian rhinoceros (*Rhinoceros unicornis*). *ZFX, ZFY*

Fernando and Melnick, 2001

F:5′-ATAATCACATGGAGAGCCACAAGCT-3′
R:5′-GCACTTCTTTGGTATCTGAGAAAGT-3′

*Nsi*I recognition site: 5′...ATGCA▼T...3′ / 3′...T▲ACGTA...5′

```
ZFX   1    GTGGGAAGCATTTCTCTCATGCTGGGGCTTTGTTTACTCACAAAATGGTGCATAAGGAGA   60
           ||||||||||||||||||||||||||||||||||||| ||||||||||||||||||||||
ZFY   1    GTGGGAAGCATTTCTCTCATGCTGGGGCTTTGTTTACGCACAAAATGGTGCATAAGGAGA   60

ZFX   61   AAGGAGCCAACAAAATGCACAAGTGTAAATTCTGTGAATATGAGACAGCTGAACAAGGGT   120
           |||||||||  |||||||| ||||||||||||||||||||||||||||||||||||||||
ZFY   61   AAGGAGCCAGTAAAATGCATAAGTGTAAATTCTGTGAATATGAGACAGCTGAACAAGGGT   120
                           *****▲

ZFX   121  TATTGAACCGCCACCTTTTGGCGGTCCACAGCAAGAACTTTCCTCATATTTGCGTGGAGT   180
           ||||||||||||||||||||||||||||||||||||||||||||||||||||||||||||
ZFY   121  TATTGAACCGCCACCTTTTGGCGGTCCACAGCAAGAACTTTCCTCATATTTGCGTGGAGT   180

ZFX   181  GCGGTAAAGGTTTCGGCACCCGTCAGAGCTCAAAAAGCACATGCGAATCCATACTGGGG   240
           ||||||||||||||| |||||||||||||||| |||||||||||||||||||||||||||
ZFY   181  GCGGTAAAGGTTTCGTCACCCGTCAGAGCTCAGAAAGCACATGCGAATCCATACTGGGG   240

ZFX   241  AGAAGCCGTACCAATGCCAGTACTGCGAATATAGGTCTGCAGACTCTTCTAACTTGAAAA   300
           ||||||||||||||||||||||||| ||||||||||||||||||||||||||||||||||
ZFY   241  AGAAGCCGTACCAATGCCAGTACTGTGAATATAGGTCTGCAGACTCTTCTAACTTGAAAA   300

ZFX   301  CGCATGTAAAAACTAAG   317
           ||||||| |||||||||
ZFY   301  CGCATGTGAAAACTAAG   317
```

*Nsi*I recognition site Restriction site ▲ Diagnostic site ******

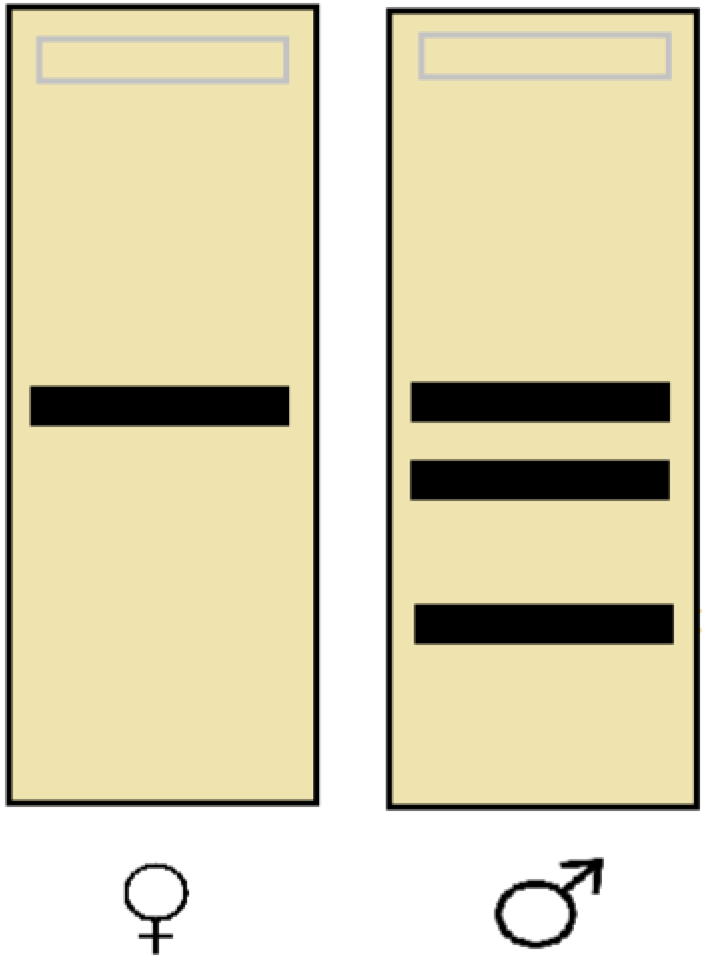

Exact fragment sizes could not be determined.

EMBL acc. No.: AF393753 and AF393754

Asian elephant (*Elephas maximus*). *ZFX, ZFY*

Fernando and Melnick, 2001

F:5'-ATAATCACATGGAGAGCCACAAGCT-3'

R:5'-GCACTTCTTTGGTATCTGAGAAAGT-3'

*Bam*HI recognition site 5'...GGATCC...3' / 3'...CCTAGG...5'

```
ZFX  1    GTGGGAAGCATTTCTCTCACACTGGGGCTTTGTTTACTCACAAAATGGTGCATAAGGAAA  60
          ||||||||||||||||||||||||||||||||||||||||||||||||||||||||||||
ZFY  1    GTGGGAAGCATTTCTCTCACACTGGGGCTTTGTTTACTCACAAAATGGTGCATAAGGAAA  60

ZFX  61   AGGGAGCCAACAAAATGCACAAGTGTAAATTCTGTGAATACGAGACAGCTGAGCAGGGGC  120
          ||||| ||| ||||||||||||||||||||||||||||||||||||||||||||||||||
ZFY  61   AGGGATCCAGCAAAATGCACAAGTGTAAATTCTGTGAATACGAGACAGCTGAGCAGGGGC  120

ZFX  121  TATTGAATCGCCACCTTTTGGCAGTCCATAGCAAGAACTTTCCTCATATTTGTGTGGAGT  180
          ||||||||||||||||||||||||||||||||||||||||||||||||||||||||||||
ZFY  121  TATTGAATCGCCACCTTTTGGCAGTCCATAGCAAGAACTTTCCTCATATTTGTGTGGAGT  180

ZFX  181  GCGGTAAAGGTTTTCGTCACCCTTCAGAGCTGAAAAAGCACATGCGAATCCACACCGGGG  240
          |||||||||||||||||||||||||||||||||||||||||||||| ||||| || ||||
ZFY  181  GCGGTAAAGGTTTTCGTCACCCTTCAGAGCTGAAAAAGCACATGCGGATCCATACTGGGG  240

ZFX  241  AGAAACCCTACCAATGCCAGTACTGCGAATATAGGTCTGCAGACTCTTCTAACTTGAAAA  300
          ||||||||||||||||||||||||||||||||||||||||||||||||||||||||||||
ZFY  241  AGAAACCCTACCAATGCCAGTACTGCGAATATAGGTCTGCAGACTCTTCTAACTTGAAAA  300

ZFX  301  CTCATGTAAAAACTAAGCATAGTAAAGAGATGCCATTCAAGTGTGACATT  350
          ||||||||||||||||||||||||||||||||||||  ||||||||||||
ZFY  301  CTCATGTAAAAACTAAGCATAGTAAAGAGATGCCATATAAGTGTGACATT  350
```

BamHI recognition site

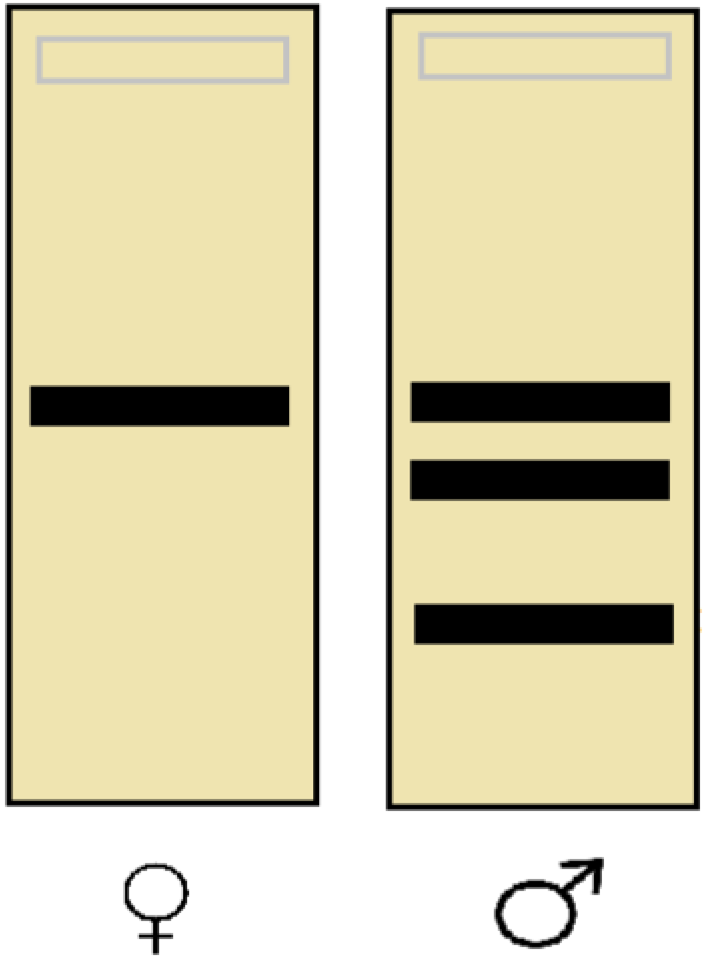

BamHI

EMBL acc. No.: AF393751, AF393752

Exact fragment sizes could not be determined.

Asian elephant (*Elephas maximus*). *ZFX, ZFY*

Vidya et al., 2003

F:5′-ATAATCACATGGAGAGCCACAAGCT-3′

R:5′-GCACTTCTTTGGTATCTGAGAAAGT-3′

*Bam*HI recognition site: 5′...GGATCC...3′ / 3′...CCTAGG...5′

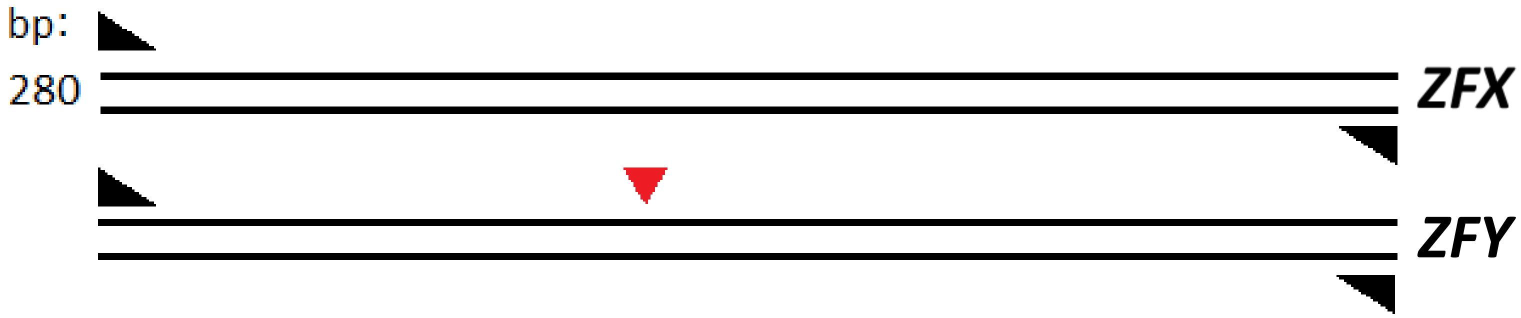

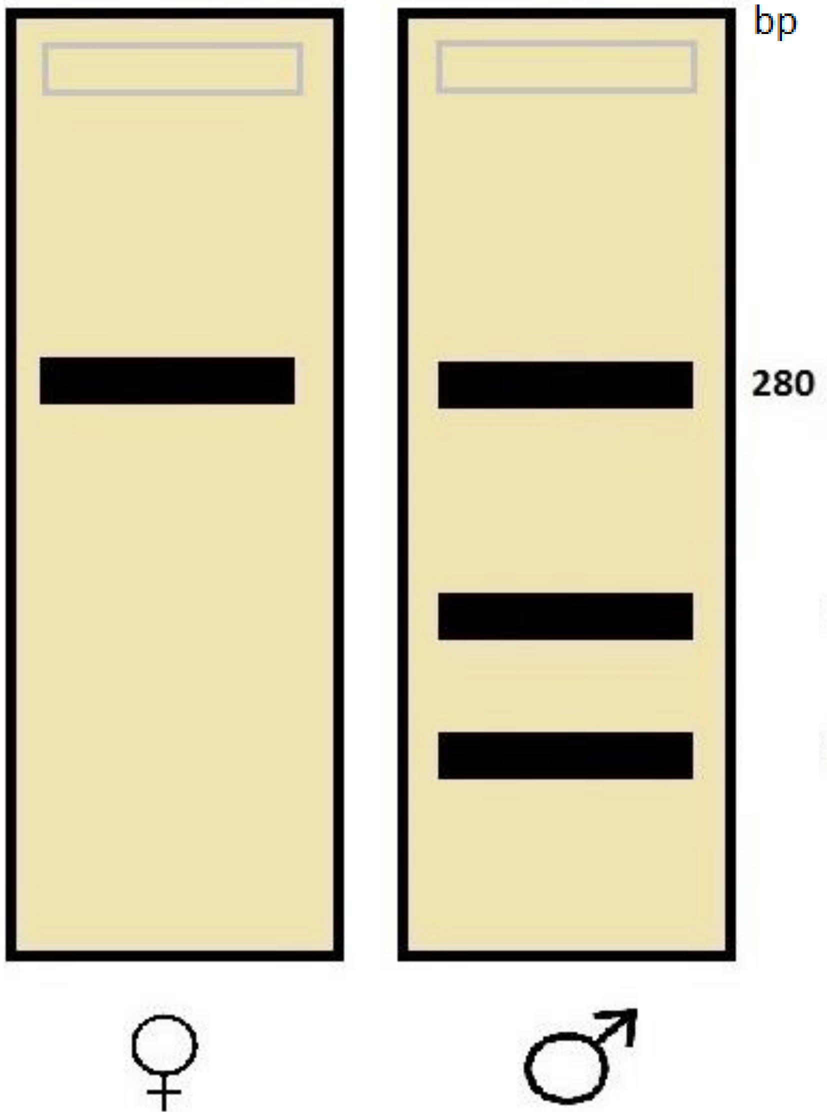

Exact fragment sizes could not be determined.

Maned sloth (*Bradypus torquatus*), Brown-throated sloth (*Bradypus variegatus*), Pale-throated three-toed sloth (*Bradypus tridactylus*), *ZFX, ZFY*

Martinelli et al., 2010

F:5′-ATAATCACATGGAGAGCCACAAGCT-3′

R:5′-GCACTTCTTTGGTATCTGAGAAAGT-3′

*Hae*III restriction site: 5′...GG^CC...3′ / 3′...CC,GG...5′

```
ZFX    1    CATGGAGAGCCACAAGCTAACCAGCAAGGCAGAGAAGGCCATTGAATGTGATGAATGTGG   60
            |||||||||||||||||| |||| ||||||||| |||||||||||||||||||| |||||
ZFY    1    CATGGAGAGCCACAAGCTGACCAACAAGGCAGATAAGGCCATTGAATGTGATGAGTGTGG   60

ZFX    61   GAAGCATTTCTCTCATGCGGGGGCTTTGTTTACTCACAAAATGGTGCATAAGGAAAAAGG   120
            |||||||||||||||  ||||||||||||||||||||||||||||||| |||||||||||
ZFY    61   GAAGCATTTCTCTCACACGGGGGCTTTGTTTACTCACAAAATGGTGCACAAGGAAAAAGG   120

ZFX    121  AGCCAACAAAATGCACAAGTGTAAATTCTGTGAATACGAGACTGCTGAACAAGGGTTACT   180
             |||||||||||||| |  ||||||||||||||||| ||||| ||||||||||||||| |
ZFY    121  GGCCAACAAAATGCATACATGTAAATTCTGTGAATATGAGACCGCTGAACAAGGGTTATT   180

ZFX    181  GAATCGTCACCTTTTGGCAGTCCACAGCAAGAACTTTCCTCATATTTGTGTGGAGTGCGG   240
            || |||||||||||||||||| || |||||||||||||||||||||||||||||||||||
ZFY    181  GAGTCGTCACCTTTTGGCAGTACATAGCAAGAACTTTCCTCATATTTGTGTGGAGTGCGG   240

ZFX    241  TAAAGGTTTTCGTCATCCGTCAGAGCTCAAAAAGCACATGCGAATCCACACTGGGGAGAA   300
            |||||||||||||||||| |||||||||||||||||||||||||||||||| ||||||||
ZFY    241  TAAAGGTTTTCGTCATCCATCAGAGCTCAAAAAGCACATGCGAATCCACACGGGGGAGAA   300

ZFX    301  GCCGTACCAATGCCAGTACTGCGAATATAGATCTGCAGACTCCTCTAACTTGAAAACTCA   360
            |||||||||||||||||||||||||||||| || ||||||||||||||||||||||||||
ZFY    301  GCCGTACCAATGCCAGTACTGCGAATATAGTTCCGCAGACTCCTCTAACTTGAAAACTCA   360

ZFX    361  TGTAAAAACTAAGCATAGTAAAGAGATGCCATTCAAGTGTGACATTTGTCTTCTGACTTT   420
            ||||||||||||||| ||||||||||||||||||||||| ||||||||||||||||||||
ZFY    361  TGTAAAAACTAAGCACAGTAAAGAGATGCCATTCAAGTGCGACATTTGTCTTCTGACTTT   420

ZFX    421  CTC   423
            |||
ZFY    421  CTC   423
```

*Hae*III recognition site

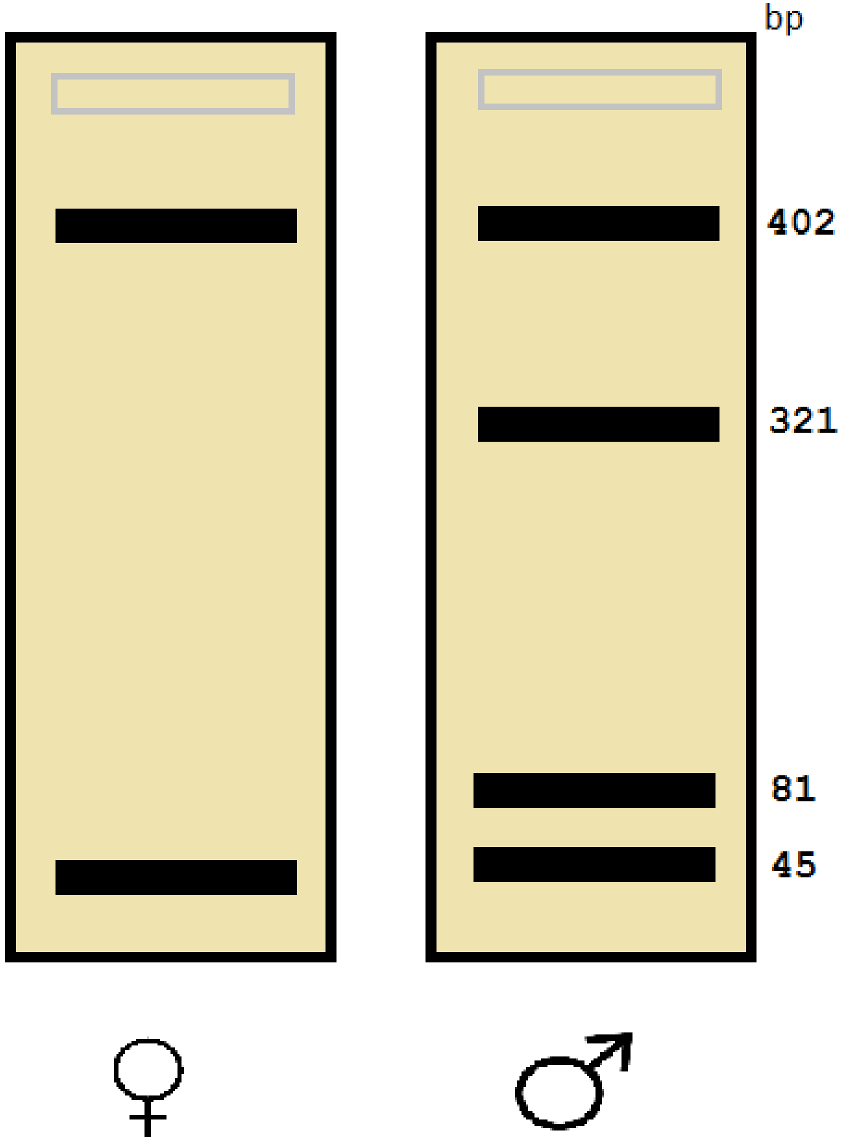

EMBL acc. No.: GU187041, GU187042

Ringtail possums (*Pseudocheirus peregrinus*) and bushtail possums (*Trichosurus vulpecula*). *G6PD, SRY*

Russell et al., 2011

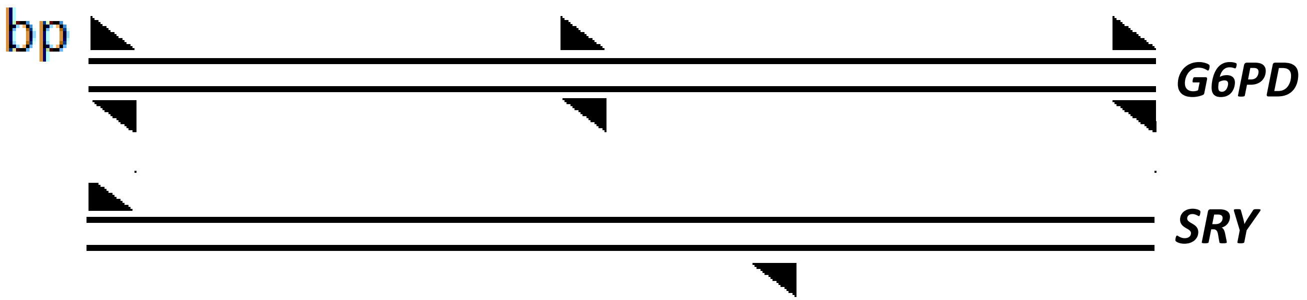

G6PD

F:5′-ACACACATATTCATCAT-3′

R:5′-ATGATGAATATGTGTGT-3′

F:5′-CAGGCCAACCGCCTCTTCTACCTGGCCTTG-3′

R:5′-CAAGGCCAGGTAGAAGAGGCGGTTGGCCTG-3′

F:5′-TTCCAGTATGAGGGCACCTACAAGTGG-3′

R:5′-CCACTTGTAGGTGCCCTCATACTGGAA-3′

SRY

F:5′-GCTATGTATGGCTTCTTGAATG-3′

R:5′-GTTAAACCTGAAACGAATGACAG-3′

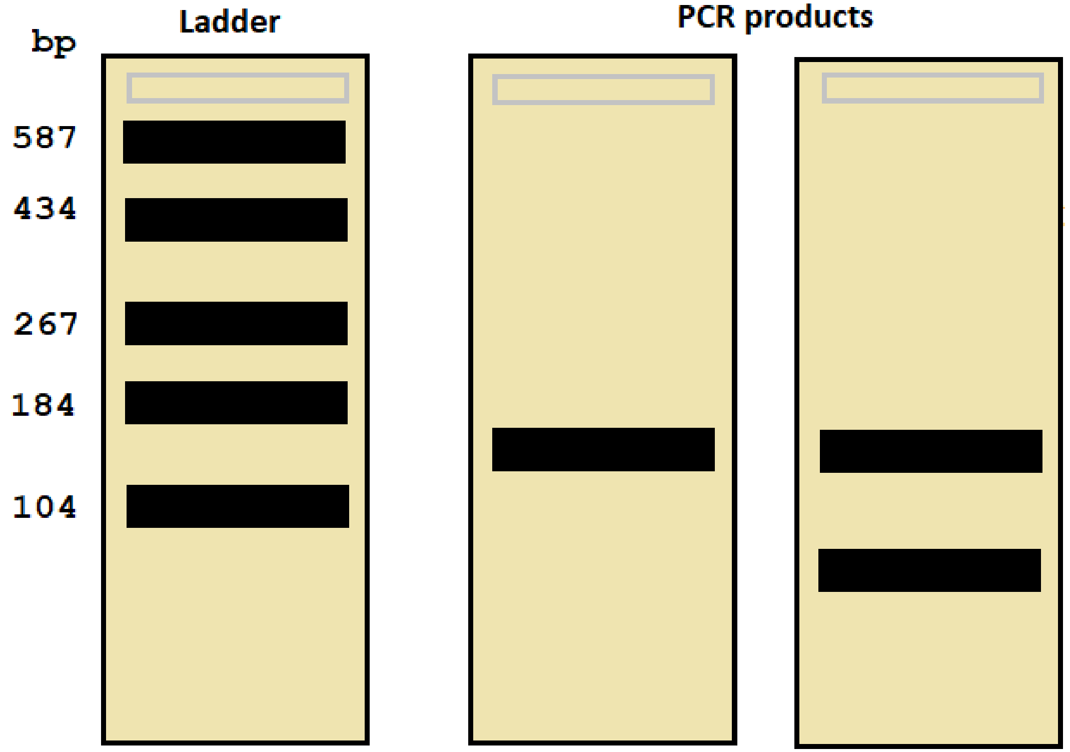

Exact fragment sizes could not be determined. ♀ ♂
